# Bonding and reactivity of isostructural uranyl and neptunyl peroxide phases

**DOI:** 10.1038/s42004-025-01733-6

**Published:** 2025-11-22

**Authors:** Harindu Rajapaksha, Grant C. Benthin, Emma L. Markun, Cameron J. Flester, Sara E. Mason, Tori Z. Forbes

**Affiliations:** 1 Department of Chemistry, University of Iowa, Iowa City, IA USA; 2 Center for Functional Nanomaterials, Brookhaven National Laboratory, Upton, NY USA

**Keywords:** Chemical bonding, Coordination chemistry

## Abstract

Understanding the reactivity of actinide peroxides is critical for predicting the behavior of spent nuclear fuel in radiolytic environments. Herein, we report the synthesis and characterization of a lithium neptunyl(VI) hydroxo peroxo compound (**LiNp**), which is isostructural to the uranyl analogue (**LiU**). Single-crystal X-ray diffraction reveals that **LiNp** contains both [NpO_2_(O_2_)_3_]^4-^ and [NpO_2_(OH)_4_]^2-^ units stabilized by Li^+^ and hydrogen bonding and Raman spectroscopy shows systematic redshifts in neptunyl vibrational modes relative to uranyl. DFT calculations highlight the importance of secondary coordination in reproducing vibrational and structural features, but challenges remain with correctly modeling strong sigma donors. Solid-state EPR spectroscopy and DFT confirm superoxide stabilization within **LiU** and calculations suggest favorability of the analogous radical species in **LiNp**. Solution state EPR spectroscopy with the radical spin trap 5-tert-butoxycarbonyl-5-methyl-1-pyrroline N-oxide (BMPO) reveal evidence of superoxide in the **LiU** and **LiNp** phases and suggests stabilization of superoxide within actinyl triperoxide complexes, forming [AnO_2_(O_2_)_2_(O)_2_^•^]^3-^.

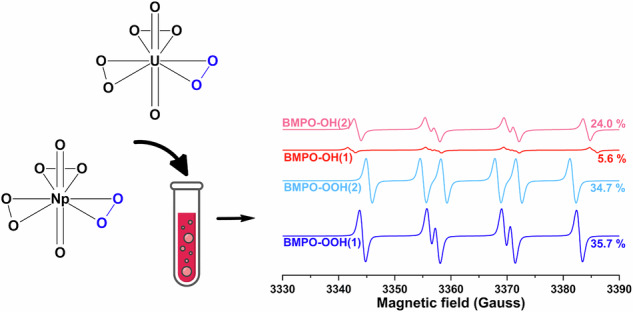

## Abstract (web summary)

Understanding the reactivity of actinyl-peroxide complexes is critical for predicting the behavior of spent nuclear fuel in radiolytic environments. Here, the authors synthesize and characterize a lithium neptunyl(VI) hydroxo-peroxo phase that stabilizes superoxide and underscores the importance of secondary-sphere coordination in modeling actinyl–peroxide compounds.

## Introduction

High ionizing radiation fields produced from nuclear materials can lead to the formation of free radicals and reactive species that further complicate the already complex chemical processes in the nuclear fuel cycle. Within aqueous solutions, ionizing radiation will generate water radiolysis products, including ${{e}^{-}}_{({{\rm{aq}}})}$, ${{{\rm{H}}}}_{({{\rm{aq}}})}^{\bullet }$, ${{{\rm{OH}}}}_{({{\rm{aq}}})}^{\bullet }$, peroxides (${{{\rm{H}}}}_{2}{{{\rm{O}}}}_{2({{\rm{aq}}})}$, ${{{\rm{HO}}}}_{2({{\rm{aq}}})}^{-},\,{{{\rm{O}}}}_{2({{\rm{aq}}})}^{2-}$), and superoxide (${{{\rm{HO}}}}_{2({{\rm{aq}}})}^{\bullet },\,{{{\rm{O}}}}_{2({{\rm{aq}}})}^{{{\bullet }}-}$) species^1–4^. These reactive ions and molecules can impact the nuclear fuel cycle processes by corroding UO₂ fuel pellets^5,6^, degrading organic extractants used in radiological separation^7^, and promoting the formation of secondary alteration products on the surface of nuclear waste forms^8–10^. These alteration phases increase the risks of storing and handling spent nuclear fuel by forming more reactive actinide-bearing compounds^11,12^.

Among the reactive species produced by water radiolysis, the peroxide anion (O_2_^2−^) is the most relevant for chemical timescales and has been previously identified as an important species throughout the fuel cycle. Peroxide is added as an oxidant during the milling process, and U(VI) peroxides are often a starting material for the production of yellowcake^13,14^. On the back end of the fuel cycle, U(VI) peroxides, studtite ([UO_2_O_2_(H_2_O)_2_] • 2 H_2_O), and meta-studtite ([UO_2_O_2_(H_2_O)_2_]), have been identified as corrosion products formed on the surface of nuclear fuel under aqueous conditions^15–17^. In addition, reprocessing schemes, such as the CARBEX process, rely on the formation of peroxo and peroxocarbonate species under alkaline conditions for oxidative dissolution of the uranium solids^18^. Under these conditions, a range of actinide species are predicted to form, including coordination complexes ([AnO_2_(O₂)₃]⁴⁻) and larger peroxo clusters^19–21^.

While much of the research on actinide peroxide chemistry has focused on uranium, it is equally important to understand the behavior of other actinides, particularly neptunium-237 (^237^Np) in similar oxidative environments.^237^Np is a minor actinide generated in nuclear reactors primarily through two pathways: ${(1)}_{92}^{238}{{\rm{U}}}(n,2n)_{92}^{237}{{\rm{U}}}\to _{93}^{237}{{\mathrm{Np}}}+_{-1}^{0}\beta +{\overline{\nu }_{e}}$ and ${(2)}_{92}^{235}{{\rm{U}}}\left(n,\gamma \right)_{92}^{236}{{\rm{U}}}(n,\gamma )_{92}^{237}{{\rm{U}}}\to _{93}^{237}{{\mathrm{Np}}}+_{-1}^{0}\beta +{\overline{\nu }_{e}}$. Due to its long half-life (~2.14 × 10⁶ years), ^237^Np is a significant contributor to the long-term radiotoxicity of spent nuclear fuel and nuclear waste^22–25^. It is also used as a precursor to produce ^238^Pu, a radionuclide widely used in radioisotope thermoelectric generators for space missions^26^. Despite its significance, the coordination chemistry of neptunyl species under oxidizing conditions, particularly in the presence of water radiolysis products such as peroxide, remains poorly understood.

To date, only two neptunyl peroxide compounds have been structurally characterized. The first, synthesized by Burns et al. in 2005, was a nanocluster (Np-24) with the composition Li_20_[Li_6_(H_2_O)_8_NpO_2_(H_2_O)_4_(NpO_2_(O_2_)(OH))_24_] that was isolated from a solution containing H_2_O_2_ and LiOH^27^. The Np-24 nanocluster is composed of neptunyl hydroxo peroxo complexes linked together to form a sphere containing 24 Np(VI) cations. An additional [NpO_2_(H_2_O)_4_]^2+^ moiety was reported to exist in the center of the Np-24 nanocluster, but the observed bond lengths and geometry suggest it could alternatively be identified as a [NpO_2_(OH)_4_]^3−/2−^ unit^28^. The second known neptunyl peroxide compound was isolated by Hickam et al. in 2019 and contains isolated units of [NpO_2_(O_2_)_3_]^4−^ charge balanced by Ca^2+^ cations^29^. Raman spectroscopy was also collected for the Ca_2_[NpO_2_(O_2_)_3_]∙9 H_2_O phase and included several features between 600–750 and 800–850 cm^−1^ that corresponded to neptunyl and peroxide vibrational modes, respectively. To date, there are no structurally characterized Np(IV) or Np(V) peroxide compounds, and only a few peroxide compounds containing Th(IV) and Pu(IV) have been reported. A detailed discussion of the tetravalent actinide peroxo complexes is beyond the scope of this study, but we encourage readers to refer to the review by Kruse et al.^30^ for a condensed overview, along with the primary literature of Margate et al.^31^ and Runde et al.^32^ on Pu(IV) peroxide chemistry, and Bonato et al.^33^ and Galley et al.^34^ on Th(IV) peroxide chemistry. While these two studies provided a structural basis for the Np(VI) peroxide coordination complexes, there is still limited information on the overall chemistry of this system. This highlights a critical need to explore the chemistry of neptunyl peroxides, giving special attention to their chemical reactivity.

Herein, we report the synthesis and isolation of a lithium neptunyl hydroxo peroxo phase (**LiNp**), which is isostructural to its uranyl analog (**LiU**)^35^, providing a unique platform for comparing structure, bonding, vibrational modes, and reactivity. The structure of **LiNp** was determined by single-crystal X-ray diffraction and Raman spectroscopy. Supporting Density Functional Theory (DFT) calculations were used to probe the vibrational features and bonding in both phases. Solid-state electron paramagnetic resonance (EPR) spectroscopy was performed to investigate the reactivity of peroxide to form oxygen-centered radicals in **LiU**. Additionally, solution-state EPR spectroscopy with 5-tert-butoxycarbonyl-5-methyl-1-pyrroline N-oxide (BMPO) spin traps was used to further investigate the presence of reactive oxygen species within **LiU** and **LiNp**.

## Results and discussion

### Characterization of the Np(VI) peroxide solid

When the Np(VI) stock solution was added to a mixture of LiOH and H₂O₂, the color changed from light red to deep dark red, indicating that peroxide likely formed a complex with the Np(VI) ion. While the Np(VI) stock solution contains features in the NIR region (1226 nm, Supporting information (SI) Section 2.1, Fig. S5), the UV/Vis/NIR spectrum of the Np(VI) peroxide solution in LiOH only displays a weak, broad feature centered at 600 nm (SI Section 2.1, Fig. S5). The Raman spectrum of the solution exhibits multiple features characteristic of peroxide species, including an intense band at 850 cm^−1^ attributed to the free OOH^−^ (SI Section 2.1, Fig. S5d). The final pH of the solution is >12. Since this value exceeds the first pK_a_ of H_2_O_2_, the acid-base equilibrium favors deprotonation, shifting toward OOH^−^ making it the dominant species. To confirm this assignment, a reference Raman measurement of H_2_O_2_ in 2 M KOH was performed, which exhibited a peak at 848 cm^−1^. This is within instrumental error of the observed value and supports the assignment of the 850 cm^−1^ band to free OOH^−^ (SI Section 5, Fig. S22). Additionally, a weaker band at 815 cm^−1^ is consistent with the presence of peroxide (O_2_^2−^) coordinated to the Np(VI) center. The feature at 704 cm^−1^ can be assigned to the symmetric stretching vibration of the neptunyl unit in the [NpO_2_(O_2_)_3_]^4−^ complex and is supported by solid-state data (Table 1). The band at 666 cm^−1^ falls within the expected range for the symmetric stretching mode of neptunyl in neptunate(VI) compounds, which are known to form under highly alkaline conditions. Additionally, the presence of multiple bands in the 500–300 cm^−1^ (SI Section 2.1, Fig. S5d) region further supports the formation of neptunate(VI) species, as these compounds typically exhibit several Raman-active modes in this spectral range^36^.

**Table 1:** Summary of the LiU and LiNp solid-state Raman peak assignments

LiU peak centroids (cm^−1^)	LiNp peak centroids (cm^−1^)	Peak shift (LiU-LiNp) (cm^−1^)	Peak assignment
1090	1089	1	CO_3_^2−^ symmetrical stretching of the C-O bond
873	874	−1	Actinyl cation coordinated O_2_^2−^ in-phase symmetrical stretching (ν_1)_
844 and 816	843 and 807	1 and 9	Actinyl cation coordinated O_2_^2−^ out-of-phase symmetrical stretching (ν_2_ and ν_3_)
798	747	51	[AnO_2_(OH)_4_]^2−^ actinyl cation symmetrical stretching (ν_1_)
722	705	17	[AnO_2_(O_2_)_3_]^4−^ actinyl cation symmetrical stretching (ν_1_)
488, 391, and 309	471, 387, and 347	17, 4, and −38	AnO_2_^2+^- O_2_^2−^ stretching modes

**Fig. 1:**
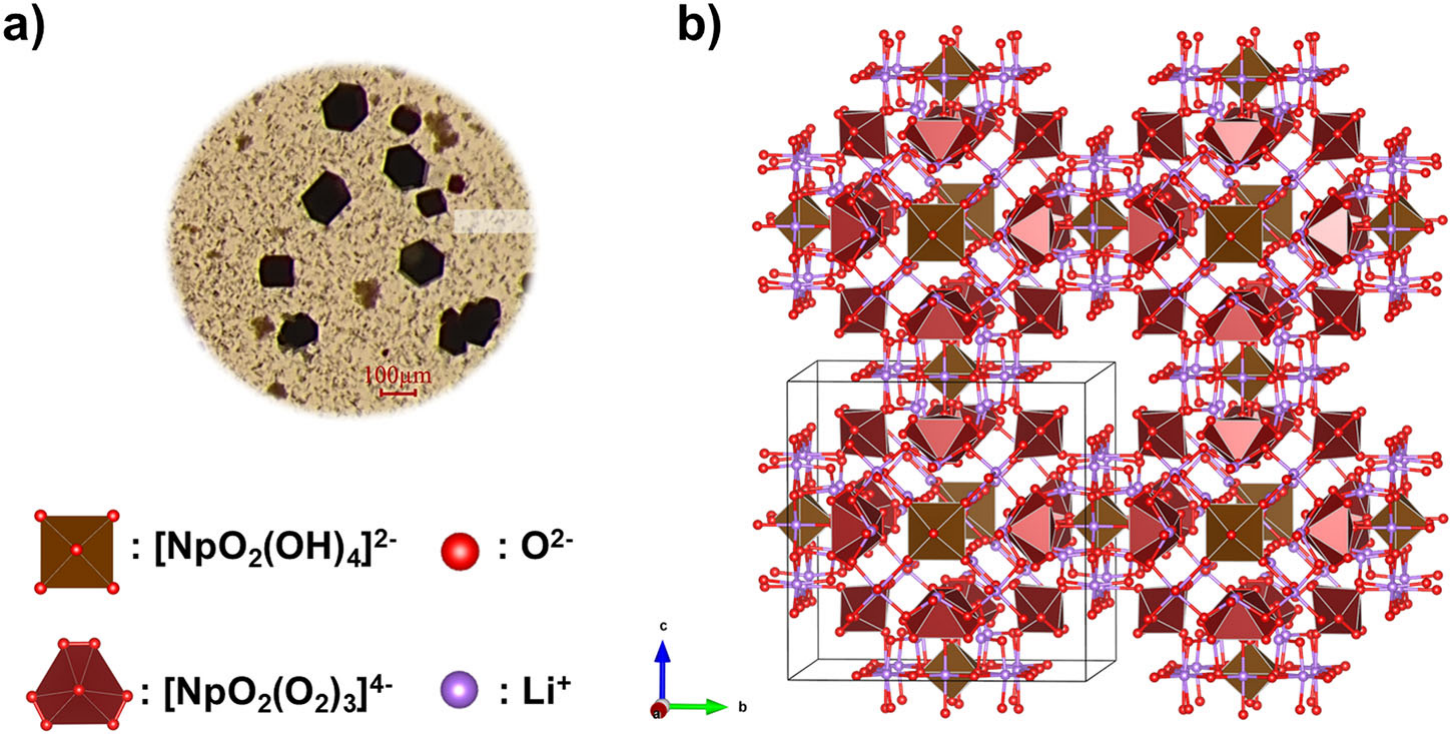
Optical microscopy and the crystal structure of LiNp. **a** Optical microscope image of the product shows the formation of a blocky, dark-red crystalline phase (**LiNp**). **b** Structural characterization of **LiNp** indicates that the material is composed of [NpO_2_(O_2_)_3_]^4−^ and [NpO_2_(OH)_4_]^2−^ coordination complexes that are charge balanced with Li^+^ cations.

Upon addition of methanol as a crystallization agent, large (100 μm), blocky crystals (**LiNp**) with good yields (Fig. 1a) were obtained and structurally characterized using single-crystal X-ray diffraction. The compound crystallizes in a cubic *Pm*-3*m* space group, and the unit cell contains twelve [NpO_2_(O_2_)_3_]^4−^ and three [NpO_2_(OH)_4_]^2−^ units with all Np atoms in VI oxidation state. The overall charge balance is achieved by the Li^+^ cations (Fig. 1b). The Np=O bond length within the [NpO_2_(OH)_4_]^2−^ moiety (1.79(1) Å) fits with the expected value reported by Clarke et al., whereas the neptunyl bond distance observed in the [NpO_2_(O_2_)_3_]^4−^ complex is longer (1.834(4) Å), but consistent with previously reported neptunyl triperoxide complex (1.843(7) Å)^21,29,37^. This bond elongation likely results from significant electron donation from the peroxide ligands to the actinyl center, which increases the Lewis basicity of the axial oxygen atoms, enhancing their ability to engage in actinyl–cation interactions^38,39^. In the crystal structure, each axial oxygen in [NpO_2_(O_2_)_3_]^4−^ interacts with three Li^+^ ions, with average Li–O distances of 2.266 Å, and each Np(VI) triperoxide complex is surrounded by twelve water molecules, hydrogen-bonded to the peroxide ligands. Additional water molecules are located within the interstitial regions to give an overall formula of (Li_18_[NpO_2_(O_2_)_3_]_4_[NpO_2_(OH)_4_]∙39.5 H_2_O).

The **LiNp** phase is isostructural to a uranyl peroxo hydroxo phase previously reported by the Nyman group (Table 2)^35^. While the bond distances observed in the [AnO_2_(OH)_4_]^2−^ units are consistent with expected changes, subtle differences can be observed for the [AnO_2_(O_2_)_3_]^4−^ complex. The NpO_2_^2+^ cation has one additional electron compared to UO_2_^2+^, which occupies a non-bonding *f* orbital on the actinyl center. This added electron can repel the negatively charged axial oxo ligands, and as a result, Np(VI)=O bond lengths are generally expected to be longer than the corresponding U(VI)=O bonds. However, in the [AnO_2_(O_2_)_3_]^4−^ unit of **LiNp**, the Np(VI)=O bond is observed to be 0.016 Å shorter than the U(VI)=O bond in the analogous **LiU** complex. The An-O equatorial distances are within error, but the peroxo bonds in the Np(VI) compound are 0.034 Å shorter than the distance observed in the U(VI) compound. We also note that all Li^+^ cation interactions with the neptunyl oxo atoms are slightly longer than those observed in the related U(VI) compound, suggesting weaker second-sphere electrostatic interactions. However, resolving Li^+^ cation positions using XRD can be challenging, particularly in a high symmetry unit cell, and may not provide an accurate assessment of the extended coordination environment.

**Table 2:** Summary of bond lengths (Å) and interaction distances (Å) associated with the uranyl and neptunyl hydroxo peroxo compounds

Bond length Å		LiU^a^	LiNp
[AnO_2_(O_2_)_3_]^4−^	An=O (actinyl)	1.850(8)	1.834(4)
	An-O_2_ (actinyl peroxide)	2.281(8) 2.310(9) 2.327(9)	2.281(4) 2.301(4) 2.325(4)
	O-O (peroxide)	1.49(1) 1.51(2)	1.497(6) 1.476(8)
	An=O--Li^+^	2.200(8) 2.25(4) 2.25(4)	2.237(4) 2.28(2) 2.28(2)
[AnO_2_(OH)_4_]^2−^	An=O (actinyl)	1.77(4)	1.79(1)
	An-OH (actinyl hydroxide)	2.29(3)	2.23(1)
	An=O--Li^+^	1.93(6)	1.94(2)

^a^Bond distances for **LiU** were taken from ref. ^19^. Crystallographic parameters for LiNp can be found in the SI, Table S2.

**Fig. 2:**
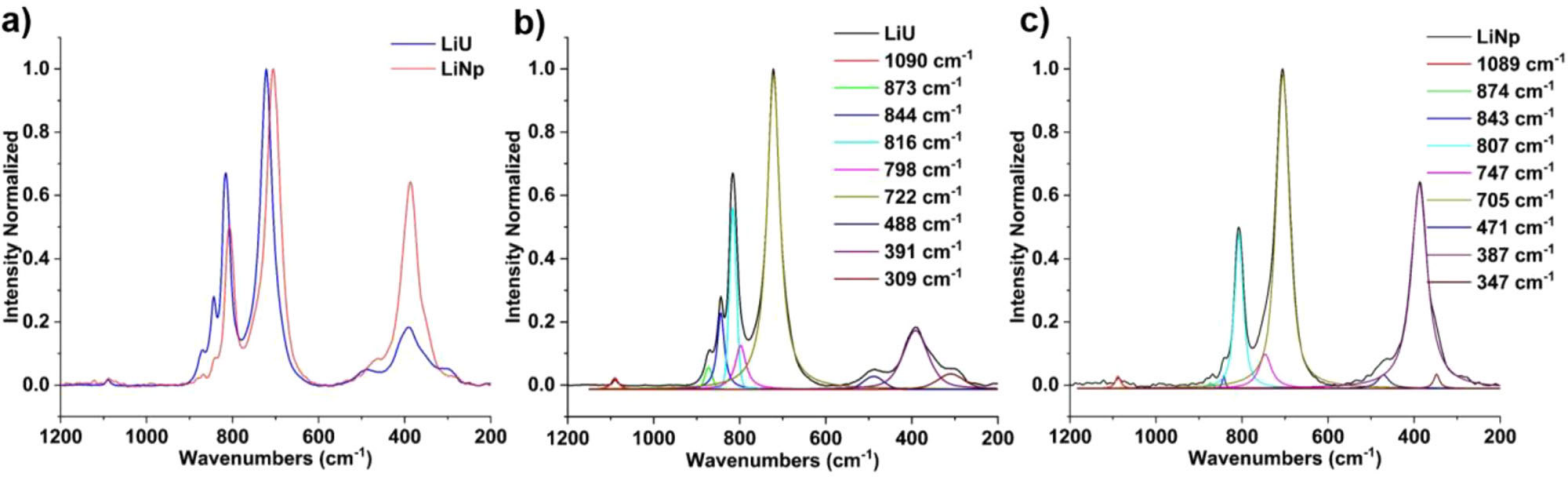
Solid-state Raman spectra of LiU and LiNp. **a** The stacked Raman spectra of **LiU** (blue) and **LiNp** (red) emphasize the red shift of the neptunyl peroxide bands. Peak fittings of **b** **LiU** and **c** **LiNp** solid phases provide a quantitative assessment of the peak shifts between the isomorphous solids. Spectral intensities are normalized with respect to the maximum intensity peak to aid in comparison between the two compounds.

To gain deeper insight into the bonding characteristics of the material, we employed Raman spectroscopy (Fig. 2). The AnO_2_^2+^ cation has four fundamental vibrational modes: symmetric stretch (Raman-active ν_1_), doubly degenerate bending mode (IR active ν_2_), and antisymmetric stretch (IR active ν_3_)^40,41^. Additional vibrational features can appear from ligand vibrations and AnO_2_^2+^-ligand stretching modes. Neptunyl vibrational bands generally exhibit a red shift compared to their uranyl counterparts. This shift is attributed to a weakened actinyl bond resulting from increased electron density at the neptunium center and enhanced non-covalent interactions involving the axial oxygens in neptunyl complexes^37,42,43^.

For the **LiNp** phase, the neptunyl symmetric stretch (ν₁) associated with the [NpO_2_(O_2_)_3_]^4−^ complex is observed at 705 cm^−1^, representing a red shift of 17 cm^−1^ relative to the corresponding ν₁ band of [UO_2_(O_2_)_3_]^4−^ in **LiU** (Fig. 2a, b, Table 1). Based on crystallographic data, one might expect the ν₁ band of [UO_2_(O_2_)_3_]^4−^ to appear at lower energy than that of the neptunyl analog due to the longer U=O bond length (1.850 Å)^35^ compared to Np=O (1.834 Å). This trend is also observed for the Ca_2_[AnO_2_(O_2_)_3_] system as the An=O bond distances are within error, but the Raman-active Np=O ν_1_ band is 20 cm^−1^ red-shifted compared to the U(VI) analog^21,29^. These discrepancies highlight that crystallographic data alone are insufficient for reliably predicting vibrational bands within actinyl solids, particularly for peroxide complexes^42^.

Other features present in the Raman spectra match well with previously reported compounds. Vibrational modes located at 798 and 747 cm^−1^ in the **LiU** and **LiNp** spectra, respectively, can be linked to the [AnO_2_(OH)_4_]^2−^ complex as Clark et al. previously reported uranyl and neptunyl symmetric stretching vibrations for these species at 796 and 741 cm^−1^, respectively^28,44^. Vibrational bands of peroxide bound to actinyl cation are observed at 873–874 cm^−1^ (ν_1_), 843–844 cm^−1^ (ν_3_), and 807–816 cm^−1^ (ν_2_), and they match well with literature values^21,29^. Additional peaks in the 300–600 cm^−1^ range can be attributed to AnO_2_^2+^-peroxide stretching modes based upon previous work by Margate et al.^31^. A weak feature at 1089–1090 cm^−1^ present in both spectra is attributed to residual carbonate on the surface of the crystals, potentially formed from the reaction between CO_2_ and hydroxide. This reaction can occur either in the LiOH stock solution or during the crystal isolation from the mother liquor.

DFT calculations were utilized to further confirm the Raman-active vibrational modes and bond distances (SI, Tables S4 and S5). Significant discrepancies were observed between the calculated and experimental results, consistent with similar findings previously reported by others^21,29^. It is important to note that the theoretical vibrational frequency calculations presented here are based on the harmonic oscillator approximation; therefore, some discrepancy between experiment and theory is expected. However, previous DFT studies on Raman features of other molecular actinyl systems (e.g. [NpO_2_Cl_4_]^2−^) have demonstrated greater agreement with experimental data compared to the triperoxide unit discussed in this work^38,45,46^.

For the [AnO_2_(O_2_)_3_]^4−^ unit, calculations using the bare actinyl–peroxide complex (Model I) overestimate the bond distances by 0.04–0.05 Å and underestimate the actinyl ν₁ band by 76 and 50 cm^−1^ for U and Np, respectively. Prior work noted the importance of the second coordination sphere to correctly predict the band positions^42,43^, so we developed three additional models for the [AnO_2_(O_2_)_3_]^4−^ unit and one for the [AnO_2_(OH)_4_]^2−^ species, each including explicit water molecules and Li^+^ cations. When two Li^+^ cations each engaging in actinyl–cation interactions are included in the calculations (Model II), we find significant improvements in experimental agreement to the ν_1_ actinyl symmetric stretch, reducing the discrepancies to 5 and 7 cm^−1^ for U(VI)O_2_^2+^ and Np(VI)O_2_^2+^, respectively. However, the opposite trend is observed for the peroxide vibrational modes within these systems. Model I yields lower absolute average errors of 8 and 19 cm^−1^ for the ν_1_ O_2_^2−^ stretching mode in the U(VI) and Np(VI) compounds, respectively, while the inclusion of secondary interactions in Model II increases these errors to 44 and 40 cm^−1^. For bare [UO_2_(OH)_4_]^2−^ and [NpO_2_(OH)_4_]^2−^ complexes, the calculated actinyl ν₁ bands are underestimated by 32 and 12 cm^−1^, and An=O bond distances are overestimated by 0.07 and 0.02 Å, respectively. Adding the second coordination sphere improves the U=O bond substantially, but the calculated actinyl ν₁ bands are now overestimated by 63 cm^−1^. For [NpO_2_(OH)_4_]^2−^, inclusion of the second coordination sphere leads to underestimation of the Np=O bond by 0.04 Å and overestimation of the ν₁ mode by 108 cm^−1^.

To further investigate these discrepancies in actinyl bonding, QTAIM^47^ analysis was performed at the Bond Critical Points (BCPs). The electron density at the BCP of the Np=O bonds in both the [AnO_2_(O_2_)_3_]^4−^, and [AnO_2_(OH)_4_]^2−^ was found to be higher than that of the corresponding U=O bonds (SI Section 6.5, Tables S8 and S9), suggesting a stronger neptunyl bond. However, this finding appears inconsistent with the Raman spectroscopic data, which indicates a weaker Np=O bond. This highlights the challenges in reconciling localized, equilibrium electron density descriptors with vibrational observables. Specifically, QTAIM analysis reflects the ground-state electron density at the equilibrium geometry and does not account for dynamic changes in charge distribution induced by nuclear motion. Vibrational modes can perturb the electronic structure in ways that alter bond strengths or polarizabilities, effects that may not be captured in static, topological analyses of the electron density.

To confirm that we had chosen the best methodology for the peroxide system, we benchmarked 22 density functionals for geometry optimization and vibrational frequency calculations of the [NpO_2_(O_2_)_3_]^4−^ unit (SI, Section 1.6, Figs. S1–S4). The results indicate that no single functional accurately reproduces both the experimental structural parameters of the NpO_2_^2+^ core and the peroxide ligands simultaneously. Functionals such as TPSS0^48^ and ωr2SCAN^49^ provide highly accurate results for the actinyl cation, whereas functionals like TPSS^50^ and RPBE^51^ yield better agreement with the experimental data for the peroxide ligands. These results suggest that the overall limitations of DFT in this context may not stem solely from the omission of secondary coordination effects, but rather from an overestimation of the sigma bond strength in these strong donors. For the [AnO_2_(O_2_)_3_]^4−^, DFT may overestimate the An–peroxide bond strength, which in turn leads to an overestimation of electron density at the metal center and an artificial weakening of the actinyl bond^37,52^. The interplay of DFT delocalization error and the strong σ-donor character of peroxide ligands manifests in the difficulty describing mixed bonding regimes within a single functional framework. Systematic benchmarking against experimental structural and spectroscopic data provides essential feedback to guide and support the need to develop more accurate and transferable methods for complex actinide systems.

### Actinyl peroxide radical chemistry

Peroxide is considered part of the family of reactive oxygen species and can readily oxidize or degrade to form radical species. Previous work by Scherrer et al. demonstrated that over the course of several days, the U(VI) peroxide solid K_4_[UO_2_(O_2_)_3_] dissolved in water would react to form U(VI) superoxide species^53^. This was also shown to occur in the solid state by both Scherrer et al.^53,54^ and Kravchuk et al.^11^, where EPR spectroscopy confirmed the presence of superoxide in U(VI) peroxide compounds. No reactivity studies have been conducted for the Np(VI) peroxide phases; thus, we utilized EPR spectroscopy and DFT calculations for both the **LiU** and **LiNp** systems to provide additional information on their chemical behavior.

**Fig. 3:**
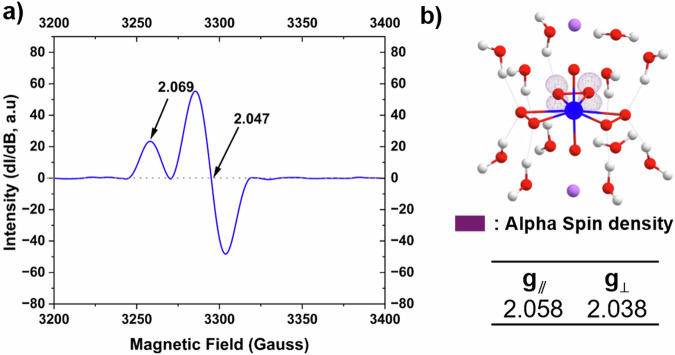
Solid-state EPR spectra and calculated EPR parameters of LiU. **a** Solid-state EPR spectrum of the uranyl phase at room temperature and **b** spin density showing the formation of superoxide within [UO_2_(O_2_)_2_(O)_2_^•^]^3−^ unit. The spin density is generated with iso value of 0.015. Blue, red, purple, and white spheres represent U, O, Li, and H, respectively.

EPR spectroscopy was first used to evaluate the presence of superoxide or hydroxyl radicals in the U(VI) solid-state material. Solid-state EPR spectra of the **LiU** compound exhibited an axial signal with *g*_||_ = 2.069 and *g*_⊥_ = 2.047 (Fig. 3a). In the solid state, superoxide commonly exhibits an axial EPR signature (*g*_z_ ≠ *g*_x_ = *g*_y_), with reported *g*_||_ values ranging from 2.034 to 2.168 and *g*_⊥_ values from 2.005 to 2.075^55–58^. In previous work by the Forbes group, we measured *g*_||_ and *g*_⊥_ values for superoxide coordinated to a uranyl cation at 2.035–2.050 and 2.015–2.017, respectively^53,54^. The *g* value for the unpaired electron within an atomic or molecular orbital typically deviates from the free electron (2.0023)^59^ due to factors such as spin–orbit coupling, hyperfine interactions, and orbital interactions^60,61^. It is also highly sensitive to the electronic structure and local environment of the radical species and can be influenced by secondary interactions such as hydrogen bonding and cation interaction, making it characteristic of both the chemical species and its surrounding matrix^62–64^. This is clearly observed for **LiU**, which exhibits a shift in the EPR signal to a higher magnetic field compared to the previously observed signals in the related potassium U(VI) complex.

DFT calculations were previously used to interpret the EPR signatures of radical complexes^53,54,65^, so we utilized this methodology to determine the origin of the observed solid-state EPR features within the **LiU** solid. The crystal structure contains U(VI) peroxide and hydroxide complexes, suggesting the potential presence of either a superoxide or hydroxyl radical species. Formation of these radicals requires the removal of an electron from the respective ligand (O_2_^2−^ → O_2_^−•^ or OH^−^ → ^•^OH); thus, electrons were removed from the [UO_2_(O_2_)_3_]^4^^−^ and [UO_2_(OH)_4_]^2^^−^ units, generating the open-shell [UO_2_(O_2_)_2_(O)_2_]^3•^^−^ and [UO_2_(OH)_4_]^•^^−^ species in the simulation. For the [UO_2_(O_2_)_3_]^4^^−^, complex, the HOMO to HOMO-2 molecular orbitals are primarily composed of peroxide π* character (SI, Section 6.4, Fig. S25). Removal of an electron to form O_2_^−•^ is expected to occur from these orbitals^66^ and this is observed in the spin density distribution of the optimized [UO_2_(O_2_)_2_(O)_2_^•^]^3^^−^ structure, where the unpaired spin is localized on the 2p_z_ orbitals of the two oxygen atoms within a single ligand (Fig. 3b). The calculated *g*_||_ and *g*_⊥_ values for the bare [UO_2_(O_2_)_3_]^4^^−^ model (Model I) were 2.063 and 2.018, respectively. Upon inclusion of the secondary coordination sphere (Models II–IV, Supporting Information Section 5.2, Table S3), the calculated values shifted to 2.055–2.058 (*g*_||_) and 2.036–2.038 (*g*_⊥_), bringing them into closer agreement with experimentally observed values. In contrast, the spin density of [UO_2_(OH)_4_]^•−^ is not purely ligand-centered but is delocalized across the entire molecule. This is consistent with the nature of the HOMO in [UO_2_(OH)_4_]^2^^−^, which is not strictly ligand-based. The calculated *g*-tensor for this species is rhombic, with values of *g*_x_ = 1.230, *g*_y_ = 1.629, and *g*_z_ = 1.986 (SI, Section 6.2, Table S5), which significantly deviate from experimentally observed values. Furthermore, when the secondary coordination sphere was included (Model VI), the spin density localized predominantly on surrounding water molecules, while the [UO_2_(OH)_4_]^2^^−^ unit remained closed-shell (Supporting Information Section 5.4, Fig. S27c). This suggests that electron removal from a water molecule in the crystalline lattice is more favorable than from the hydroxide-bound uranyl complex. Taken together, these results strongly indicate that stabilization of a hydroxyl radical in the uranyl hydroxide unit is unlikely. Instead, the species detected experimentally is best assigned as a superoxide radical coordinated within the [UO_2_(O_2_)_2_(O)_2_^•^]^3^^−^ unit.

Solid-state EPR measurements for the solid neptunyl phase were not pursued due to radiological safety concerns. Instead, DFT calculations provided some additional insights into the system. In the [NpO_2_(O_2_)_2_(O)_2_^•^]^3^^−^ unit where the spin multiplicity is three, the spin density is not confined to an equatorial ligand but is distributed between the metal center and a superoxide ligand located in the equatorial plane. Here, one unpaired electron resides on the 2p_z_ orbital of the superoxide, while the other occupies the *δ*_u_ orbital of the neptunyl cation. The calculated g-tensor values (*g*_x_ = 0.656, *g*_y_ = 1.244, and *g*_z_ = 1.528, Model I) are markedly different from those of the uranyl superoxide species. This enhancement in anisotropy and deviation from the free-electron value stems from the metal-centered unpaired electron (higher spin-orbital coupling) and the zero-field splitting intrinsic to triplet systems. Comparable shifts in *g* values have been reported for actinide complexes with metal-localized spin densities^67–70^. DFT calculations also provided information on the relative stability of [NpO_2_(O_2_)_2_(O)_2_^•^]^3^^−^ and [UO_2_(O_2_)_2_(O)_2_^•^]^3^^−^ within the crystal lattice (Models II–VI) suggest that both species have similar thermodynamic stabilies with Δ*G* = −11.93 to 6.34 kJ/mol (SI, Section 6.2, Table S7). This result supports the idea that Np(VI) superoxide complexes could be potentially stabilized in the solid-state material.

Solution-phase EPR studies are a safer alternative than solid-state measurements on dispersible powders and have previously been used to identify the presence of U(VI) superoxide when the related potassium uranyl triperoxide phase was dissolved in water^53^. However, dissolution of the **LiU** and **LiNp** phases in water did not result in any signatures associated with the radicals in the sample (SI, Section 4.2, Figs. S7 and S8). Previous work by Nienhuis et al. found that the identity of the alkali base affected the radical production by influencing the local solvation structures^71^. In this case, they noted that in the case of kosmotropic cations (Li^+^), solvent rearrangement has a higher energy barrier, which forms a caged pair that more likely undergoes recombination. However, with chaotropic cations like K^+^, there is a smaller barrier for solvent rearrangement, enabling more radical interactions and formation. This may explain the differences in superoxide radical stabilization between the previous work on K⁺-containing system and the related **LiU** solution^53^.

To enhance our detection capabilities, we turned to the addition of spin traps to evaluate if short-lived radicals can be observed in either the **LiU** or **LiNp** solutions. 5-tert-butoxycarbonyl-5-methyl-1-pyrroline N-oxide (BMPO) was used as a spin trap because it has been previously used for the identification of reactive oxygen species, including superoxide, hydroperoxyl, and hydroxyl radicals^72–76^. BMPO was chosen over the more common 5,5,-dimethyl-1-pyrroline N-oxide (DMPO) spin trap due to a longer half-life of the superoxide adduct^59^. Spin traps, including BMPO, have been developed for biological systems and can be influenced by pH, radical concentrations, and the presence of metal cations. In addition, high levels of radicals and redox-active metals can cause degradation of the BMPO molecule; thus, careful control and modeling of the resulting spectra are required to fully understand the nature of the radicals in the system. These challenges will be further highlighted below.

Dissolution of approximately 5 mg of the **LiU** crystals in 200 µL of water yields a highly alkaline solution, with a pH ranging from 10 to 12 as determined by pH indicator strips. Misak et al. demonstrated that under such conditions, spin trapping experiments using inorganic superoxide (KO_2_) primarily produced hydroxyl radical adducts (BMPO-OH)^72^. This is due to the reaction between superoxide and hydroxyl ions, which generates peroxide and hydroxyl radicals and subsequently interacts with BMPO. Furthermore, **LiU** dissolved with the spin trap under highly alkaline conditions, showing the formation of paramagnetic BMPO adduct degradation product(s) at 60 min (SI, Section 4.3, Fig. S18). However, lowering the pH between 4 and 6 enables the BMPO-OOH spectra to remain relatively stable over the same period. To both enhance superoxide detection and avoid formation of paramagnetic BMPO degradation products, we selected conditions that maintain a final pH between 4 and 6 for all subsequent spin-trap experiments^72^. Additionally, previous work suggested that diethylenetriaminepentaacetic acid (DTPA) should be added to chelate any trace transition metals present in the reagents, which could otherwise interfere with BMPO or its radical adducts^77^. Therefore, DTPA was also added to all of the solutions containing the BMPO spin trap. Control experiments with the uranyl or neptunyl stock and the spin trap showed no detectable EPR signal, indicating An(VI) does not generate paramagnetic products of BMPO under these experimental conditions by itself (SI, 4.3, Fig. S17a–c).

**Fig. 4:**
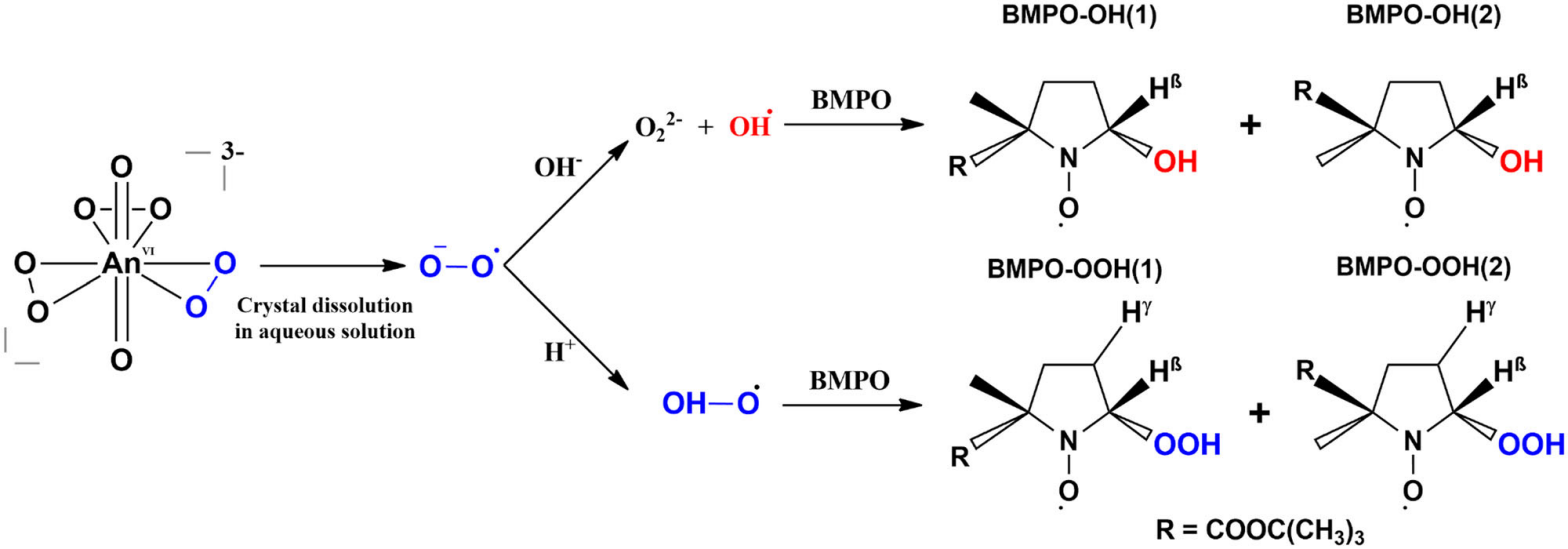
Schematic diagram showing the reactions involved in BMPO adducts formation starting from dissolved crystals containing superoxide complexed to actinyl cation. BMPO-OH/OOH (1) and BMPO-OH/OOH (2) are diastereomers, with (1) adopting the cis configuration and (2) adopting the trans configuration.

Hyperfine splitting parameters of spin traps are sensitive to matrix conditions such as ionic strength and presence of metals^78,79^. To confirm the hyperfine parameters of BMPO-OOH and BMPO-OH adducts under experimental conditions, here matched previously reported values, a control experiment was conducted using KO_2_ at ion types and concentrations comparable to those expected upon dissolution of **LiU** and **LiNp**. The resulting spectra showed the hyperfine parameters were consistent with prior reports (SI, Section 4.3, Figs. S11–S15)^72–74,80,81^. This further supports the use of BMPO as an effective spin trap for superoxide under these experimental conditions. The validated hyperfine parameters (Table 3) were subsequently used to fit the experimental data obtained from spin-trap experiments with **LiU** and **LiNp**. The overall process for the generation of BMPO-OOH and BMPO-OH adducts is also summarized schematically in Fig. 4.

**Table 3:** Average *g*_iso_ and the hyperfine splitting parameters of measured BMPO adducts

Adducts	${g}_{{\mathrm{iso}}}$	${A}_{{{\rm{N}}}}$ (G)	${A}_{{{{\rm{H}}}}^{{{\rm{\beta }}}}}$ (G)	${A}_{{{{\rm{H}}}}^{{{\rm{\gamma }}}}}$ (G)
BMPO-OOH(1)	2.006	13.387	12.000	-
BMPO-OOH(2)	2.006	13.377	9.661	-
BMPO-OH(1)	2.006	13.915	15.365	0.713
BMPO-OH(2)	2.006	14.124	12.674	0.599

**Fig. 5:**
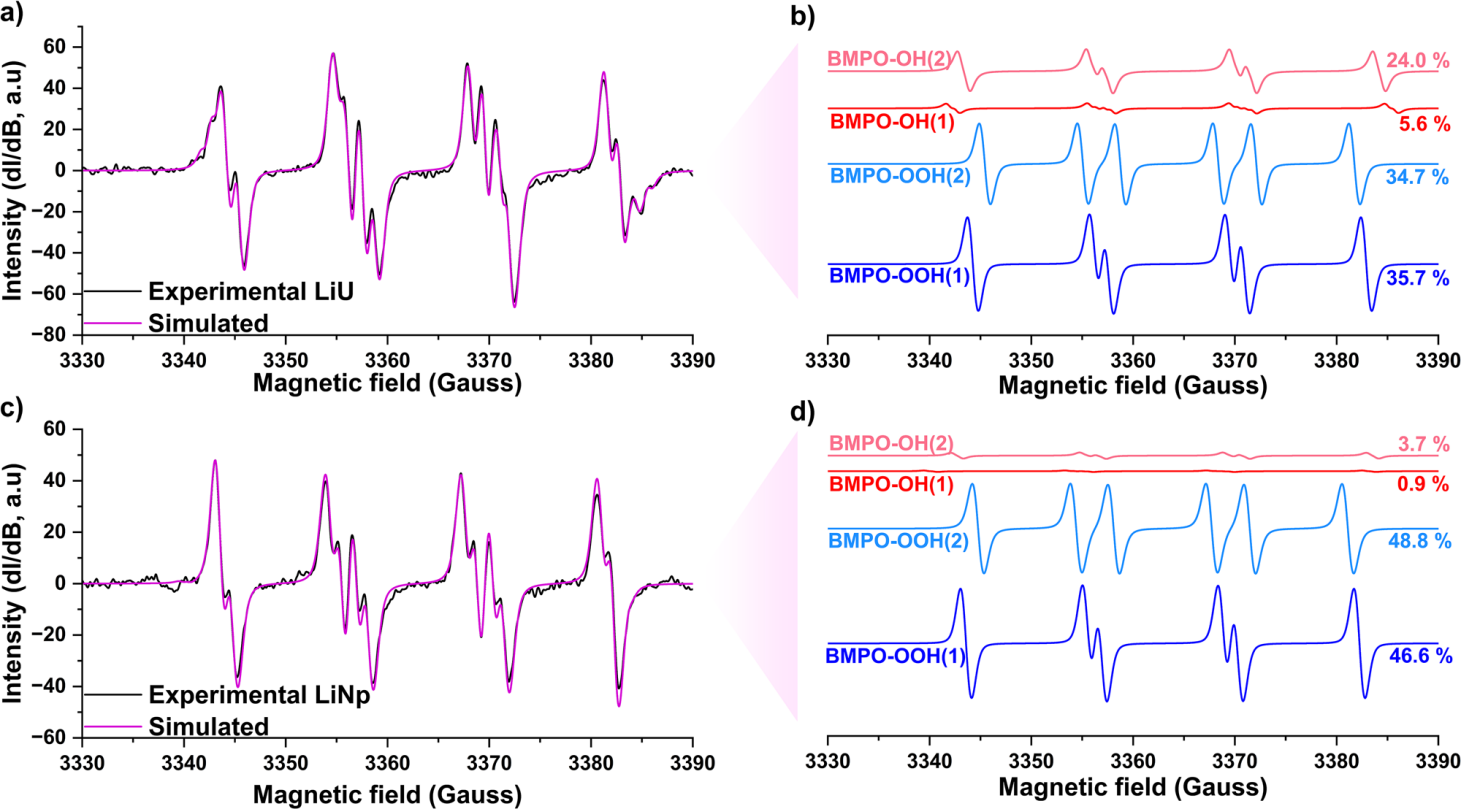
Solution EPR spectra of spin-trap experiments with BMPO on dissolved crystals. **a**, **b** corresponds to experinental(black line) and simulated (purple line) spectra of **LiU** and **LiNp**, respectively. Each BMPO adduct associated with the fits is provided for **c** **LiU** and **d** **LiNp** with the BMPO-OH and BMPO-OOH aducts represented by red and blue lines, respectively. The simulated percentages of each adduct are also provided. Additional simulated fits and experimental EPR spectra are provided in SI, Section 4.3.

The EPR spectra obtained from spin-trap experiments with **LiU** were dominated by the BMPO-OOH adduct (70.4%), with a secondary contribution from the BMPO-OH adduct (28.6%) (Fig. 5b). Similarly, spectra from the **LiNp** experiments showed a BMPO-OOH contribution exceeding 95% (Fig. 5d). To confirm that the observed signals were not the result of the reactivity of peroxide, we evaluated a control solution containing uranyl nitrate and H_2_O_2_ at pH 5 and found that the EPR spectra was silent (SI, 4.3, Fig. S17d). As no superoxide was detected in any control experiments (Section 4.3, Fig. S17), the observed superoxide must arise from the dissolution of the crystalline actinyl triperoxide phase rather than from solution-phase chemistry alone. These results therefore provide direct evidence that superoxide is present within the crystalline materials, supporting the stabilization of superoxide within actinyl triperoxide complexes, forming [AnO_2_(O_2_)_2_(O)_2_^•^]^3−^.

The presence of BMPO-OH adducts is likely due to the generation of hydroxyl radicals via the reaction of superoxide with hydroxide ions. The higher proportion of BMPO-OH observed in the **LiU** experiment may result from a higher pH (~6), while the lower percentage in the **LiNp** experiment corresponds to a lower pH (~4), which limits hydroxyl radical formation. A similar trend was observed in the KO_2_ control experiments, where increasing the pH from 4 to 9 led to a rise in the proportion of BMPO-OH and a corresponding decrease in BMPO-OOH (SI, Section 4.3, Fig. S16).

## Conclusion

This study reports the synthesis and characterization of a lithium neptunyl hydroxo peroxo phase (**LiNp**), which is isostructural to the uranyl analog (**LiU**), enabling direct comparison of U(VI) and Np(VI) peroxide chemistry. Structural analysis revealed subtle differences in bonding; notably, the axial bond in the [AnO_2_(O_2_)_3_]^4−^ unit is 0.016 Å shorter in **LiNp** than in **LiU**, contrary to the typical trend. Raman spectroscopy showed consistent redshifts in actinyl vibrational modes of **LiNp** in comparison to **LiU**, while DFT calculations emphasized the critical role of the second coordination sphere for improving the accuracy of the modeled actinyl vibrational and bonding features. Benchmarking across 22 functionals revealed that current DFT approaches struggle to capture both actinyl and peroxide bonding simultaneously at equal accuracy. Solid-state EPR spectroscopy and DFT calculations confirmed the presence of a coordinated superoxide radical in **LiU**. Although solid-state EPR data for **LiNp** were not collected due to radiological safety constraints, DFT results indicated the thermodynamic feasibility of having superoxide coordinated to neptunyl cation. Solution-phase spin-trapping experiments revealed direct evidence of superoxide in both the **LiU** and **LiNp** systems, supporting the stabilization of superoxide within actinyl triperoxide complexes, forming [AnO_2_(O_2_)_2_(O)_2_^•^]^3−^.

Given the importance of reactive oxygen species within the nuclear fuel cycle, continued efforts are needed to fully understand the chemistry of actinide peroxide species. Future work is needed to fully understand the nature of the actinyl peroxide bonding, and the challenges with the DFT calculations for these systems highlight a frontier in advancing computational modeling of actinyl complexes with stronger axial-ligand interactions. Furthermore, reactivity between free radical species and actinide cations is important given the inherent radioactivity of these elements. Lastly, understanding chemical transformations that may occur due to the presence of reactive oxygen species is necessary to fully understand the behavior of actinides in complex aqueous solutions and predict their fate within the nuclear fuel cycle.

## Methods

Detailed procedures for experimental and computational work can be found in the Supporting Information of this article.

### Synthesis of Li_18_([NpO_2_(O_2_)_3_]_4_[NpO_2_(OH)_4_]) x H_2_O (LiNp)

In a 1-dram vial, 25 μl of Np(VI) stock in 1 M HCl was mixed with LiOH solution (saturated, 100 μl) and 30% H_2_O_2_ solution (100 μl). The resulting solution was mixed until a dark red solution was formed. Next, the dram vial was placed in a 20 mL scintillation vial with ~3 mL methanol, and the scintillation vial was capped. After vapor diffusion overnight, red-brown cubic crystals of **LiNp** appeared at the bottom and walls of the vial. Crystals were isolated by decanting the mother liquor for further analysis.

### Raman spectrocopy

A solid-state Raman spectrum of **LiU** was collected using a SnRI High-Resolution Sierra 2.0 Raman spectrometer outfitted with a 785 nm laser and a 2048 pixel TE-CCD. The laser intensity was set to 100 mW, and spectra were obtained with a 15 s integration period. The **LiNp** spectra were obtained with a Renishaw Raman Microscope outfitted with a 785 nm laser. The laser power was set to 1 mW and the spectra were obtained with 10 s integration time. The final spectrum was obtained after 64 accumulations.

### Solid-state electron paramagnetic resonance (EPR)

**LiU** crystals were ground using a mortar and pestle until a fine powder was obtained. Approximately 50 mg of this powder was loaded into a 4 mm quartz EPR tube, and spectra were collected with a Bruker Magnettech ESR5000 spectrometer. The magnetic field was scanned from 3200 G to 3600 G with a sweep time of 30 s. Modulation was set to 0.4 mT, and Microwave frequency was centered at 9.44 GHz with power set to 50 dB.

### EPR measurements of solutions with spin traps

For **LiU** crystals, approximately 5 mg of powdered sample was dissolved in 100 μL of 0.1 M BMPO solution and 100 μL of 0.1 M DTPA. After homogenizing the mixture for approximately 30 s, the pH of the solution was adjusted to the range of pH 4–6 by adding 15 µL of 2 M HCl. A portion of the resulting solution was transferred into a melting point tube, which was then inserted into a 4 mm quartz EPR tube. For the **LiNp** crystals, the mother liquor was carefully removed from a 1-dram vial containing the crystals, and the residual solid-state materials were left to dry overnight. The following day, 100 μL of 0.1 M BMPO solution, 100 μL of 0.1 M DTPA, and 15 µL of 2 M HCl were added to the vial, and the crystals were dissolved by homogenizing the mixture for approximately 30 s. The resulting solution was transferred to a melting point tube and then placed into a PTFE-FEP EPR tube liner for secondary containment, and this assembly was inserted into a 4 mm quartz EPR tube. EPR measurements were collected immediately using a Bruker Magnettech ESR5000 spectrometer. The magnetic field was scanned from 3300 G to 3450 G with a sweep time of 45 s. Modulation amplitude was set to 0.05 mT, and the microwave frequency was centered at 9.44 GHz with a power setting of 20 dB.

### EPR measurements of solutions with spin traps

All DFT calculations were performed with ORCA 6.0.1^82^ using the B3LYP hybrid functional^83,84^. Relativistic effects were included by Zeroth-Order Regular Relativistic Approximation (ZORA)^85,86^ in combination with ZORA-recontracted^87^ versions of the def2 basis sets^88,89^. All non-actinide atoms were represented by the ZORA-def2-TZVP basis set, while U and Np atoms were represented by the SARC-ZORA-TZVP basis set together with SARC/J coulomb-fitting auxiliary base sets^87–89^. Dispersion correction was applied to all calculations with Grimme’s DFT-D3 dispersion correction with Becke-Johnson damping (D3BJ)^90,91^. Solvation effects were included using the Conductor-like Polarizable Continuum Model^92,93^ with water as the solvent.

## Supplementary information

Revised supporting information document

## Data Availability

Crystallographic data are available under the CCDC number 2433748. Detailed experimental procedures, functional benchmarking, characterization of actinyl peroxide solutions, characterization of LiU and LiNp solid phases, EPR experiments including fitted spectra for spin-trap experiments and control experiments, fitted Raman spectra, and DFT calculation results and optimized coordinates are provided in the Supporting Information of this article.
