# Supplementary material for: Bonding and reactivity of isostructural uranyl and neptunyl peroxide phases
Source: Commun Chem. 2025 Nov 22;8:385. doi: 10.1038/s42004-025-01733-6 (PMC12669678; doi:10.1038/s42004-025-01733-6)
Supplement: Supplementary file 1 — Revised supporting information document [file 42004_2025_1733_MOESM1_ESM.pdf]

Bonding and reactivity of isostructural uranyl and neptunyl peroxide phases

Harindu Rajapaksha[a], Grant C. Benthin[a], Emma L. Markun[a] , Cameron J. Flester[a] , Sara E. Mason*[a,b], Tori Z. Forbes*[a]

[a] *Department of Chemistry, University of Iowa, Iowa City, IA 52242, USA*
[b]*Center for Functional Nanomaterials, Brookhaven National Laboratory, Upton, NY 11973, USA*

*Corresponding Authors: Tori Z. Forbes Email: tori-forbes@uiowa.edu, Sara E. Mason Email: smason@bnl.gov

Supporting Information

Table of Contents

1. Experimental Procedures 7

1.1 Np(VI) Stock Preparation 7

Table S1: ICP-MS data on transition metal ion retention by Dowex 50-X4 cation exchange column. Fraction 1 : 30 mL MQ water, Fraction 2: 30 mL 1 M $HClO_4$, and Fraction 3: 30 mL 1 M $HClO_4$ 9

1.2 Synthesis of Crystaline Materials and Characterization 9

1.3 Raman Spectroscopy 10

1.4 EPR Spectroscopy 10

1.5 DFT Calculations 12

1.6 Benchmarking DFT Functionals 14

Figure S1:Benchmarking geometry optimization with different functionals against experimental bond lengths of $[NpO_2(O_2)_3]^{4-}$ unit. 14

Figure S2:Error in geometry optimized bond length with different functionals against experimental bond lengths of $[NpO_2(O_2)_3]^{4-}$ unit. 15

Figure S3: Benchmarking calculated vibrational wavenumbers with different functionals against experimental values $[NpO_2(O_2)_3]^{4-}$ unit. 16

Figure S4: Error in calculated vibrational wavenumbers with different functionals against experimental values $[NpO_2(O_2)_3]^{4-}$ unit. 17

2. Characterization of actinyl peroxide solution 18

2.1 Optical and Raman spectroscopy Np(VI) peroxide solution 18

3. Characterization of LiU and LiNp solid phase 19

3.1 P-XRD data on LiU 19

Figure S6:P-XRD data of the synthesized **LiU** in comparison to SC-XRD data 19

3.2 SC-XRD data on LiNp... 19

Table S2: Selected crystallographic parameters of the **LiNp** compound............................ 19

4. Radical Chemistry.. 20

4.1 Solution EPR on actinyl triperoxide solution 20

Figure S7: EPR of 25µl of 0.2 M uranyl nitrate stock added with 100 µL of saturated LiOH and 100 µL of 30% H_2O_2 collected at 100 K. The EPR of the capillary + EPR tube was taken as a control (yellow spectra). No new peaks were seen in samples in addition to peaks that are seen in capillary + EPR tube. Room temperature measurements did not display any signal above background for either the capillary + EPR tube or the capillary + EPR tube+ sample. 20

Figure S8: EPR of 25µl of 0.22 M Np(VI) stock added with 100 µL of saturated LiOH and 100 µL of 30% H_2O_2 collected at 100 K. The EPR of the capillary + EPR Liner + EPR tube was taken as a control (yellow spectra). No new peaks were seen in samples in addition to peaks that are seen in capillary + EPR tube. Room temperature measurements did not display any signal above background for either the capillary + EPR tube or the capillary + EPR tube+ sample.. 21

4.2 Experimental and Simulated EPR Spectra of LiU and LiNp crystals dissolved in 0.1 BMPO spin trap solution. 22

Figure S9: Experimental and simulated EPR spectra 5 mg of **LiU** dissolved in 100 µl of 0.1 M BMPO and 100 µl of 0.1 M DTPA at pH 6 22

Figure S10: Experimental and simulated EPR spectra of **LiNp** dissolved in 100 µl of 0.1 M BMPO and 100 µl of 0.1 M DTPA at pH 4.. 23

4.3 BMPO control experiments 24

Figure S11: Experimental and simulated EPR spectra of 1 mg KO_2 dissolved in 100 µl of 0.1 M BMPO and 100 µl of 0.1 M DTPA at pH 4 in the presence of 5 mg of uranyl nitrate and 5 mg of LiCl 24

Figure S12: Experimental and simulated EPR spectra of 1 mg KO_2 dissolved in 100 µl of 0.1 M BMPO and 100 µl of 0.1 M DTPA at pH 5 in the presence of 5 mg of uranyl nitrate and 5 mg of LiCl 25

Figure S13: Experimental and simulated EPR spectra of 1 mg KO_2 dissolved in 100 µl of 0.1 M BMPO and 100 µl of 0.1 M DTPA at pH 7 in the presence of 5 mg of uranyl nitrate and 5 mg of LiCl 26

Figure S14: Experimental and simulated EPR spectra of 1 mg KO_2 dissolved in 100 µl of 0.1 M BMPO and 100 µl of 0.1 M DTPA at pH 8 in the presence of 5 mg of uranyl nitrate and 5 mg of LiCl 27

Figure S15: Experimental and simulated EPR spectra of 1 mg KO_2 dissolved in 100 µl of 0.1 M BMPO and 100 µl of 0.1 M DTPA at pH 9 in the presence of 5 mg of uranyl nitrate and 5 mg of LiCl 28

Figure S16:Percentage of BMPO-OOH and BMPO-OH adducts at pH 4,5,7,8,and 9. EPR spectra was collected with1 mg KO_2 dissolved in 100 µl of 0.1 M BMPO and 100 µl of 0.1 M DTPA in the presence of 5 mg of uranyl. 2M HCl or 1M LiOH was added to adjust the pH........ 29

Figure S17: EPR control experiments with **(a)** 100 µl of 0.1 M BMPO + 100 µl of 0.1 M DTPA at pH 6, **(b)** 100 µl of 0.1 M BMPO + 100 µl of 0.1 M DTPA + 25 µl of 0.2 uranyl nitrate at pH 6, **(c)** 100 µl of 0.1 M BMPO + 100 µl of 0.1 M DTPA + 25 µl of 0.2 Np(VI) stock at pH 6, **(d)** 100 µl of 0.1 M BMPO + 100 µl of 0.1 M DTPA + 25 µl of 0.2 uranyl nitrate + 25 µl of 30% H_2O_2 at pH 6. No EPR signatures were observed for any of the control spectra. 30

Figure S18: EPR measurements of (a) LiU with BMPO at pH 6 and (b) LiU with BMPO at pH 12 over time. At acidic pH, the BMPO-OOH adducts remain stable until 60 mins. In contrast at alkaline pH, the BMPO adducts undergo degradation when reaching 60 mins.. 31

5. Fitted Raman spectra with fitting parameters 32

Figure S19: Fitted Raman Sectra of Neptunyl triperoxide solution made with 500µL sat KOH + 500µL 30% H_2O_2 + 500µL H_2O + 100µL of 0.22 M Np(VI). KOH is used instead of LiOH to obtain higher signal without causing solid-state precipitation. The spectral fitting parameters for the spectra are reduced R^2 = 0.9984 and reduced χ^2 = 3.16 x 10^{-4}. ... 32

Figure S20: Solid-state Raman spectra of pristine **LiU**. The spectrum was fit with a Pseudo-Voigt function and fitting parameters for the spectra are reduced R^2 = 0.9984 and reduced χ^2 = 5.73 x 10^{-5}. 33

Figure S21: Solid-state Raman spectra of pristine **LiNp**. The spectrum was fit with a Pseudo-Voigt function and fitting parameters for the spectra are reduced R^2 = 0.9981 and reduced χ^2 = 7.68 x 10^{-5}. 34

Figure S22: Solution Raman of H_2O_2 in 2 M KOH solution. 35

6. DFT Calculations Results 36

6.1 Models I-VI Used in DFT Calculations 36

Figure S23: Models I-IV were used in DFT calculations of the actinyl triperoxide units and models V-VI were used in calculation of actinyl tetra hydroxide unit. The maroon, red, purple, and white spheres represent U or Np, O, Li, and H atoms, respectively. 36

6.2 DFT Calculated Bond Lengths, Vibration Frequencies and Energetics 37

Table S3: Summary of DFT calculated bond lengths and vibration frequencies for $[UO_2(O_2)_3]^{4-}$ and $[UO_2(O_2)_2O_2^{•}]^{3-}$ species with different second-sphere coordination environments (Model I-IV).. 37

Table S4: Summary of DFT calculated bond lengths and vibration frequencies for $[NpO_2(O_2)_3]^{4-}$ and $[NpO_2(O_2)_2O_2^{•}]^{3-}$ species with different second-sphere coordination environments (Model I-IV).. 38

Table S5: Summary of DFT calculated bond lengths and vibration frequencies for $[UO_2(OH)_4]^{2-}$ and $[UO_2(OH)_4]^{-}$ species with different second-sphere coordination environments (Model V and VI).. 39

Table S6: Summary of DFT calculated bond lengths and vibration frequencies for $[NpO_2(OH)_4]^{2}$ and $[NpO_2(OH)_4]^{-}$ species with different second-sphere coordination environments (Model V and VI).. 39

Table S7: Energetics comparing the relative stabilities of $[NpO_2(O_2)_2(O)_2^{•}]^{3-}$ and $[UO_2(O_2)_2(O)_2^{•}]^{3-}$ species within the crystalline matrix.. 39

6.3 Selected Vibration Modes 40

Figure S24: Selected vibrational modes of actinyl triperoxide and actinyl tetrahydroxide moieties. The maroon, red, and white spheres represent actinyl (U or Np), O, and H atoms respectively. The blue arrow represents the displacement vectors of atoms with different vibrational modes 40

6.4 Molecular Orbital and Spin Density Analysis 41

Figure S25: HOMO-3 to LUMO+3 molecular orbitals of **(a)** $[UO_2(O_2)_3]^{4-}$ and **(b)** $[UO_2(OH)_4]^{2-}$ units from the DFT calculations. The blue, red, and white spheres represent U, O, and H atoms, respectively. Surfaces were generated with iso value of 0.030............ 41

Figure S26: Spin density of **(a)** $[UO_2(O_2)(O_2)^{•}]^{3-}$, **(b)** $[NpO_2(O_2)_2(O_2)^{•}]^{3-}$, **(c)** $[UO_2(OH)_4]^{-}$, and **(d)** $[NpO_2(OH)_4]^{-}$ moieties determined from the DFT calculations. The blue, maroon, red, and white spheres represent U, Np, O, and H atoms respectively. The surfaces were generated with iso value of 0.015. 42

Figure S27: Spin density of **(a)** $[UO_2(O_2)(O_2)^{•}]^{3-}$, **(b)** $[NpO_2(O_2)_2(O_2)^{•}]^{3-}$, **(c)** $[UO_2(OH)_4]^{-}$, and **(d)** $[NpO_2(OH)_4]^{-}$ complexes with inclusion of the secondary coordination sphere. The blue, maroon, red, purple, and white spheres represent U, Np, O, Li, and H atoms, respectively. The surfaces were generated with iso value of 0.015. 43

6.5 QTAIM Analysis 44

Table S8: QTAIM on the $[AnO2(O2)3]4-$ unit. The U, Np, and Np-U column represent results for $[UO2(O2)3]4-$, $[NpO2(O2)3]4-$ and the difference between the two respectively. Here topological Parameters of Electron Density are Electron Density ($\boldsymbol{\rho}$), Its

Laplacian ($\nabla 2\rho$), Local Kinetic Energy Density (G), Potential Energy Density (V) and Electronic Energy Density (H). All data are given in arbitrary units 44

Table S9: QTAIM on the $[AnO2(OH)4]2-$ unit. The U, Np, and Np-U column represent results for $[UO2(OH)4]2-$, $[NpO2(OH)4]2-$ and the difference between the two respectively. Here topological Parameters of Electron Density are Electron Density (ρ), Its Laplacian ($\nabla 2\rho$), Local Kinetic Energy Density (G), Potential Energy Density (V) and Electronic Energy Density (H). All data are given in arbitrary units 44

6.6 DFT optimized coordinates.. 45

Table S10: DFT optimized coordinates of Model I - $[UO2(O2)3]4-$............................... 45

Table S11: DFT optimized coordinates of Model I - $[UO2(O2)2(O2)\bullet]3-$..................... 45

Table S12: DFT optimized coordinates of Model I - $[NpO2(O2)3]4-$ 45

Table S13: DFT optimized coordinates of Model I - $[NpO2(O2)2(O2)\bullet]3-$.................. 45

Table S14: DFT optimized coordinates of Model II - $[UO2(O2)3]4-$ 46

Table S15: DFT optimized coordinates of Model II - $[UO2(O2)2(O2)\bullet]3-$................... 47

Table S16: DFT optimized coordinates of Model II - $[NpO2(O2)3]4-$........................... 48

Table S17: DFT optimized coordinates of Model II - $[NpO2(O2)2(O2)\bullet]3-$ 49

Table S18: DFT optimized coordinates of Model III - $[UO2(O2)3]4-$............................ 50

Table S19: DFT optimized coordinates of Model III - $[UO2(O2)2(O2)\bullet]3-$ 52

Table S20: DFT optimized coordinates of Model III - $[NpO2(O2)3]4-$.......................... 53

Table S21: DFT optimized coordinates of Model III - $[NpO2(O2)2(O2)\bullet]3-$................ 54

Table S22: DFT optimized coordinates of Model IV - $[UO2(O2)3]4-$............................ 55

Table S23: DFT optimized coordinates of Model IV - $[UO2(O2)2(O2)\bullet]3-$ 56

Table S24: DFT optimized coordinates of Model IV - $[NpO2(O2)3]4-$.......................... 58

Table S25: DFT optimized coordinates of Model IV - $[NpO2(O2)2(O2)\bullet]3-$................ 59

Table S26: DFT optimized coordinates of Model V - $[UO2OH4]2-$ 60

Table S27: DFT optimized coordinates of Model V - $[UO2OH4]-$ 61

Table S28: DFT optimized coordinates of Model V - $[NpO2OH4]2-$............................... 61

Table S29: DFT optimized coordinates of Model V - $[NpO2OH4]-$................................ 61

Table S30: DFT optimized coordinates of Model VI - $[UO2OH4]2-$ 62

Table S31: DFT optimized coordinates of Model VI - $[UO2OH4]-$................................. 64

Table S32: DFT optimized coordinates of Model VI - $[NpO2OH4]2-$ 66

Table S33: DFT optimized coordinates of Model VI - $[NpO2OH4]-$ 69
7.0 References 72

1. Experimental Procedures

1.1 Np(VI) Stock Preparation

Caution: Uranyl nitrate hexahydrate contains U-238 (depleted uranium), standard precautions and licensing for handling radioactive substances should be followed. Np-237 is a highly radioactive alpha emitter, and its daughter Pa-233 is a highly radioactive beta emitter. Research with this isotope is restricted to specialized laboratories and must be handled under appropriate regulatory controls.

Preparation of Np(VI) stock in 1M HCl: Np-237 was recovered from a waste stream and processed to prepare a ~0.22 M Np(VI) stock solution in 1 M HCl. The general reprocessing procedure was as follows: solutions from multiple waste streams were combined and evaporated to dryness, yielding crystalline salt residues. These residues were washed and leached with concentrated HCl to selectively dissolve Np-237 while minimizing the dissolution of excess salts, which exhibit limited solubility in concentrated HCl. This step was aimed at reducing salt content in the final solution. Matrix exchange was performed by evaporating the HCl solution to dryness and redissolving the residue in 4 M HNO_3. Ozonation of the resulting solution was carried out for 30 minutes using an ozone flow rate of ~0.5 $L \cdot min^{-1}$ with an O_3 output of ~2.7 $g \cdot h^{-1}$ to ensure complete oxidation of neptunium to Np(VI) and to degrade any residual organic compounds from prior experiments. Liquid–liquid extraction was then conducted using acid washed, 30% tributyl phosphate (TBP) in dodecane. Np(VI) was back-extracted into 0.1 M HCl after reduction to Np(V) using $NaNO_2$. Precipitation of neptunium hydroxide was achieved by the careful addition of a minimal quantity saturated NaOH solution. The precipitate was washed three times with water and redissolved in 1 M $HClO_4$. To remove transition metal impurities, the solution was loaded onto a Dowex 50-X4 cation exchange column. Np(V) was retained on the column and washed with

approximately two column volumes of deionized water. It was then eluted with 1 M $HClO_4$, and the Np(V)-containing fractions were combined. A final matrix exchange was performed by evaporating the solution and redissolving the residue in 1 M HCl. To reoxidize Np(V) to Np(VI), a final ozonation step was performed for 15 minutes. The concentration of Np(VI) in the resulting stock solution was determined by Liquid Scintillation Counting (LSC).

To analyze the effectiveness of the Dowex 50-X4 cation exchange column in removing first row transition metal ions (a common contaminant that might interfere with EPR measurements), the following control experiment was done. A 10.0942 g aliquot of 10 ppm Inductively Coupled Plasma (ICP) standard for metals (Inorganic Venture -ICMS-71A) Cr, Mn, Fe, Co, Ni and Cu in 2% HNO_3 was evaporated to dryness and redissolved in 11.2170 g of 1 M $HClO_4$. To simulate the NpO_2^+ and alkaline metal cations competing for cation exchange sites, 800 mg of NaCl was dissolved in this solution. An identical column to the one utilized for Np samples was prepared and 2.4046 g of the ICP-standard solution was loaded into the column. The column was rinsed with 2 column volumes of Millipore water (18.2 Ω) (~60 mL) and the collected eluent was labeled Fraction 1. To simulate the elution of the NpO_2^+, 2 x 30 mL of 1 M $HClO_4$ was passed through the column and fractions were collected as Fraction 2 and Fraction 3, respectively. A matrix exchange was done by evaporating under heat and the final solution was made by dissolving the residue in 2% HNO_3. This solution was analyzed with a 7800 Agilent Inductively Coupled Plasma Mass Spectrometer (ICP-MS) with a four 4-point calibration curves were prepared at 10 ppb, 100 ppb, 1000 ppb, and 2000 ppb using Inorganic Venture -ICMS-71A standard. The results show that >85% of the metal ions are retained in the column during the total 60 mL of 1 M $HClO_4$ elution (Table S1).

Table S1: ICP-MS data on transition metal ion retention by Dowex 50-X4 cation exchange column. Fraction 1 : 30 mL MQ water, Fraction 2: 30 mL 1 M $HClO_4$, and Fraction 3: 30 mL 1 M $HClO_4$

	Fraction	Cr	Mn	Fe	Co	Ni	Cu
Concentration (ppb)	Fraction 1	30.352 ±0.9	10.271 ±2.8	73.343 ±0.3	7.467 ±2.2	12.902 ±1.2	14.633 ±1.5
	Fraction 2	63.953 ±1.9	14.382 ±2.4	119.962 ±0.5	0.800 ±2.9	41.069 ±2.1	25.985 ±0.7
	Fraction 3	63.794 ±0.8	17.853 ±0.5	139.592 ±0.6	0.979 ±1.7	33.897 ±1.3	43.296 ±1.1
Total amount (µg)	Fraction 1	308.671 ±6.1	104.453 ±28.5	745.876 ±3.1	75.937 ±22.4	131.209 ±12.2	148.813 ±14.2
	Fraction 2	640.489 ±9.0	144.036 ±24.0	1201.419 ±5.0	8.012 ±29.0	411.306 ±21.0	260.240 ±7.0
	Fraction 3	637.589 ± 5.0	178.432 ±5.0	1401.958 ± 6.0	9.785 ±17.0	338.784 ±13.0	432.722 ±11.0
% of total loaded amount eluted	Fraction 1	1.43 ±0.03 %	0.48 ±0.13 %	3.45 ±0.01 %	0.35 ±0.10 %	0.61 ±0.06 %	0.69 ±0.07 %
	Fraction 2	2.96 ±0.04 %	0.67 ±0.11 %	5.55 ±0.02 %	0.04 ±0.13 %	1.90 ±0.97 %	1.20 ±0.03 %
	Fraction 3	2.95 ±0.02 %	0.82 ±0.02 %	6.48 ±0.03 %	0.05 ±0.80 %	1.57 ±0.60 %	2.00 ±0.05 %

1.2 Synthesis of Crystaline Materials and Characterization

*Synthesis of $Li_{18}([UO_2(O_2)_3]_4[UO_2(OH)_4]).xH_2O$ (**LiU**):* Synthesis of **LiU** has been previously reported[1]. In a 20 mL scintillation vial, uranyl nitrate solution (0.2 M, 2.00 mL) was mixed with LiOH solution (saturated, 1.00 mL) and 30% H_2O_2 solution (1 mL). The resulting mixture was sonicated until the solution turned orange and no visual signs of suspended solids remained. Next, the scintillation vial was placed in a 100 mL beaker with 20 mL methanol and was covered with parafilm. After a week of vapor diffusion, crystals started to appear with a yield of approximately 80-85%. Crystals were isolated by decanting the mother liquor, and the purity of the product was *confirmed* with powder X-ray diffraction.

*Synthesis of $Li_{18}([NpO_2(O_2)_3]_4[NpO_2(OH)_4]).xH_2O$ (**LiNp**):* In a 1 dram vial, 25 µl of Np(VI) stock in 1 M HCl was mixed with LiOH solution (saturated, 100 µl) and 30% H_2O_2 solution (100 µl). The resulting solution was mixed until a dark red solution was formed. Next, the dram vial was placed in a 20 mL scintillation vial with ~3 mL methanol and the scintillation vial was capped. After vapor diffusion overnight, red-brown cubic crystals of **LiNp** appeared at the bottom and walls of the vial. Crystals were isolated by decanting the mother liquor for further analysis.

Single Crystal X-ray Diffraction (SC-XRD): Single crystals of **LiNp** were mounted on a MiTeGen MicroMount and reflections were collected using 0.5° ω scans on Brucker® D8 Quest single X-ray diffractometer ($\lambda_{MoK\alpha}$= 0.71073 Å) with PHOTON detector and Oxford® cryo-system. Absorption corrections were performed using the SADABS program. APEX3 intrinsic phasing 1 was used for the solving of structures and the structure refinements were done SHELXL with OLEX2 software suite.[2]

1.3 Raman Spectroscopy

A solid-state Raman spectrum of **LiU** was collected using SnRI High-Resolution Sierra 2.0 Raman spectrometer outfitted with a 785 nm laser and a 2048 pixel TE-CCD. The laser intensity was set to 100 mW and spectra were obtained with a 15s integration period. The **LiNp** spectra was obtained with a Renishaw Raman Microscope outfitted with a 785 nm laser. The laser power was set 1 mW and the spectra was obtained with 10s integration time. The final spectrum was obtained after 64 accumulations. Spectral fitting for both compounds was performed using the Origin Pro software.

1.4 EPR Spectroscopy

Solid-State Electron Paramagnetic Resonance (EPR): **LiU** crystals were ground using a mortar and pestle until a fine powder was obtained. Approximately 50 mg of this powder was loaded into

a 4 mm quartz EPR tube and spectra were collected with a Bruker Magnettech ESR5000 spectrometer. Magnetic field was scanned from 3200 G to 3600 G with a sweep time of 30 s. Modulation was set to 0.4 mT and Microwave frequency was centered at 9.44 GHz with power set to 50 dB. Thirty accumulations were collected and averaged to produce the final spectra. Background subtraction was done with Origin Pro software.

Note: The conditions used in the spin-trap experiments are expected to alter the LiU and LiNp complexes during dissolution. The purpose of the spin trap is to capture transient radicals by forming stable adducts. Alteration of the LiU and LiNp compounds during dissolution is acceptable, as control experiments containing only uranyl and peroxide at the same pH did not yield any superoxide adducts. Therefore, any observed superoxide signal can be attributed to the release of superoxide during the dissolution of the LiU and LiNp complexes.

Solution EPR measurements with spin-traps: For **LiU** crystals, approximately 5 mg of powdered sample was dissolved in 100 µL of 0.1 M BMPO solution and 100 µL of 0.1 M DTPA. After homogenizing the mixture for approximately 30 seconds, pH of the solution was adjusted to the range of pH 4-6 by adding 15 µL of 2M HCl. A portion of the resulting solution was transferred into a melting point tube, which was then inserted into a 4 mm quartz EPR tube. EPR measurements were collected immediately using a Bruker Magnettech ESR5000 spectrometer. The magnetic field was scanned from 3300 G to 3450 G with a sweep time of 45s. Modulation amplitude was set to 0.05 mT, and the microwave frequency was centered at 9.44 GHz with a power setting of 20 dB.

For the **LiNp** crystals, in-lab sample preparation near the instrument was not feasible due to radiological safety concerns. Therefore, the mother liquor was carefully removed from a 1-dram vial containing the crystals and and the residual solid-state materials were left to dry overnight.

The following day, 100 μL of 0.1 M BMPO solution, 100 μL of 0.1 M DTPA, and 15 μL of 2M HCl was added to the vial, and the crystals were dissolved by homogenizing the mixture for approximately 30 seconds. The resulting solution was transferred to a melting point tube and then placed into a PTFE-FEP EPR tube liner for secondary containment. This assembly was inserted into a 4 mm quartz EPR tube, and the sample was immediately frozen using liquid nitrogen. This procedure added approximately 7-10 minutes to the protocol described for the **LiU** sample. The sample was kept frozen until EPR measurements were performed using the parameters outlined in **LiU** procedure.

1.5 DFT Calculations

DFT calculations: All DFT calculations were performed with ORCA 6.0.1[3] using the B3LYP hybrid functional.[4,5] Twenty-two functionals were benchmarked and B3LYP was chosen as it in higher accuracy in replicating experimental bond lengths (Figure S1 and Figure S2) and Raman frequencies (Figure S3 and Figure S4) of both neptunyl cation and peroxide ligands. For these calculations, the relativisticeffects are included by Zeroth-Order Regular Relativistic Approximation (ZORA)[6,7] in combination with ZORA-recontracted[8] versions of the def2 basis sets.[9,10] All non-actinide atoms are represented by the ZORA-def2-TZVP basis set while U and Np atoms are represented by the SARC-ZORA-TZVP basis set together with SARC/J coulomb-fitting auxiliary base sets.[8-10] All geometry optimizations were performed with tight convergence criteria with sets total Energy change, total RMS gradient and total MAX gradient to $1e^{-6}$ au, $3e^{-5}$ au, and $1e^{-4}$ respectively. All SCF calculations were also performed with tight convergence criteria which set energy change to $1e^{-8}$ au. Dispersion correction was applied to all calculation with Grimme's DFT-D3 dispersion correction with Becke-Johnson damping (D3BJ)[11,12] When performing geometry optimizations with the secondary coordination sphere, the position of water oxygen and

Li^+ atoms were fixed, and the optimization was only performed on the actinyl unit and the hydrogens. All calculations, including the ones with the added secondary coordination sphere were performed using Conductor-like Polarizable Continuum Model (CPCM)[13,14] with water as the solvent. Visualization of spin densities was done using the Chemcraft program.[15] QTAIM analysis was done using Multiwfn.[16]

1.6 Benchmarking DFT Functionals

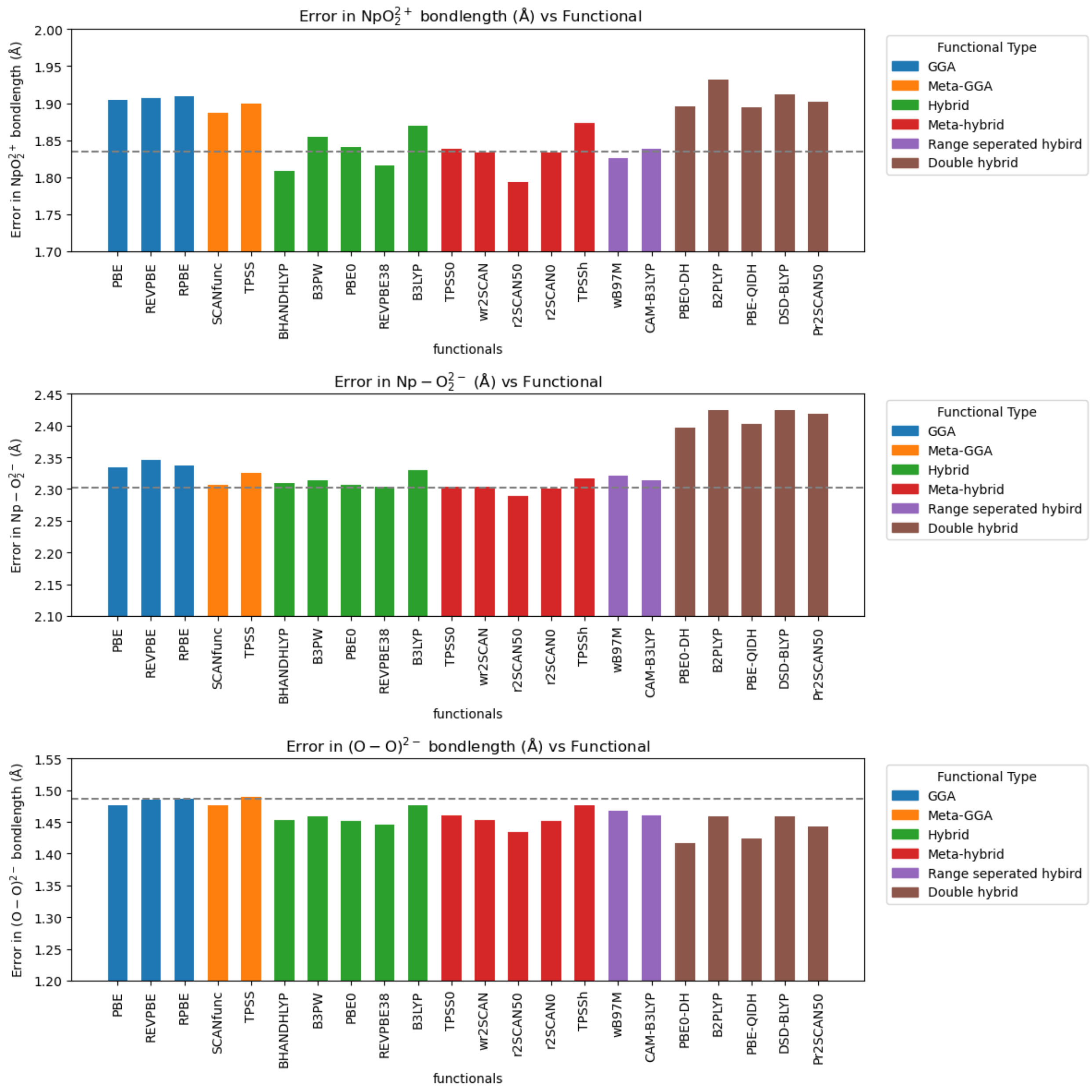

Figure S1:Benchmarking geometry optimization with different functionals against experimental bond lengths of $[NpO_2(O_2)_3]^{4-}$ unit.

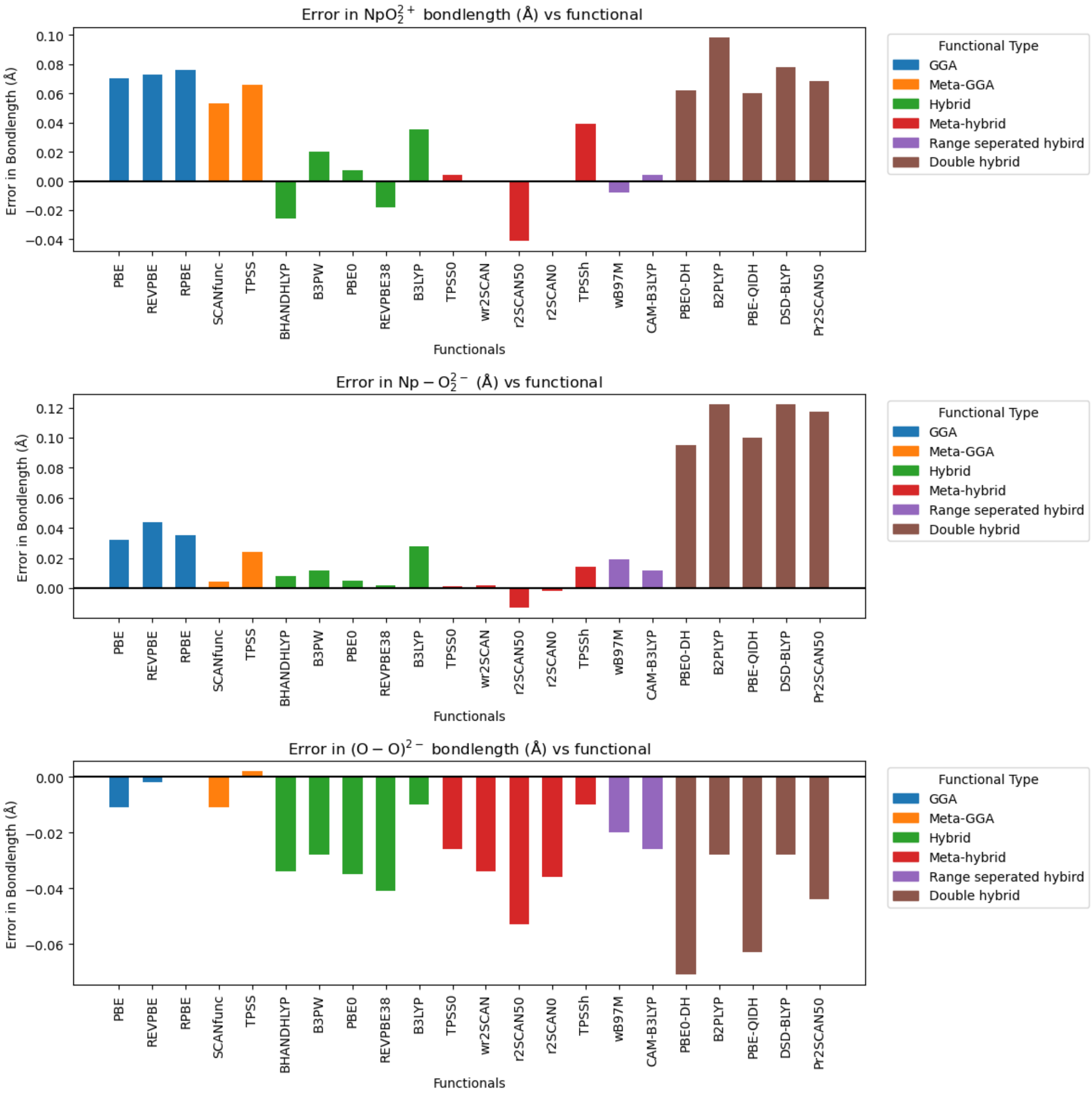

Figure S2:Error in geometry optimized bond length with different functionals against experimental bond lengths of $[NpO_2(O_2)_3]^{4-}$ unit.

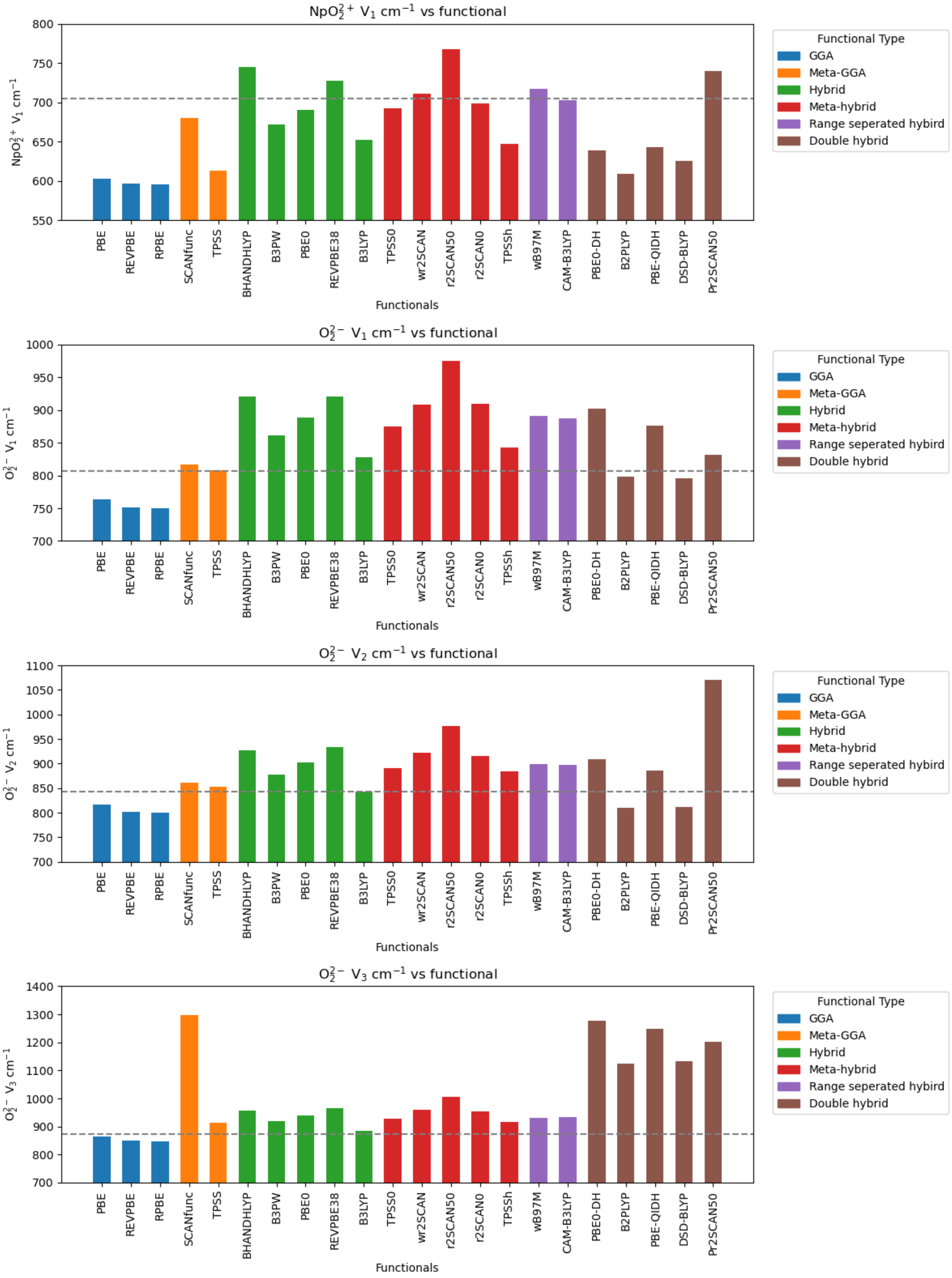

Figure S3: Benchmarking calculated vibrational wavenumbers with different functionals against experimental values $[NpO_2(O_2)_3]^{4-}$ unit.

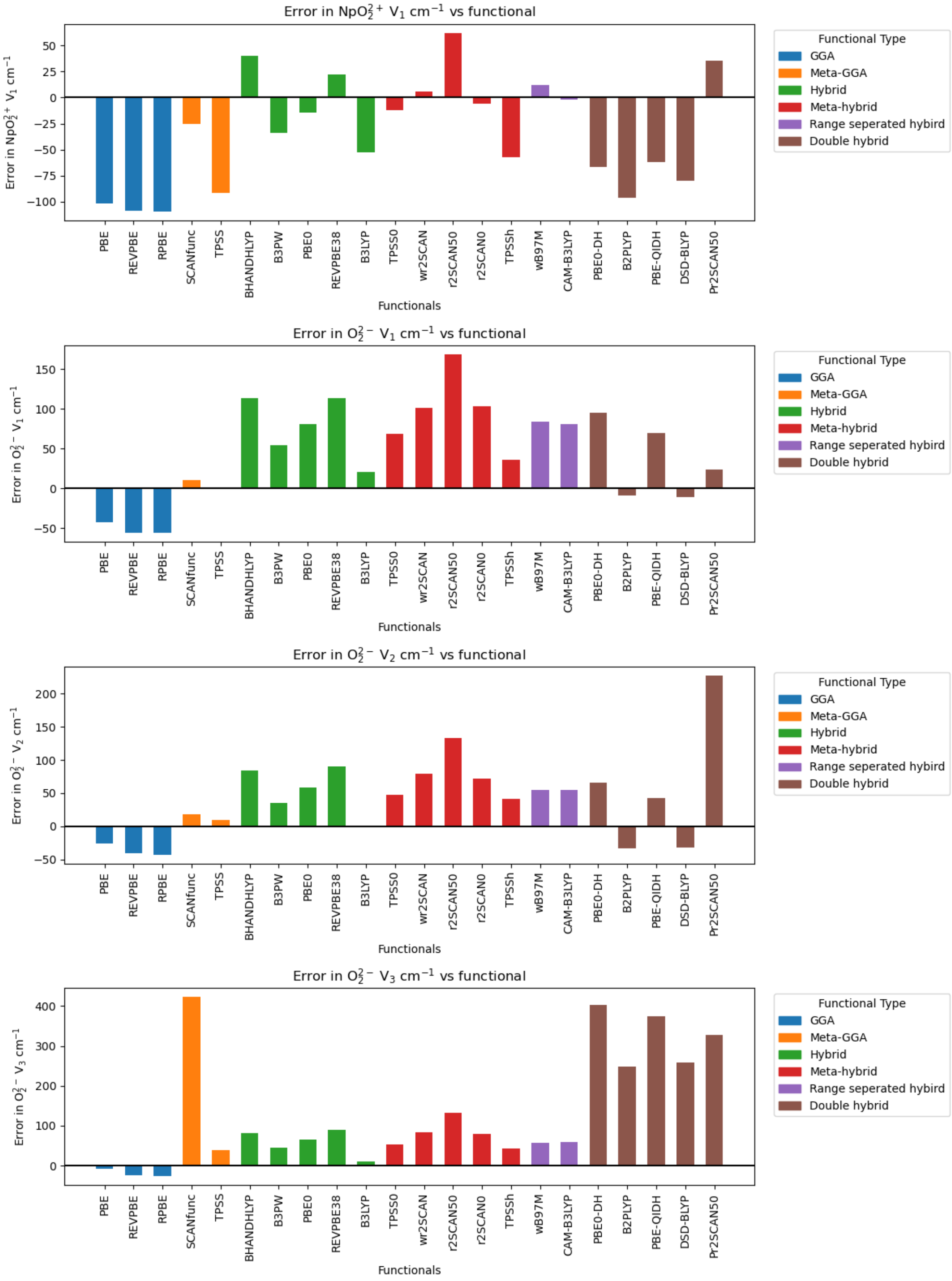

Figure S4: Error in calculated vibrational wavenumbers with different functionals against experimental values $[NpO_2(O_2)_3]^{4-}$ unit.

2. Characterization of actinyl peroxide solution

2.1 Optical and Raman spectroscopy Np(VI) peroxide solution

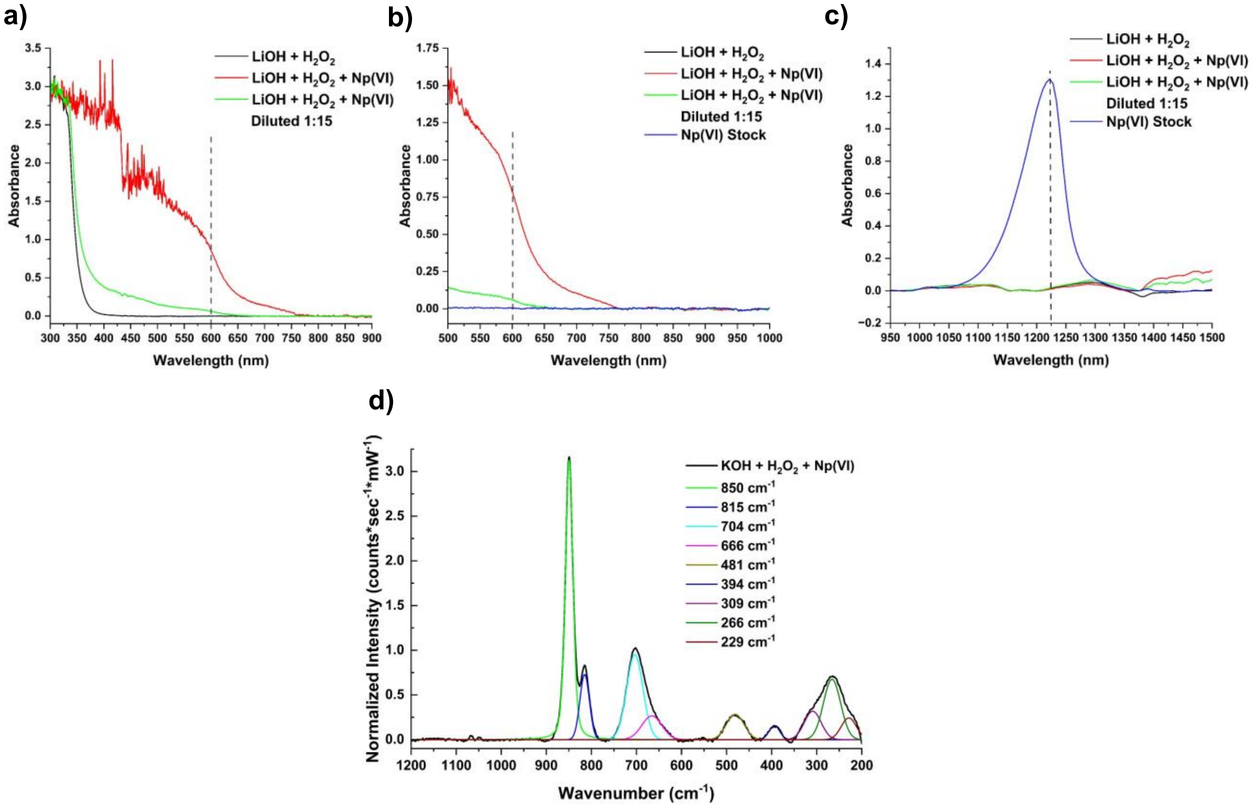

Figure S5: The **(a)** UV, **(b)** Vis, **(c)** NIR, and **(d)** Raman spectroscopy was collected for the neptunyl peroxide solution. Optical spectroscopies were taken from a control solution (black line - 500µL sat LiOH + 500µL 30% H_2O_2 + 500µL H_2O) and three Np(VI) solutions. Red line : 500µL sat LiOH + 500µL 30% H_2O_2 + 500µL H_2O + 25µL of 0.22 M Np(VI)). Green line: 15-fold diluted sample. Blue line: Np(VI) stock solution in 1M HCl. Solution Raman was taken from 500µL sat KOH + 500µL 30% H_2O_2 + 500µL H_2O + 100µL of 0.22 M Np(VI). KOH is used instead of LiOH to obtain higher signal while avoiding rapid precipitation of the sample.

3. Characterization of LiU and LiNp solid phase

3.1 P-XRD data on LiU

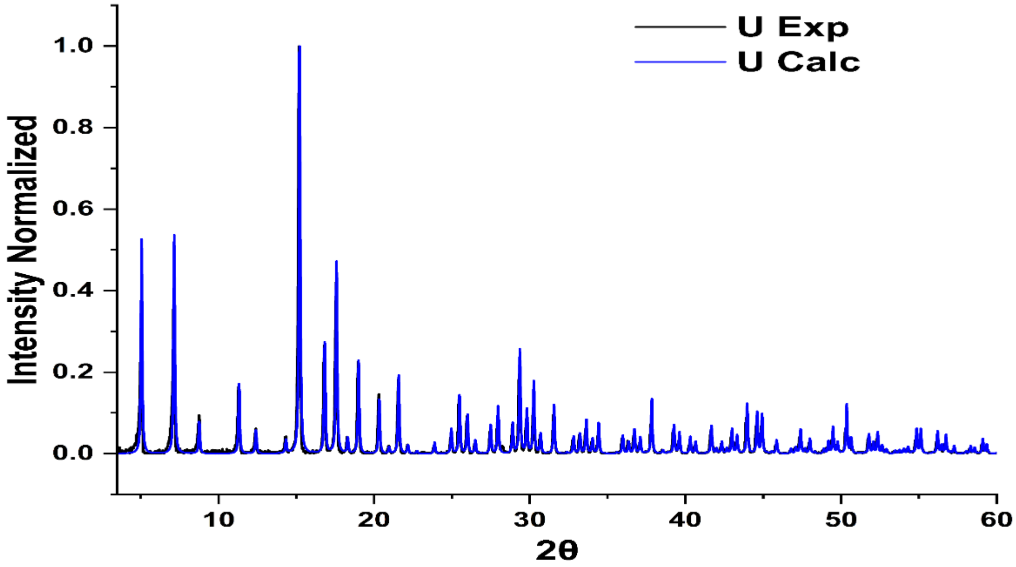

Figure S6:P-XRD data of the synthesized **LiU** in comparison to SC-XRD data

3.2 SC-XRD data on LiNp

Table S2: Selected crystallographic parameters of the **LiNp** compound.

Empirical Formula	$Li_{18}Np_5O_{77.5}$	μ (mm^{-1})	7.396
Crystal color, habit	Redish brown, cubic	F(000)	3417.0
Formula weight	2549.76	Θ range (°)	4.042 to 56.764
Crystal system	cubic	Limiting indices	$-23 \leq h \leq 23$,
a (Å)	17.4525(3)		$-23 \leq k \leq 23$,
b (Å)	17.4525(3)		$-23 \leq l \leq 23$
c (Å)	17.4525(3)	Ref. collected/unique	263897
α (°)	90°	R_{int}	0.0477
β (°)	90°	Data/restraints/parameters	1386/54/114
γ (°)	90°	GOF on F^2	1.122
Volume ($Å^3$)	5315.9(3)	Final R indices ([I>2σ(I)]) R_1	0.0274
Temperature (K)	100	Final R indices ([I>2σ(I)]) wR_2	0.0779
Density, ρ (g/cm^3)	2.389	R indices (all data) R_1	0.0289
Space group	*Pm*-3*m*	R indices (all data) wR_2	0.0792
Z	3	Largest diff. peak/hole (e $Å^{-3}$)	1.93 and -1.37
Radiation type	MoKα (λ = 0.71073)	CCDC deposition number	2433748

4. Radical Chemistry

4.1 Solution EPR on actinyl triperoxide solution

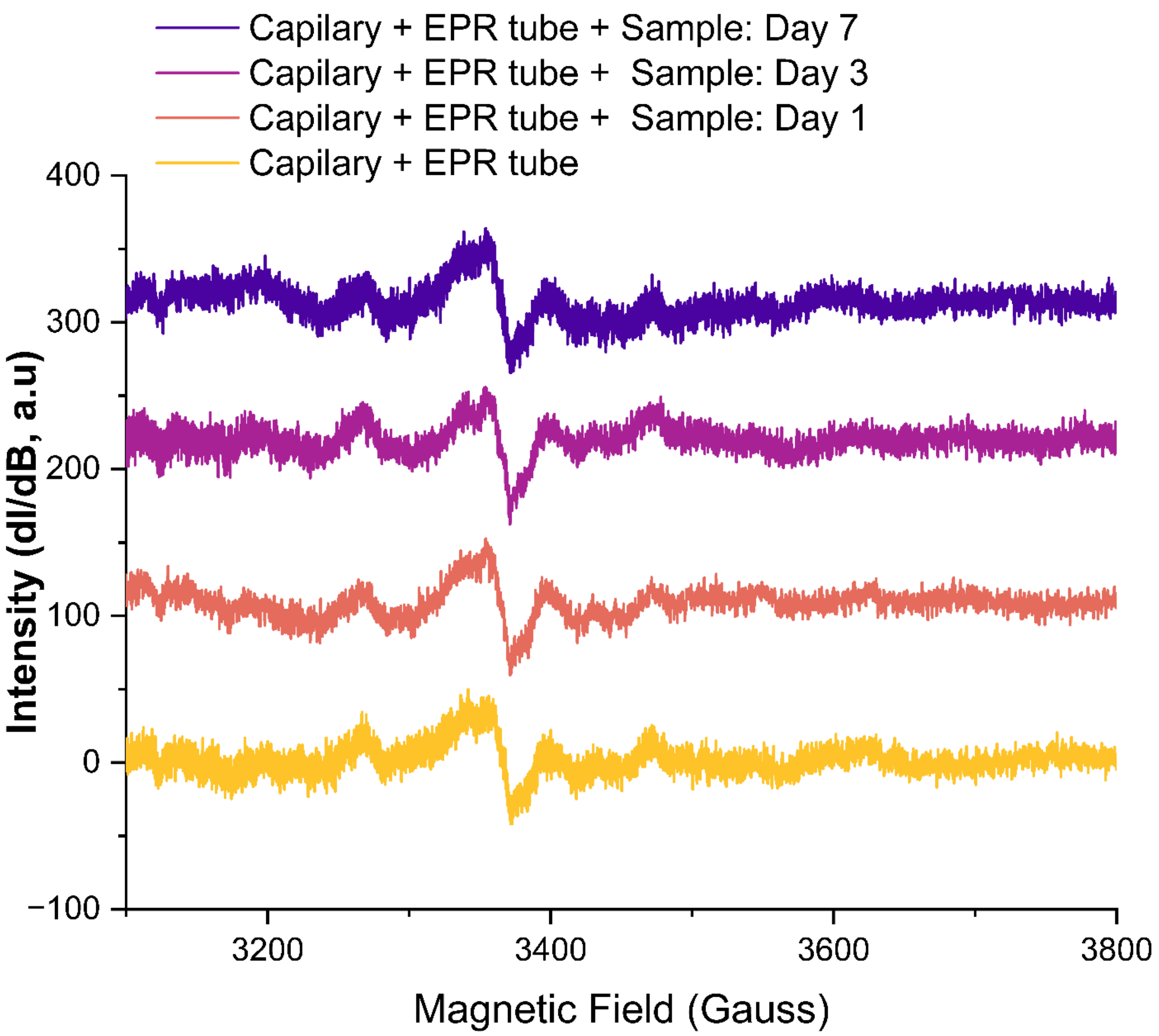

Figure S7: EPR of 25µl of 0.2 M uranyl nitrate stock added with 100 µL of saturated LiOH and 100 µL of 30% H_2O_2 collected at 100 K. The EPR of the capillary + EPR tube was taken as a control (yellow spectra). No new peaks were seen in samples in addition to peaks that are seen in capillary + EPR tube. Room temperature measurements did not display any signal above background for either the capillary + EPR tube or the capillary + EPR tube+ sample.

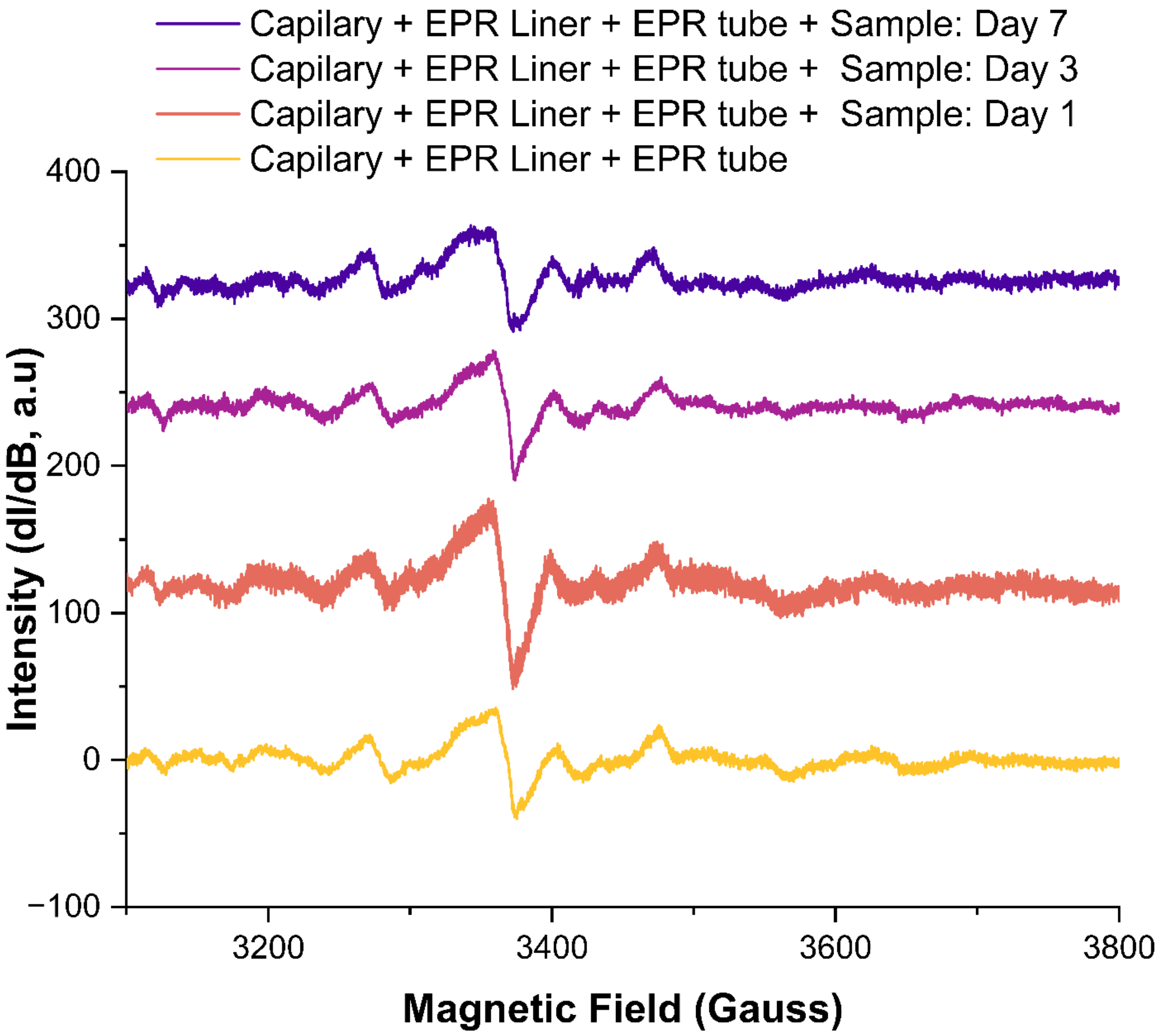

Figure S8: EPR of 25µl of 0.22 M Np(VI) stock added with 100 µL of saturated LiOH and 100 µL of 30% H_2O_2 collected at 100 K. The EPR of the capillary + EPR Liner + EPR tube was taken as a control (yellow spectra). No new peaks were seen in samples in addition to peaks that are seen in capillary + EPR tube. Room temperature measurements did not display any signal above background for either the capillary + EPR tube or the capillary + EPR tube+ sample.

4.2 Experimental and Simulated EPR Spectra of LiU and LiNp crystals dissolved in 0.1 BMPO spin trap solution.

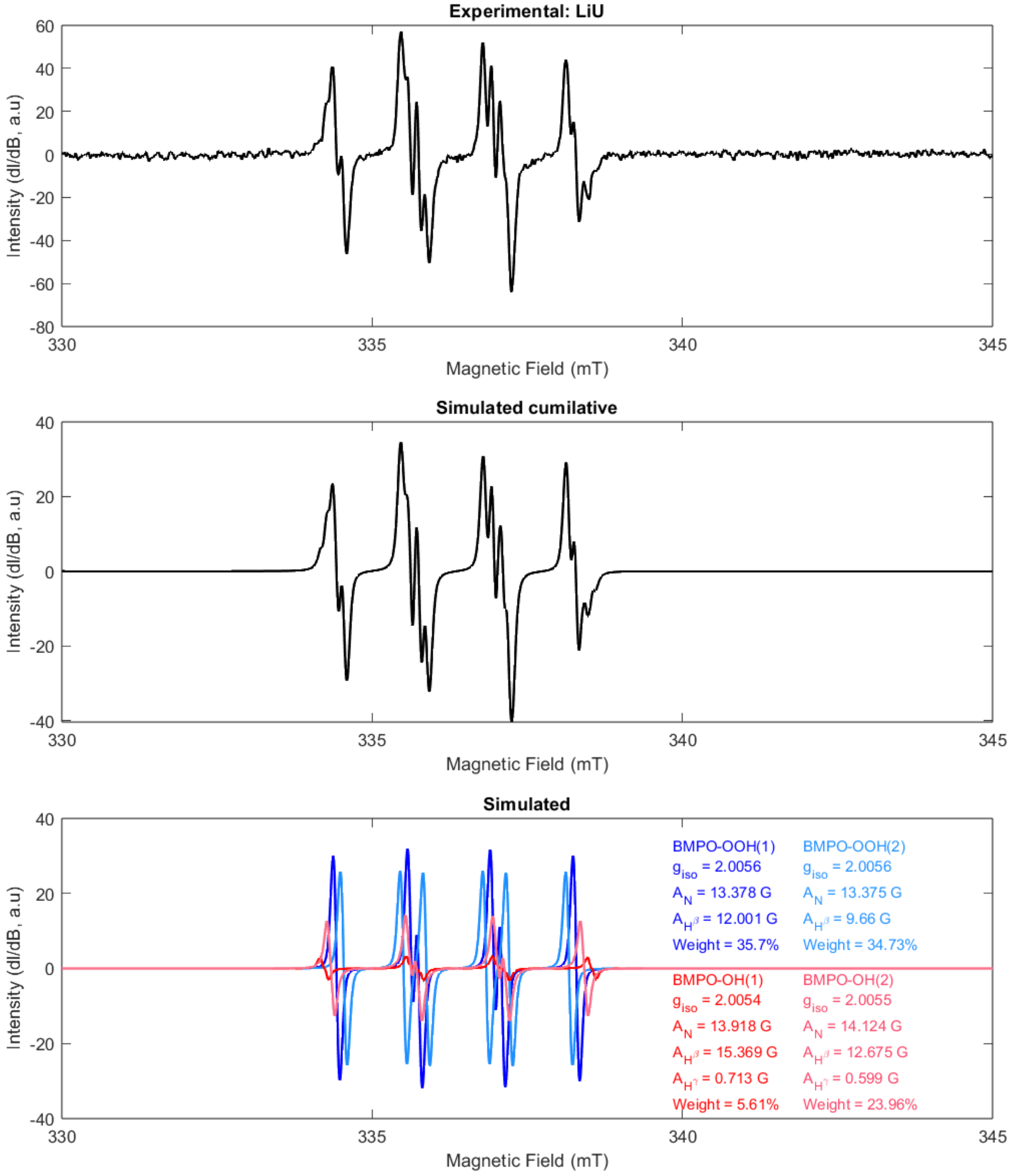

Figure S9: Experimental and simulated EPR spectra 5 mg of **LiU** dissolved in 100 µl of 0.1 M BMPO and 100 µl of 0.1 M DTPA at pH 6

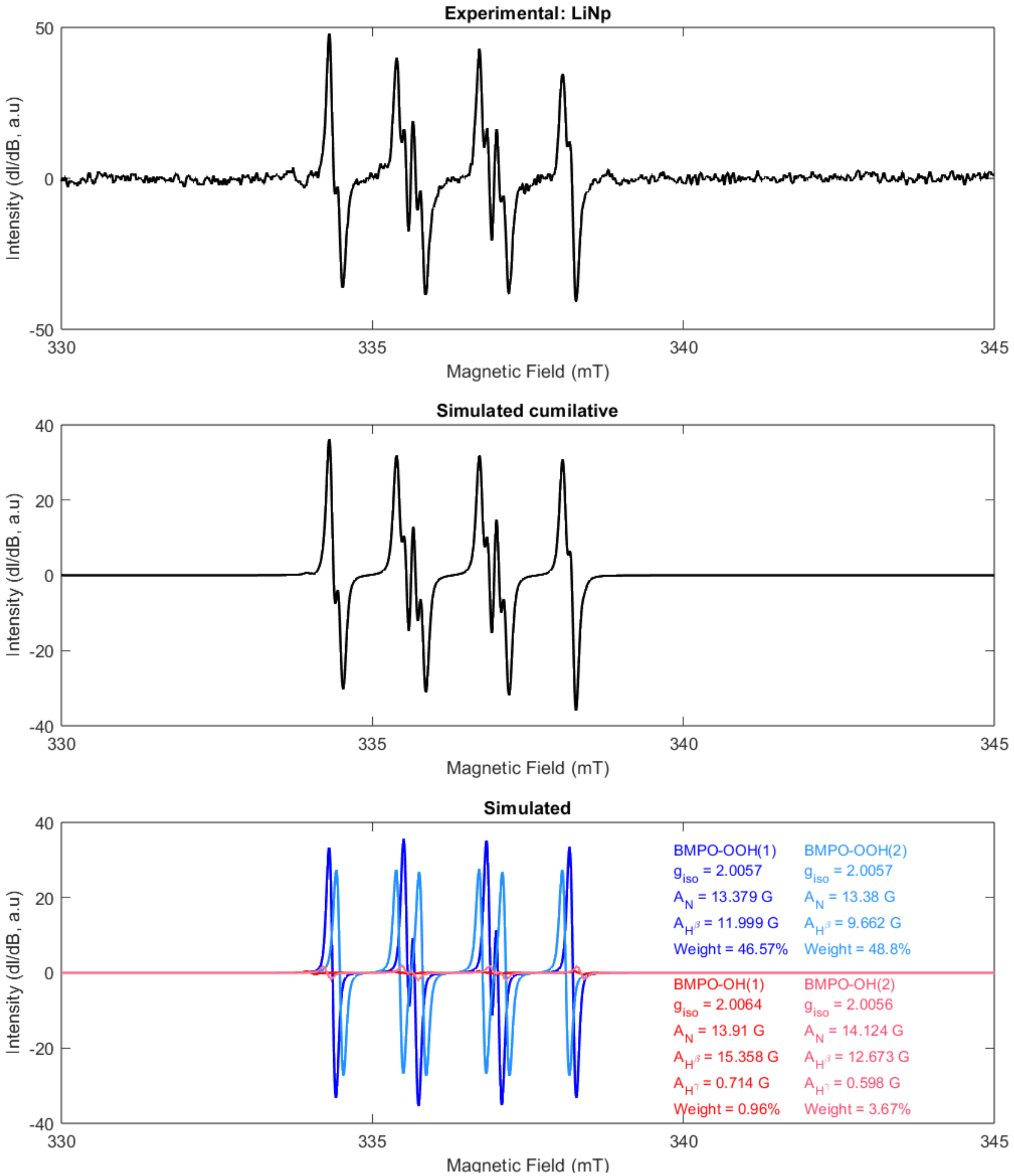

Figure S10: Experimental and simulated EPR spectra of **LiNp** dissolved in 100 µl of 0.1 M BMPO and 100 µl of 0.1 M DTPA at pH 4

4.3 BMPO control experiments

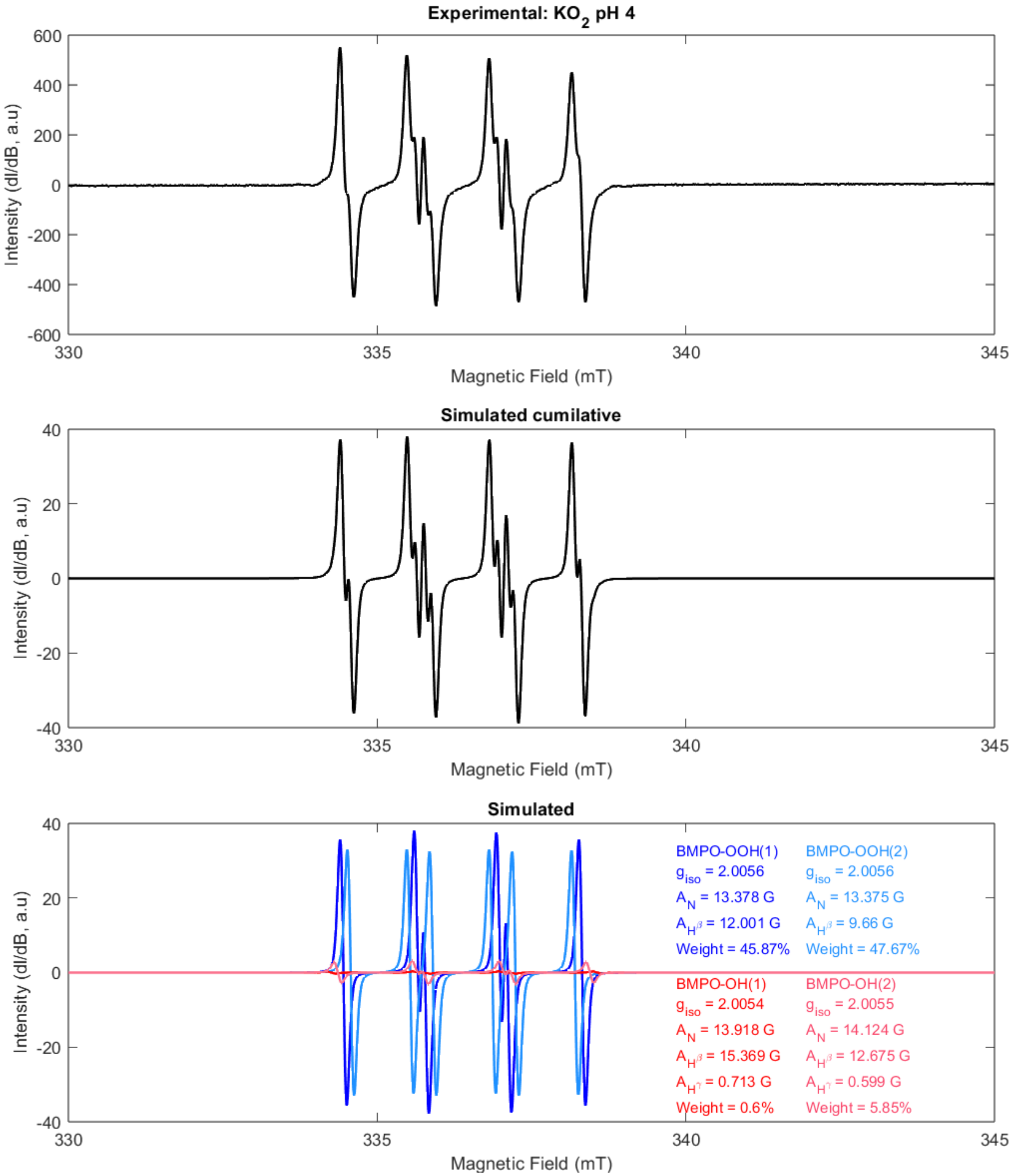

Figure S11: Experimental and simulated EPR spectra of 1 mg KO_2 dissolved in 100 µl of 0.1 M BMPO and 100 µl of 0.1 M DTPA at pH 4 in the presence of 5 mg of uranyl nitrate and 5 mg of LiCl

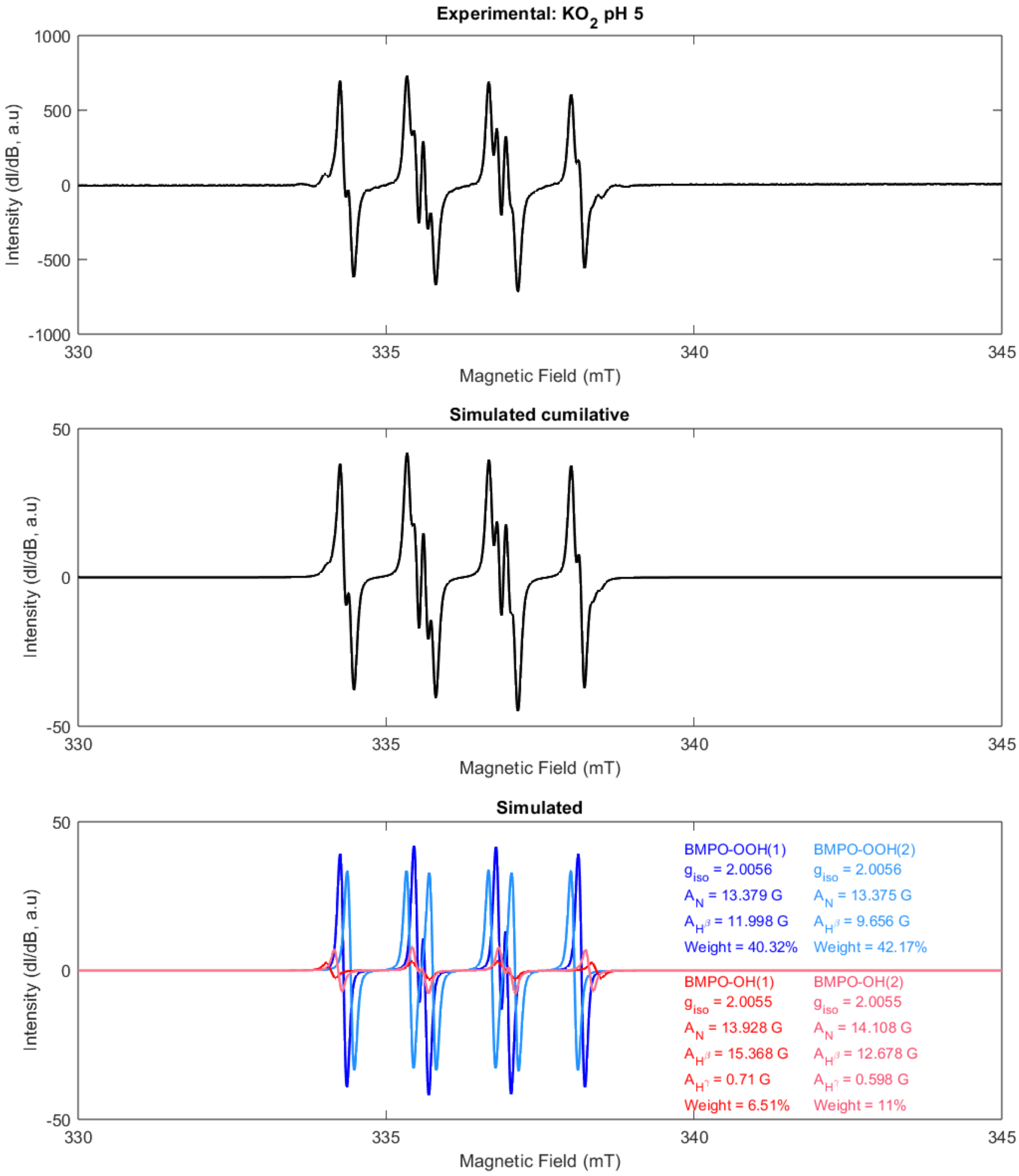

Figure S12: Experimental and simulated EPR spectra of 1 mg KO_2 dissolved in 100 µl of 0.1 M BMPO and 100 µl of 0.1 M DTPA at pH 5 in the presence of 5 mg of uranyl nitrate and 5 mg of LiCl

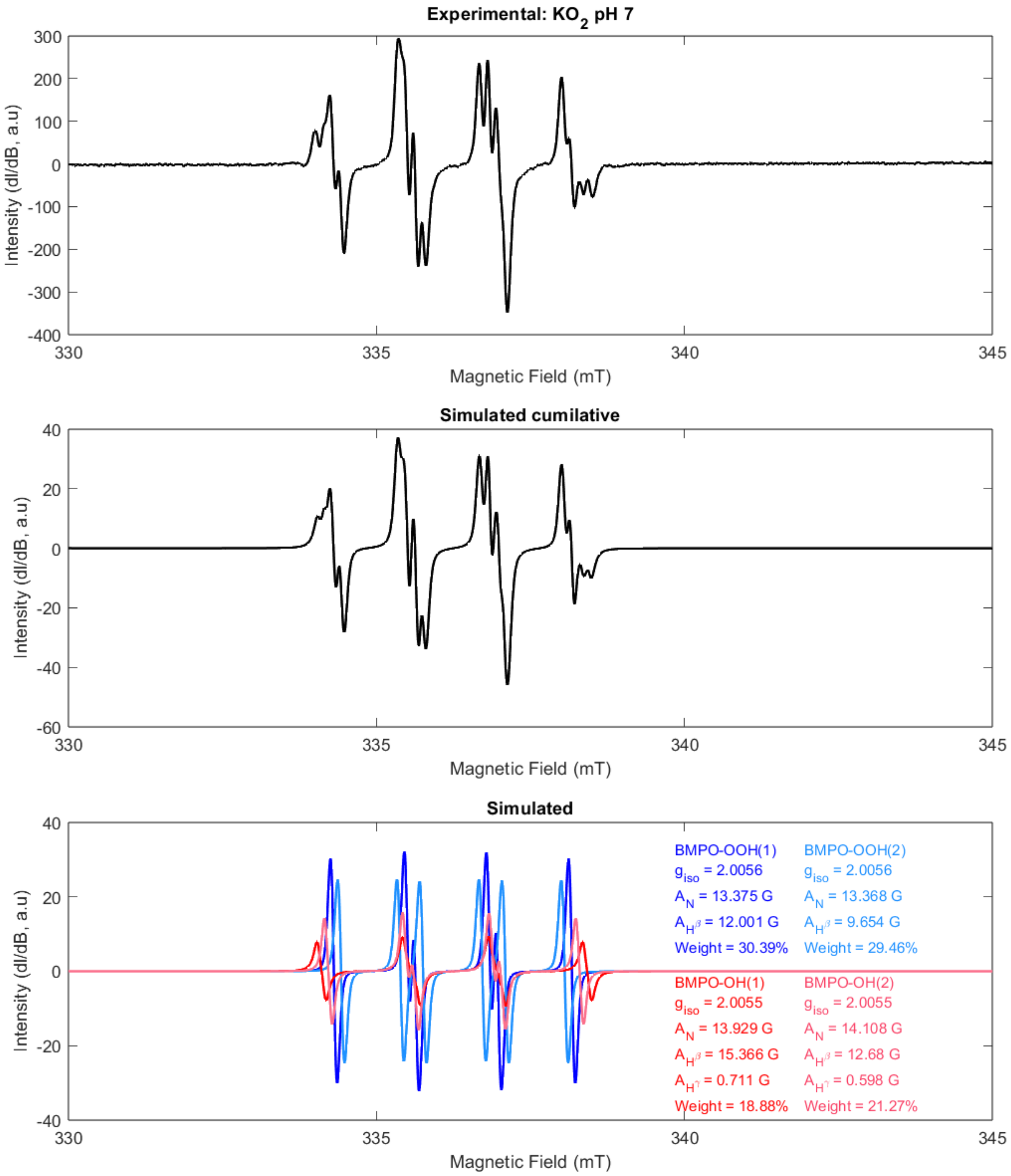

Figure S13: Experimental and simulated EPR spectra of 1 mg KO_2 dissolved in 100 µl of 0.1 M BMPO and 100 µl of 0.1 M DTPA at pH 7 in the presence of 5 mg of uranyl nitrate and 5 mg of LiCl

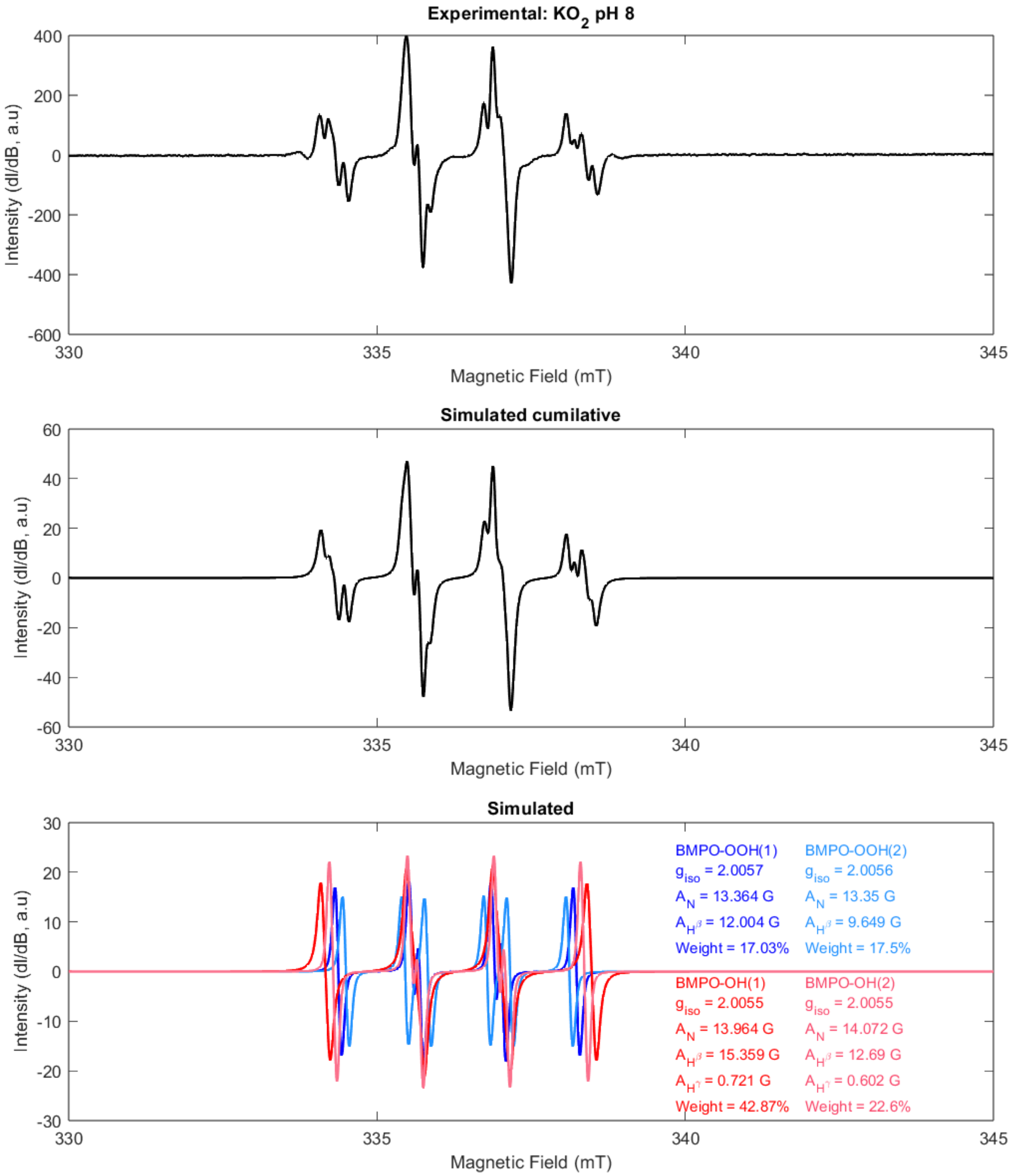

Figure S14: Experimental and simulated EPR spectra of 1 mg KO_2 dissolved in 100 µl of 0.1 M BMPO and 100 µl of 0.1 M DTPA at pH 8 in the presence of 5 mg of uranyl nitrate and 5 mg of LiCl

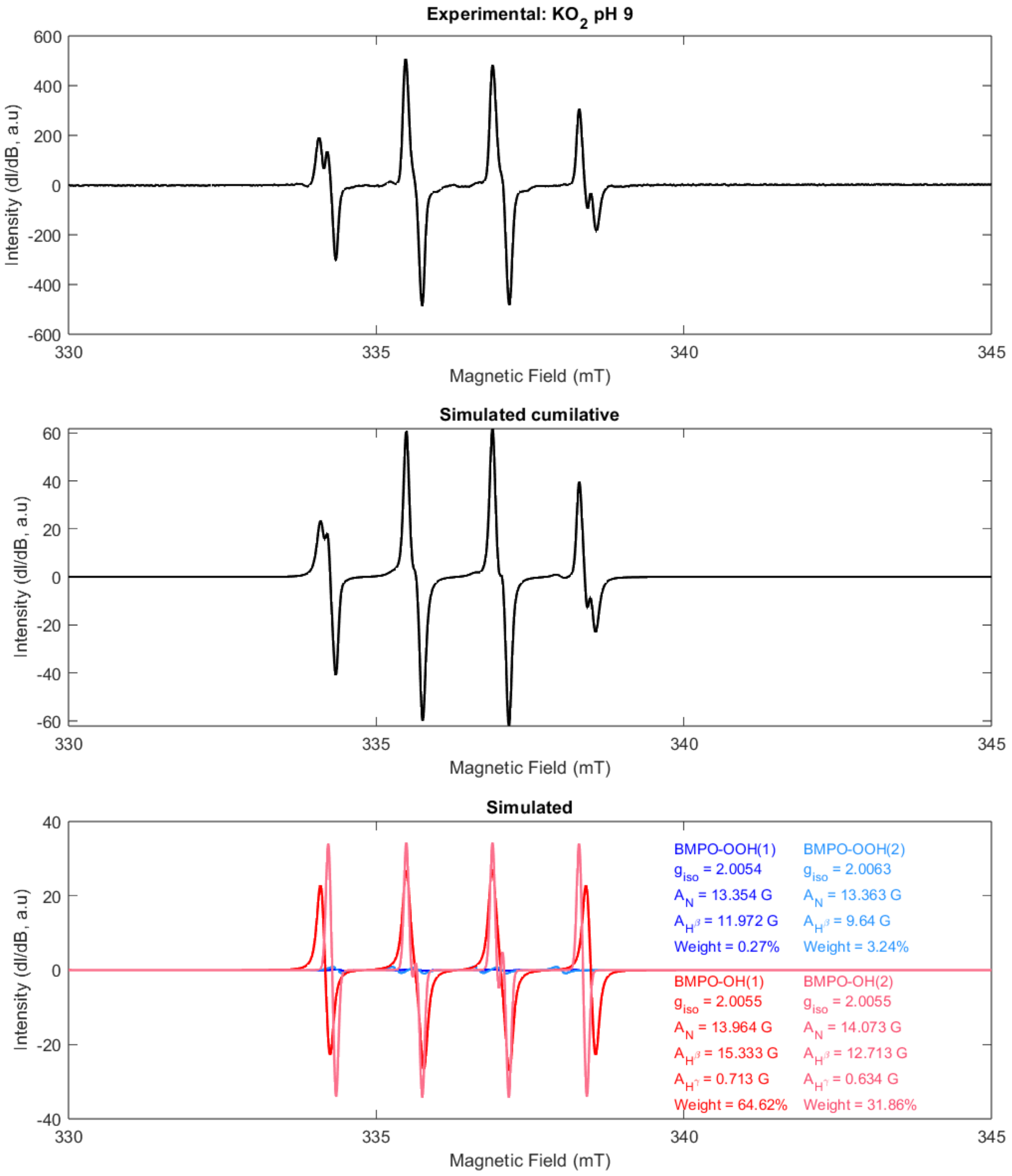

Figure S15: Experimental and simulated EPR spectra of 1 mg KO_2 dissolved in 100 µl of 0.1 M BMPO and 100 µl of 0.1 M DTPA at pH 9 in the presence of 5 mg of uranyl nitrate and 5 mg of LiCl

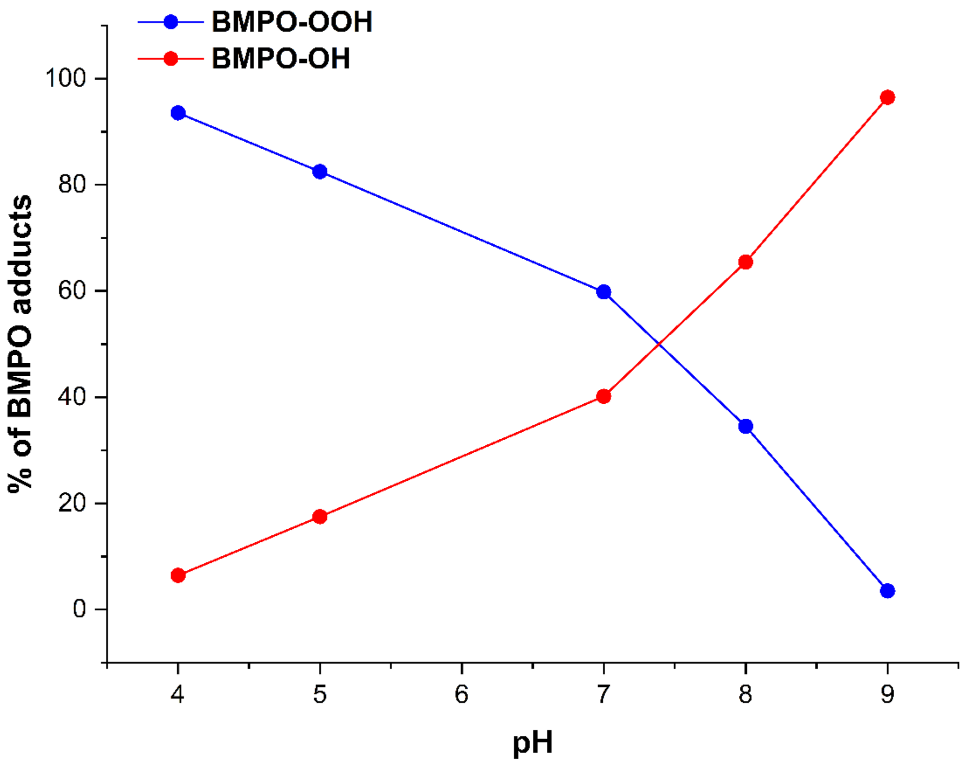

Figure S16:Percentage of BMPO-OOH and BMPO-OH adducts at pH 4,5,7,8,and 9. EPR spectra was collected with1 mg KO_2 dissolved in 100 µl of 0.1 M BMPO and 100 µl of 0.1 M DTPA in the presence of 5 mg of uranyl. 2M HCl or 1M LiOH was added to adjust the pH

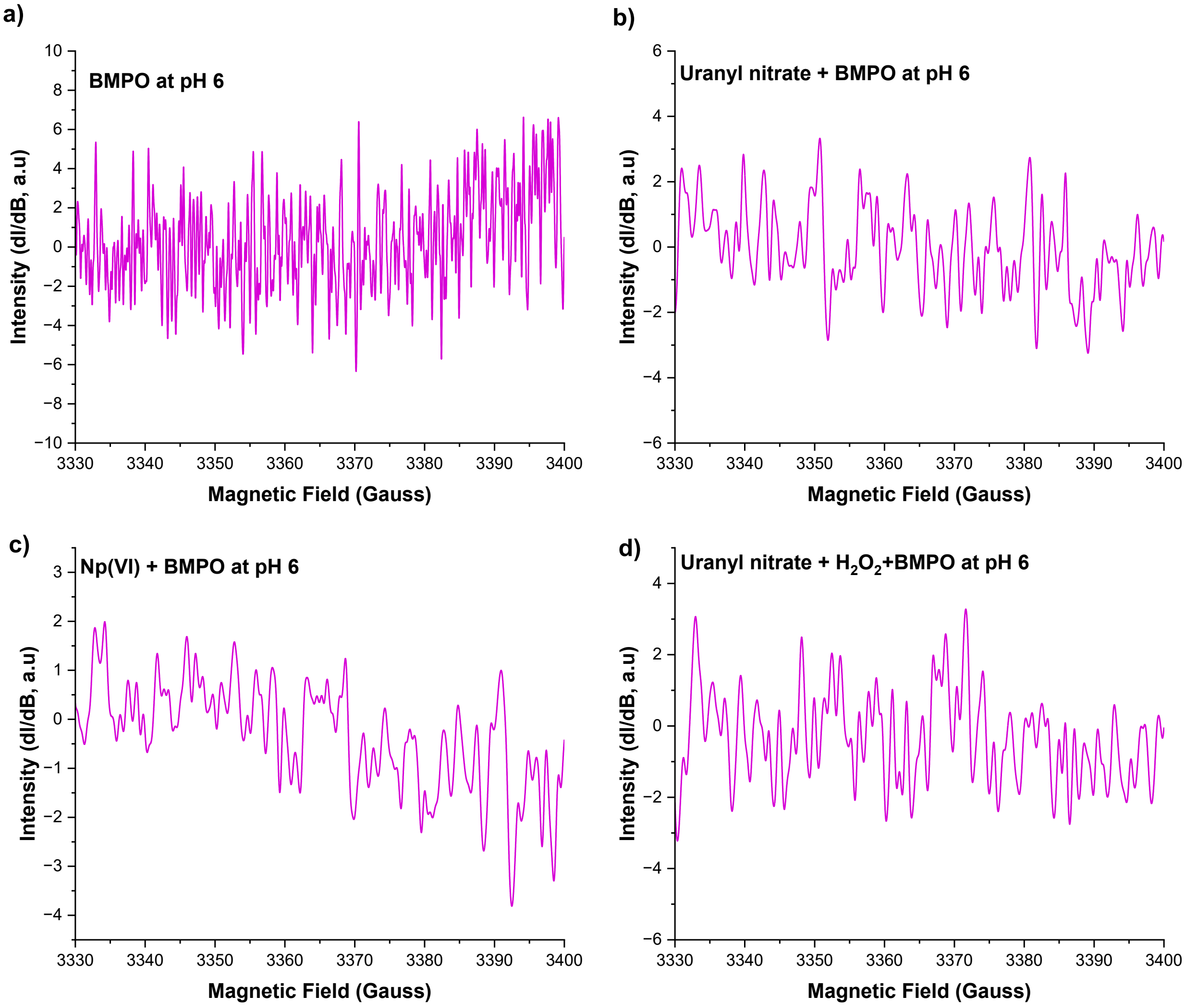

Figure S17: EPR control experiments with **(a)** 100 µl of 0.1 M BMPO + 100 µl of 0.1 M DTPA at pH 6, **(b)** 100 µl of 0.1 M BMPO + 100 µl of 0.1 M DTPA + 25 µl of 0.2 uranyl nitrate at pH 6, **(c)** 100 µl of 0.1 M BMPO + 100 µl of 0.1 M DTPA + 25 µl of 0.2 Np(VI) stock at pH 6, **(d)** 100 µl of 0.1 M BMPO + 100 µl of 0.1 M DTPA + 25 µl of 0.2 uranyl nitrate + 25 µl of 30% H_2O_2 at pH 6. No EPR signatures were observed for any of the control spectra.

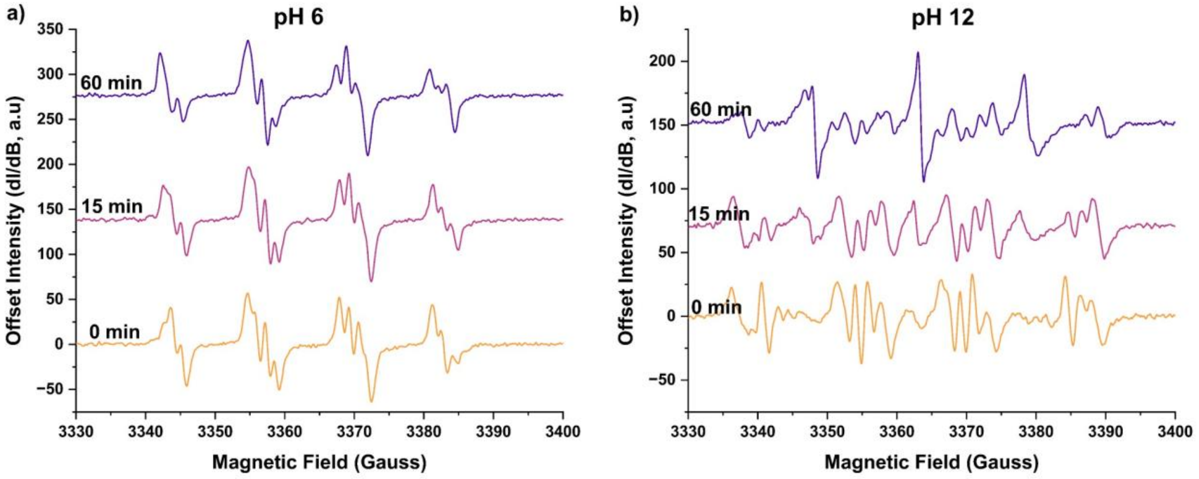

Figure S18: EPR measurements of (a) LiU with BMPO at pH 6 and (b) LiU with BMPO at pH 12 over time. At acidic pH, the BMPO-OOH adducts remain stable until 60 mins. In contrast at alkaline pH, the BMPO adducts undergo degradation when reaching 60 mins.

5. Fitted Raman spectra with fitting parameters

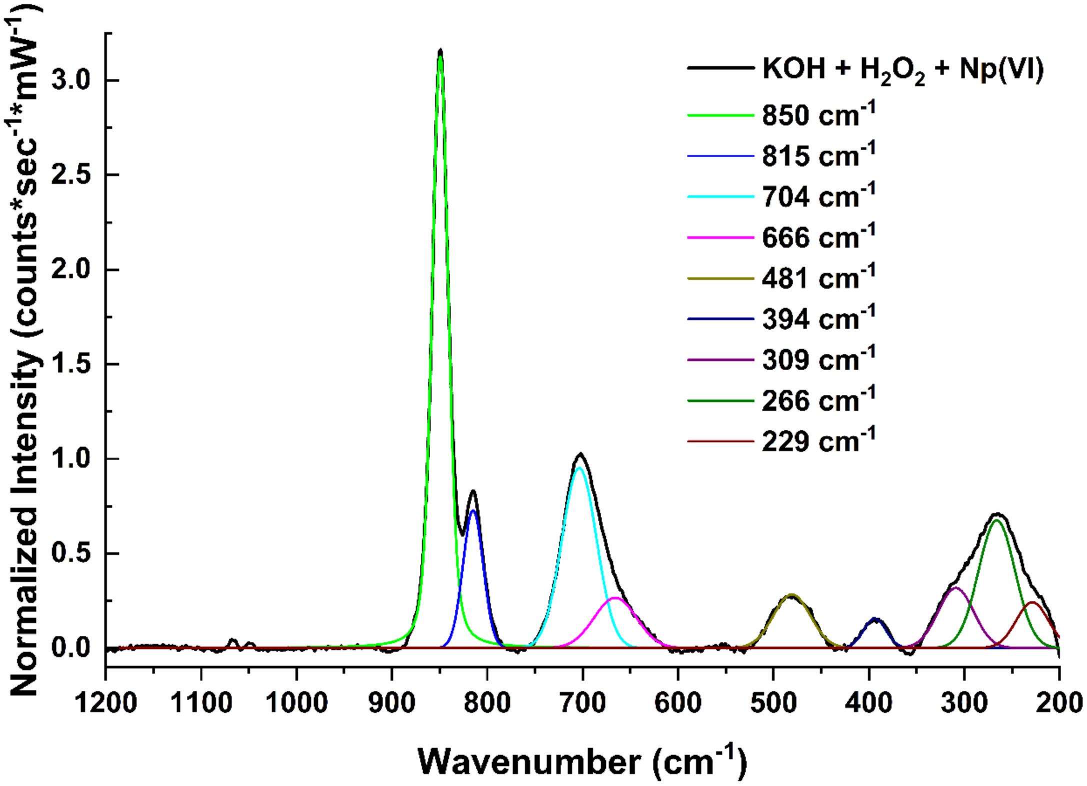

Figure S19: Fitted Raman Sectra of Neptunyl triperoxide solution made with 500µL sat KOH + 500µL 30% H_2O_2 + 500µL H_2O + 100µL of 0.22 M Np(VI). KOH is used instead of LiOH to obtain higher signal without causing solid-state precipitation. The spectral fitting parameters for the spectra are reduced $R^2 = 0.9984$ and reduced $\chi^2 = 3.16 \times 10^{-4}$.

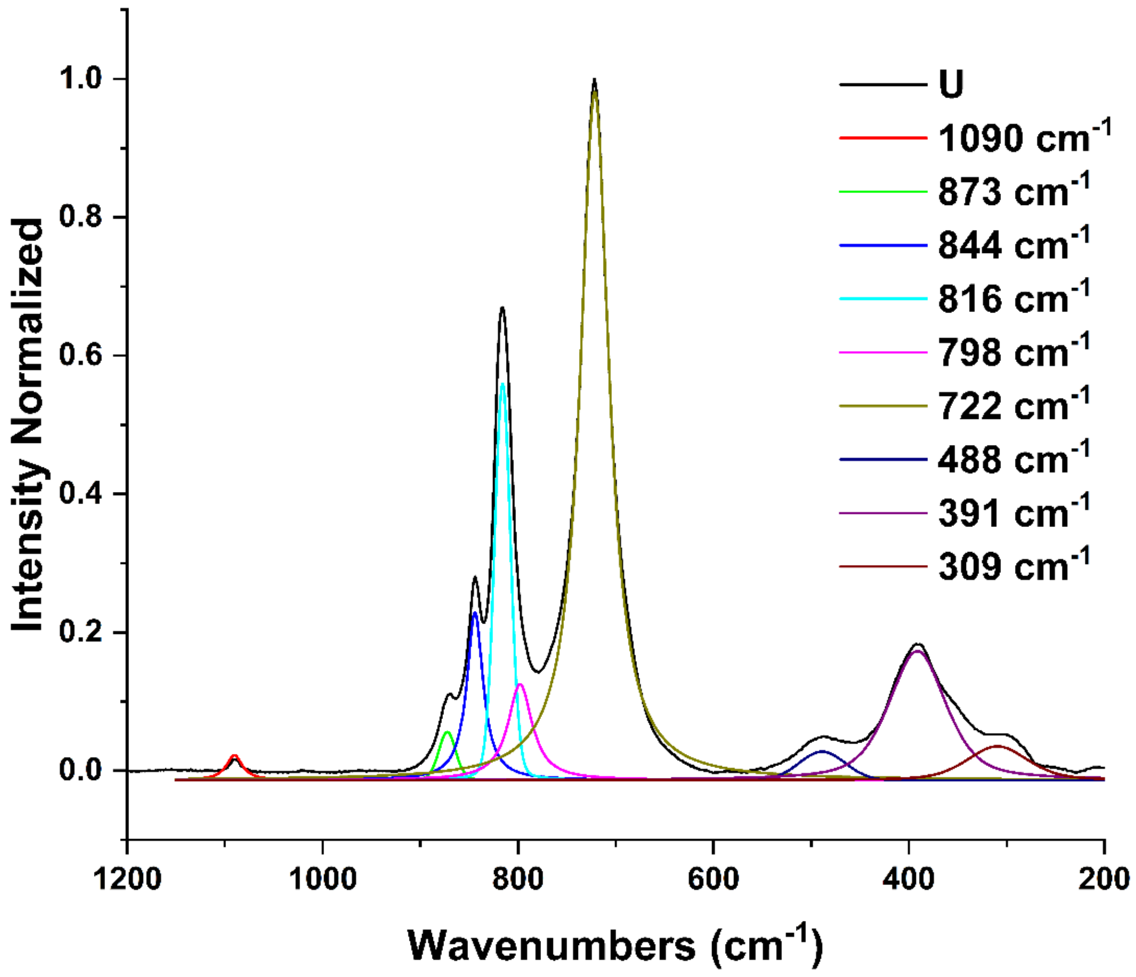

Figure S20: Solid-state Raman spectra of pristine **LiU**. The spectrum was fit with a Pseudo-Voigt function and fitting parameters for the spectra are reduced $R^2 = 0.9984$ and reduced $\chi^2 = 5.73 \times 10^{-5}$.

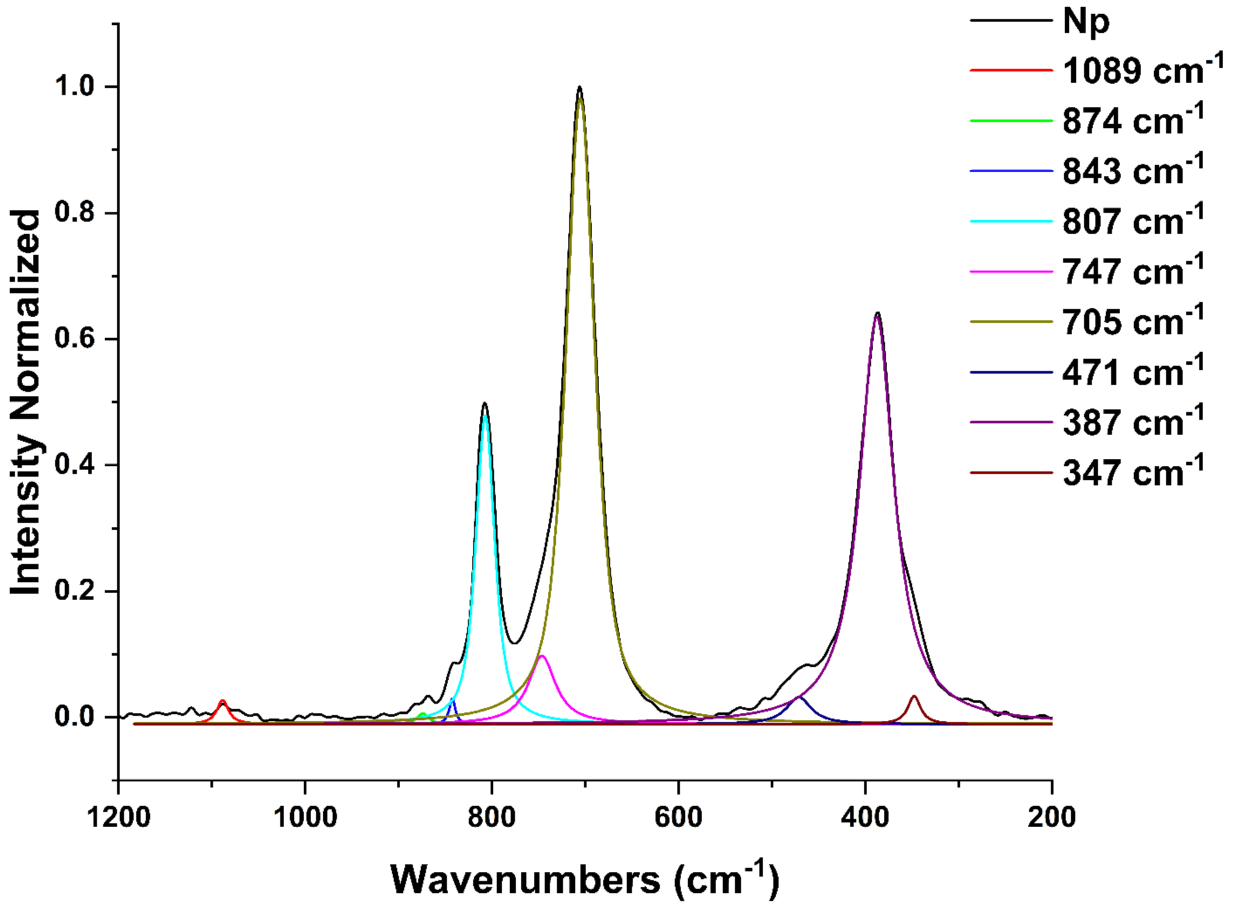

Figure S21: Solid-state Raman spectra of pristine **LiNp**. The spectrum was fit with a Pseudo-Voigt function and fitting parameters for the spectra are reduced R^2 = 0.9981 and reduced χ^2 = 7.68 x 10^{-5}.

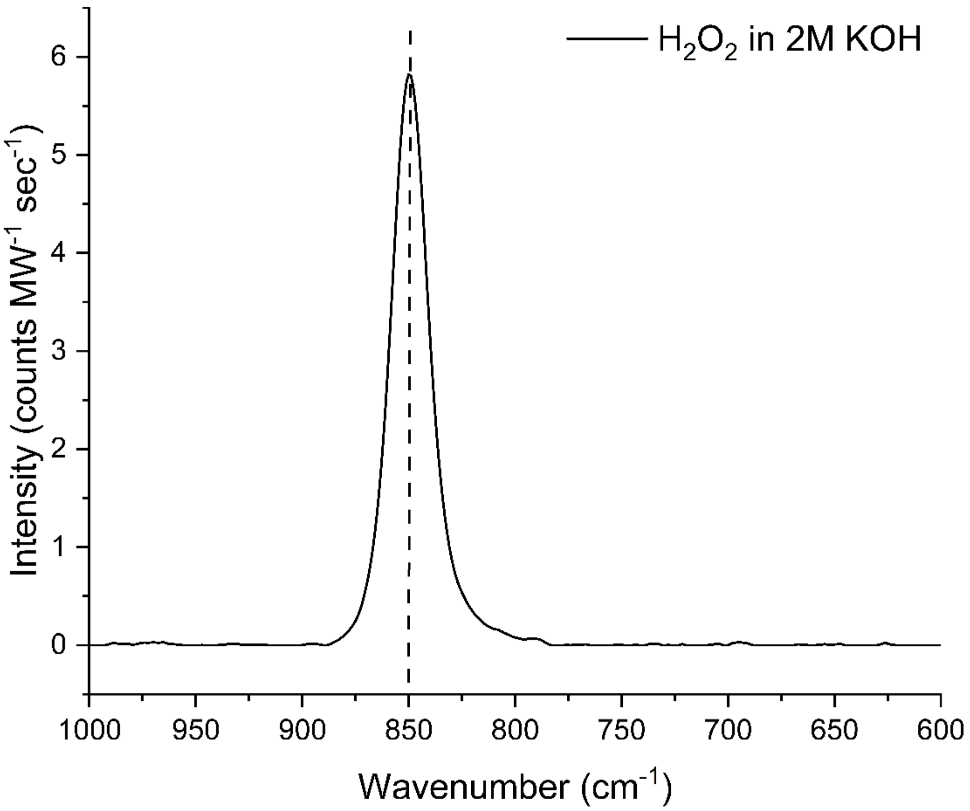

Figure S22: Solution Raman of H_2O_2 in 2 M KOH solution.

6. DFT Calculations Results

6.1 Models I-VI Used in DFT Calculations

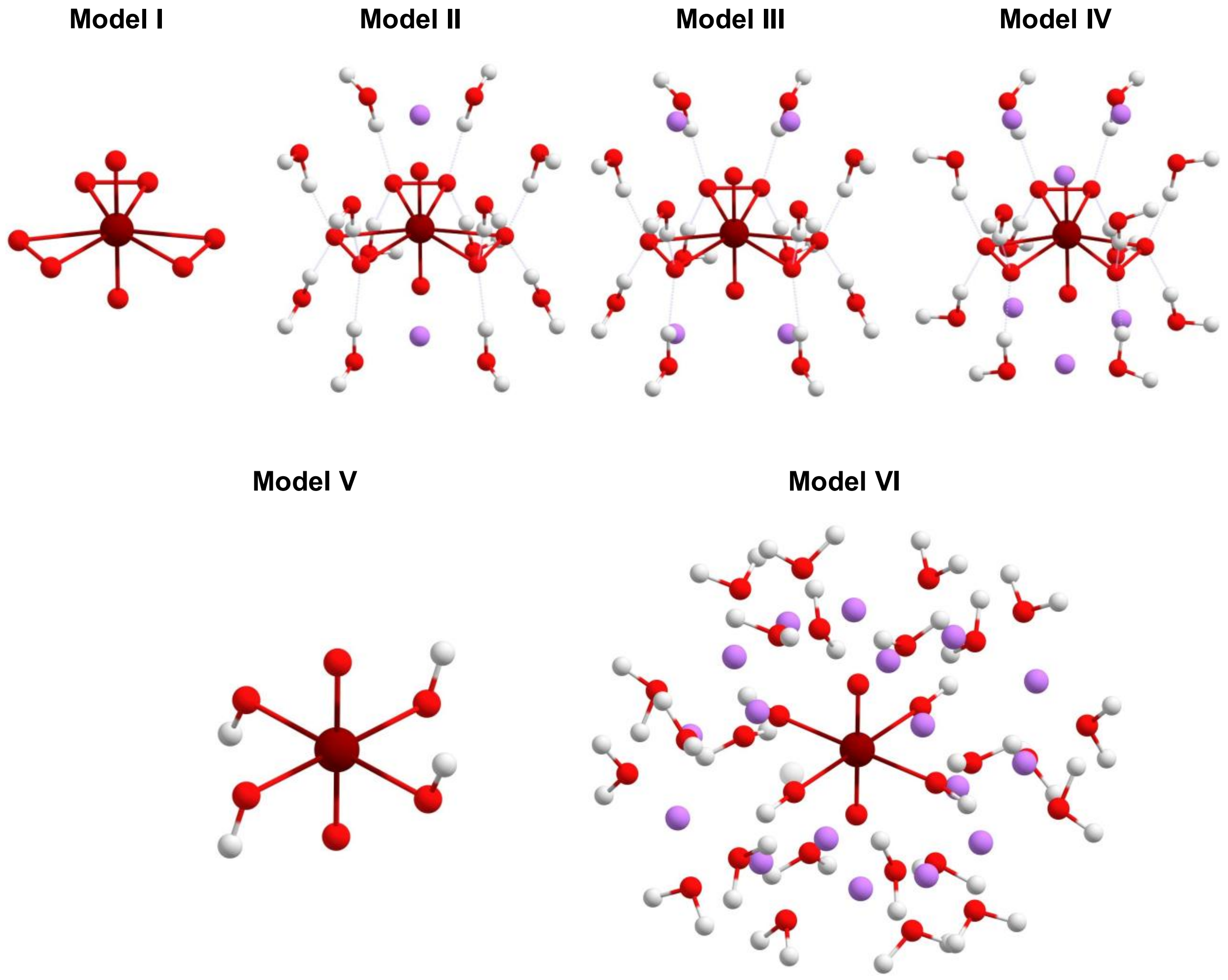

Figure S23: Models I-IV were used in DFT calculations of the actinyl triperoxide units and models V-VI were used in calculation of actinyl tetra hydroxide unit. The maroon, red, purple, and white spheres represent U or Np, O, Li, and H atoms, respectively.

6.2 DFT Calculated Bond Lengths, Vibration Frequencies and Energetics

Table S3: Summary of DFT calculated bond lengths and vibration frequencies for $[UO_2(O_2)_3]^{4-}$ and $[UO_2(O_2)_2O_2^{\bullet}]^{3-}$ species with different second-sphere coordination environments (Model I-IV).

	Molecular property	Model I	Model II	Model III	Model IV
$\mathbf{[UO_2(O_2)_3]^{4-}}$	U=O bond length (Å)	1.903	1.873	1.931	2.035
	$U\text{-}O_2$ bond length (Å)	2.326	2.344	2.314	2.386
	O-O bond length (Å)	1.478	1.469	1.465	1.457
	UO_2^{2+} ν_1 stretch (cm^{-1})	646	719	637	552
	O_2^{2-} ν_3, ν_2, ν_1 stretch (cm^{-1})	837, 842, 872	873, 881, 910	867, 878, 909	885, 889, 927
	UO_2^{2+}– O_2^{2-} stretch (cm^{-1})	377, 370, 356, 337	387, 385, 366, 355	398, 384, 364, 354	420, 409, 385, 375
$\mathbf{[UO_2(O_2)_2O_2^{\bullet}]^{3-}}$	U=O bond length (Å)	1.871	1.858	1.928	1.998
	$U\text{-}O_2$ bond length (Å)	2.294	2.310	2.345	2.224
	O-O bond length (Å)	1.475	1.464	1.456	1.449
	$U\text{-}O_2^{\bullet}$ bond length (Å)	2.734	2.472	2.464	2.405
	$O\text{-}O^{\bullet}$ bond length (Å)	1.334	1.320	1.321	1.315
	UO_2^{2+} ν_1 stretch (cm^{-1})	694	727	625	575
	O_2^{2-} ν_1 stretch (cm^{-1})	838, 870	876, 902	890, 916	890, 923
	$O_2^{\bullet-}$ ν_1 stretch (cm^{-1})	1188	1196	1199	1191
	$g_{\parallel}$	2.063	2.055	2.057	2.058
	$g_{\perp}$	2.018	2.038	2.036	2.038

Table S4: Summary of DFT calculated bond lengths and vibration frequencies for $[NpO_2(O_2)_3]^{4-}$ and $[NpO_2(O_2)_2O_2^{\bullet}]^{3-}$ species with different second-sphere coordination environments (Model I-IV).

	**Molecular property**	**Model I**	**Model II**	**Model III**	**Model IV**
$\mathbf{[NpO_2(O_2)_3]^{4-}}$	Np=O bond length (Å)	1.869	1.845	1.903	2.004
	Np-O_2 bond length (Å)	2.330	2.339	2.320	2.264
	O-O bond length (Å)	1.477	1.463	1.465	1.450
	NpO_2^{2+} ν_1 stretch (cm^{-1})	655	712	609	538
	O_2^{2-} ν_1 stretch (cm^{-1})	843, 848, 889	865, 871, 907	868, 878, 916	882, 892, 935
	NpO_2^{2+}– O_2^{2-} stretch (cm^{-1})	374, 354, 348, 334	372, 360, 354, 345	385, 375, 360, 354, 347	398, 389, 360, 353, 342
$\mathbf{[NpO_2(O_2)_2O_2^{\bullet}]^{3-}}$	Np=O bond length (Å)	1.841	1.831	1.895	1.967
	Np-O_2 bond length (Å)	2.268	2.311	2.280	2.230
	O-O bond length (Å)	1.462	1.456	1.455	1.439
	Np-$O_2^{\bullet}$ bond length (Å)	2.525	2.466	2.436	2.404
	O-$O^{\bullet}$ bond length (Å)	1.325	1.320	1.318	1.316
	NpO_2^{2+} ν_1 stretch (cm^{-1})	699	723	638	563
	O_2^{2-} ν_1 stretch (cm^{-1})	834, 888	866, 909	875, 920	885, 935
	$O_2^{\bullet -}$ ν_1 stretch (cm^{-1})	1193	1194	1193	1190
	g_x	0.656	0.565	0.576	0.668
	g_y	1.244	1.273	1.230	1.140
	g_z	1.528	1.561	1.505	1.417

Table S5: Summary of DFT calculated bond lengths and vibration frequencies for $[UO_2(OH)_4]^{2-}$ and $[UO_2(OH)_4]^{-}$ species with different second-sphere coordination environments (Model V and VI).

	Molecular property	Model V	Model VI
$[UO_2(OH)_4]^{2-}$	U=O bond length (Å)	1.839	1.775
	U-OH bond length (Å)	2.282	2.297
	UO_2^{2+} ν_1 stretch (cm^{-1})	766	861
$[UO_2(OH)_4]^{-}$	U=O bond length (Å)	1.823	1.776
	U-OH bond length (Å)	2.209	2.297
	UO_2^{2+} ν_1 stretch (cm^{-1})	770	893
	g_x	1.230	2.008
	g_y	1.629	2.009
	g_z	1.986	2.011

Table S6: Summary of DFT calculated bond lengths and vibration frequencies for $[NpO_2(OH)_4]^{2}$ and $[NpO_2(OH)_4]^{-}$ species with different second-sphere coordination environments (Model V and VI).

	Molecular property	Model V	Model VI
$[NpO_2(OH)_4]^{2-}$	Np=O bond length (Å)	1.816	1.754
	Np-OH bond length (Å)	2.267	2.286
	NpO_2^{2+} stretch ν_1 (cm^{-1})	759	855
$[NpO_2(OH)_4]^{-}$	Np=O bond length (Å)	1.798	1.755
	Np-OH bond length (Å)	2.195	2.283
	NpO_2^{2+} stretch ν_1 (cm^{-1})	776	875
	g_x	0.505	0.899
	g_y	1.277	1.402
	g_z	1.347	1.406

Table S7: Energetics comparing the relative stabilities of $[NpO_2(O_2)_2(O)_2^{\bullet}]^{3-}$ and $[UO_2(O_2)_2(O)_2^{\bullet}]^{3-}$ species within the crystalline matrix.

**Relative stability via :** $[UO_2(O_2)_2(O_2)^{\bullet}]^{3-} + [NpO_2(O_2)_3]^{4-} \rightarrow [UO_2(O_2)_3]^{4-} + [NpO_2(O_2)_2(O_2)^{\bullet}]^{3-}$	**ΔG (kJ/mol)**
Model II	4.95
Model III	-11.93
Model IV	6.34

6.3 Selected Vibration Modes

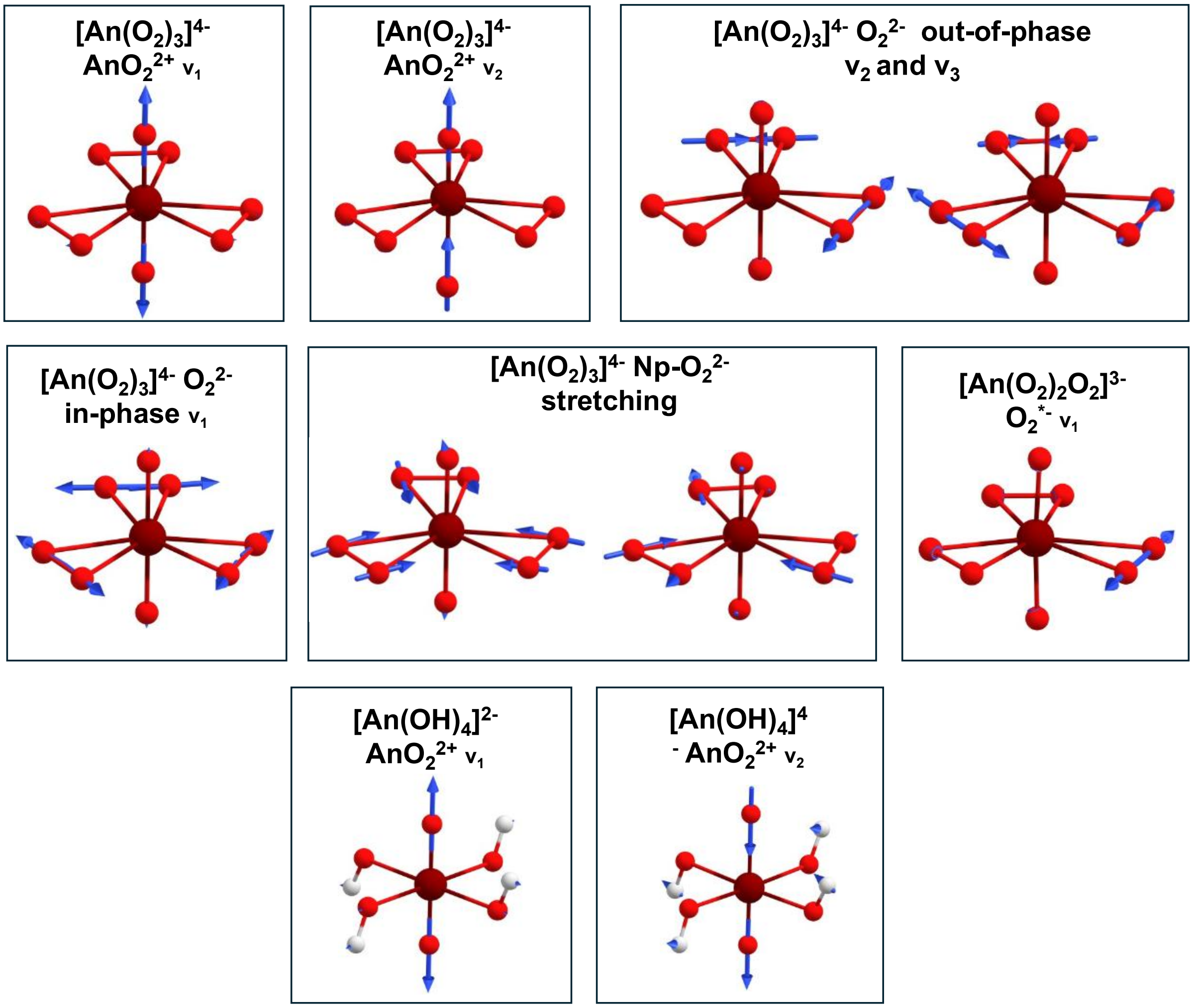

Figure S24: Selected vibrational modes of actinyl triperoxide and actinyl tetrahydroxide moieties. The maroon, red, and white spheres represent actinyl (U or Np), O, and H atoms respectively. The blue arrow represents the displacement vectors of atoms with different vibrational modes

6.4 Molecular Orbital and Spin Density Analysis

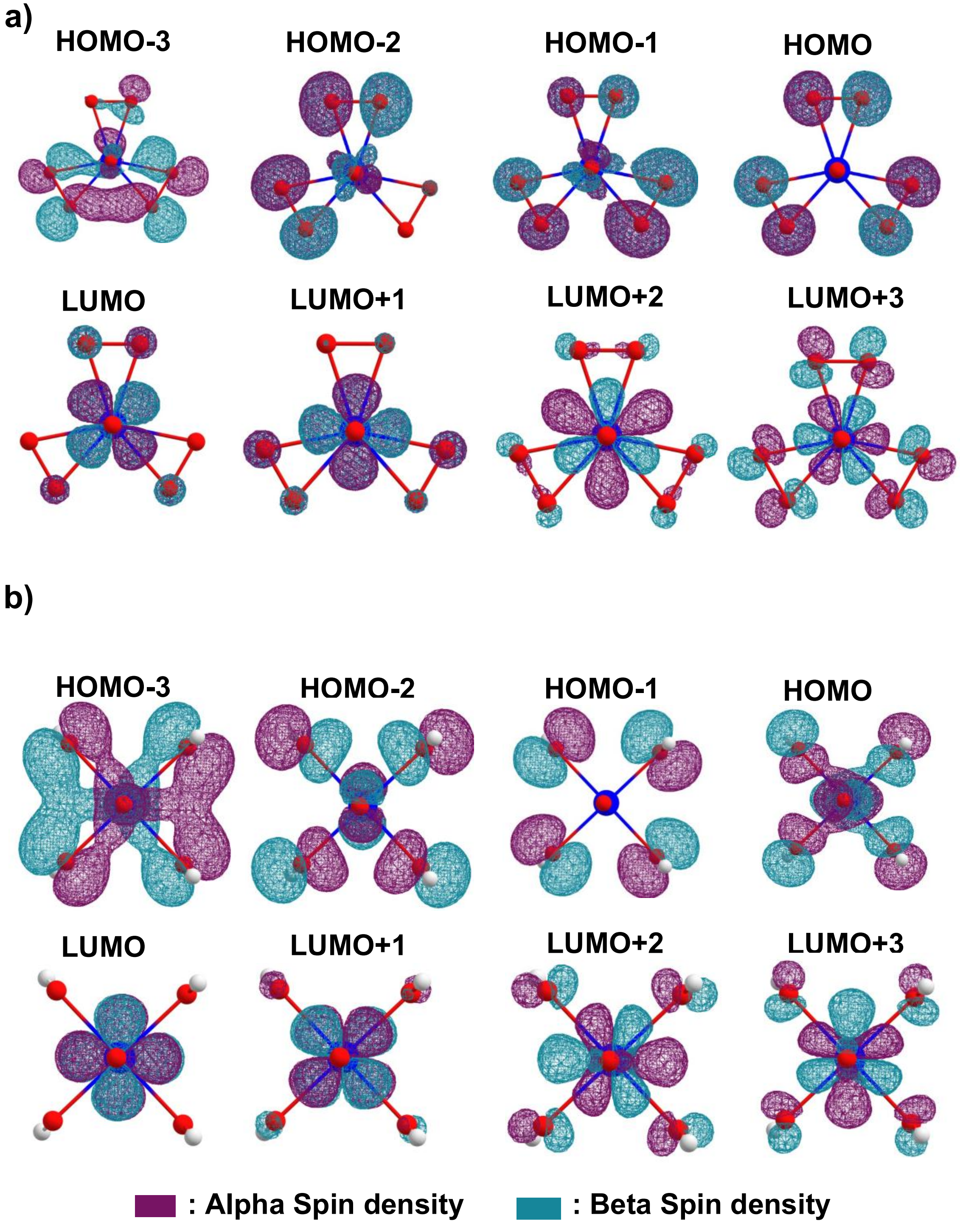

Figure S25: HOMO-3 to LUMO+3 molecular orbitals of **(a)** $[UO_2(O_2)_3]^{4-}$ and **(b)** $[UO_2(OH)_4]^{2-}$ units from the DFT calculations. The blue, red, and white spheres represent U, O, and H atoms, respectively. Surfaces were generated with iso value of 0.030.

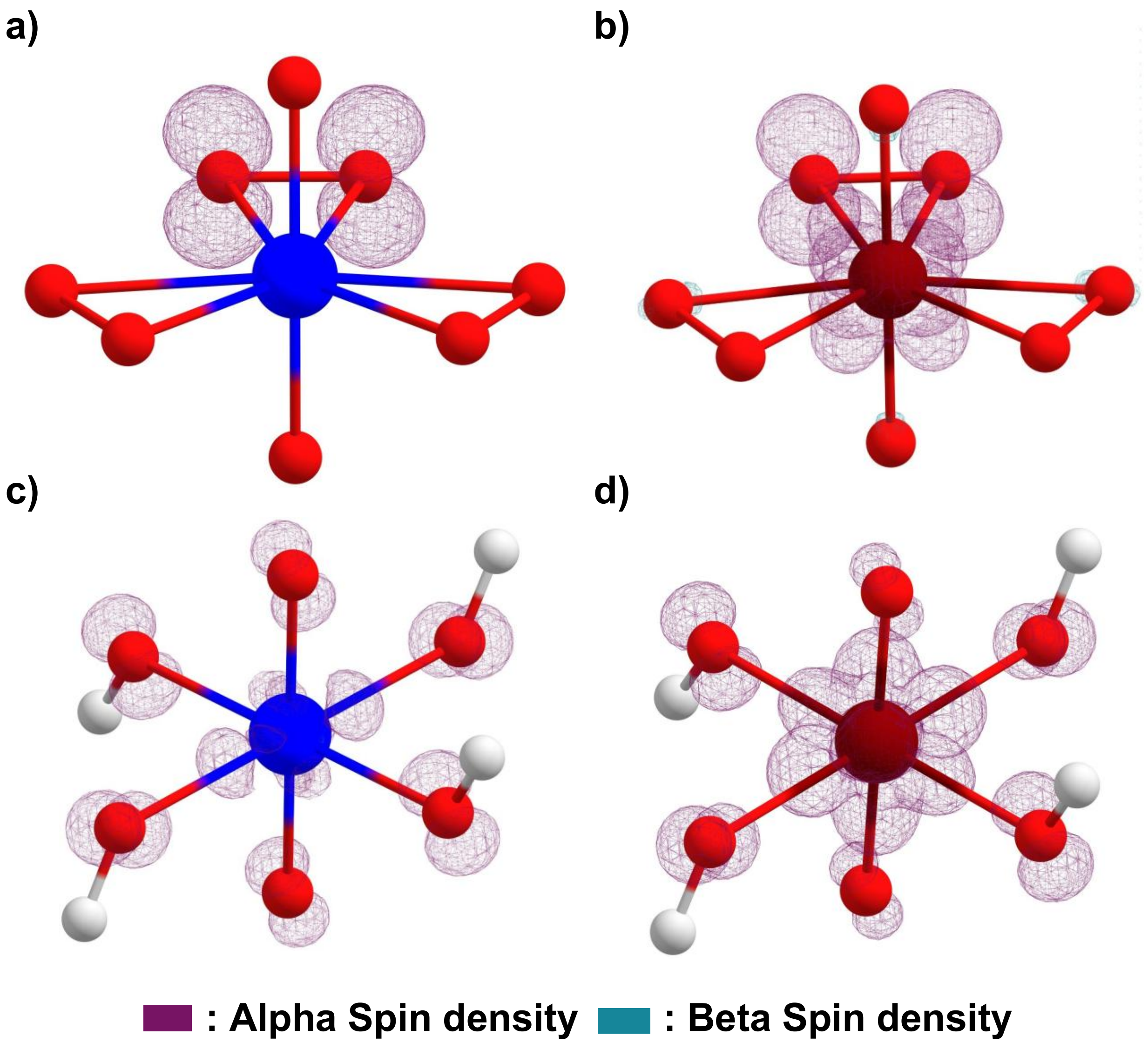

Figure S26: Spin density of **(a)** $[UO_2(O_2)(O_2)^{\bullet}]^{3-}$, **(b)** $[NpO_2(O_2)_2(O_2)^{\bullet}]^{3-}$, **(c)** $[UO_2(OH)_4]^{-}$, and **(d)** $[NpO_2(OH)_4]^{-}$ moieties determined from the DFT calculations. The blue, maroon, red, and white spheres represent U, Np, O, and H atoms respectively. The surfaces were generated with iso value of 0.015.

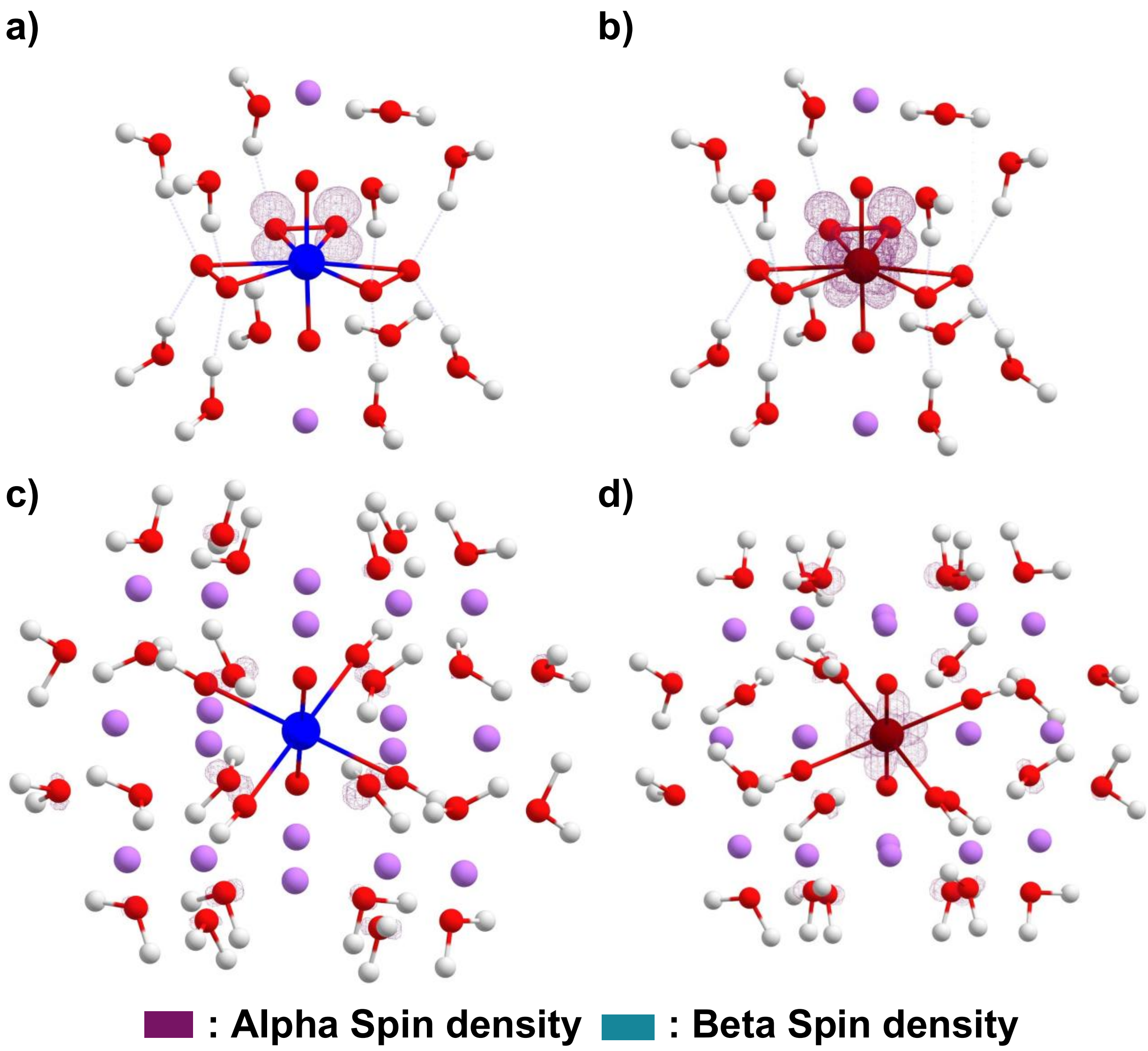

Figure S27: Spin density of **(a)** $[UO_2(O_2)(O_2)^{\bullet}]^{3-}$, **(b)** $[NpO_2(O_2)_2(O_2)^{\bullet}]^{3-}$, **(c)** $[UO_2(OH)_4]^{-}$, and **(d)** $[NpO_2(OH)_4]^{-}$ complexes with inclusion of the secondary coordination sphere. The blue, maroon, red, purple, and white spheres represent U, Np, O, Li, and H atoms, respectively. The surfaces were generated with iso value of 0.015.

6.5 QTAIM Analysis

Table S8: QTAIM on the $[AnO_2(O_2)_3]^{4-}$ unit. The U, Np, and Np-U column represent results for $[UO_2(O_2)_3]^{4-}$, $[NpO_2(O_2)_3]^{4-}$ and the difference between the two respectively. Here topological Parameters of Electron Density are Electron Density (***ρ***), Its Laplacian ($\nabla^2\rho$), Local Kinetic Energy Density (***G***), Potential Energy Density (***V***) and Electronic Energy Density (***H***). All data are given in arbitrary units

**Bond**	***ρ***			$\nabla^2\rho$			***ε***			***G***			***V***			***H***		
	**U**	**Np**	**Np-U**	**U**	**Np**	**Np-U**	**U**	**Np**	**Np-U**	**U**	**Np**	**Np-U**	**U**	**Np**	**Np-U**	**U**	**Np**	**Np-U**
**An=O**	0.221	0.238	0.016	0.341	0.329	-0.013	0.000	0.003	0.002	0.232	0.249	0.016	-0.379	-0.415	-0.036	-0.147	-0.166	-0.019
**An-O_2^{2-}**	0.080	0.078	-0.003	0.254	0.252	-0.002	0.132	0.179	0.048	0.079	0.076	-0.002	-0.094	-0.090	0.004	-0.015	-0.013	0.002
**O-O**	0.252	0.255	0.002	0.200	0.195	-0.006	0.037	0.035	-0.002	0.192	0.194	0.001	-0.334	-0.339	-0.004	-0.142	-0.145	-0.003

Table S9: QTAIM on the $[AnO_2(OH)_4]^{2-}$ unit. The U, Np, and Np-U column represent results for $[UO_2(OH)_4]^{2-}$, $[NpO_2(OH)_4]^{2-}$ and the difference between the two respectively. Here topological Parameters of Electron Density are Electron Density (***ρ***), Its Laplacian ($\nabla^2\rho$), Local Kinetic Energy Density (***G***), Potential Energy Density (***V***) and Electronic Energy Density (***H***). All data are given in arbitrary units

**Bond**	***ρ***			$\nabla^2\rho$			***ε***			***G***			***V***			***H***		
	**U**	**Np**	**Np-U**	**U**	**Np**	**Np-U**	**U**	**Np**	**Np-U**	**U**	**Np**	**Np-U**	**U**	**Np**	**Np-U**	**U**	**Np**	**Np-U**
**An=O**	0.260	0.272	0.012	0.334	0.318	-0.015	0.000	0.000	0.000	0.287	0.298	0.011	-0.490	-0.516	-0.027	-0.203	-0.218	-0.015
**An-OH**	0.089	0.090	0.001	0.279	0.282	0.003	0.069	0.068	-0.002	0.088	0.089	0.001	-0.107	-0.107	0.000	-0.019	-0.018	0.000

6.6 DFT optimized coordinates

Table S10: DFT optimized coordinates of Model I - $[UO_2(O_2)_3]^{4-}$

U	0.12884425483745	-0.07353370896836	0.08961043274510
O	1.43083089362818	1.31387246285121	0.07948875074139
O	-1.16737432577963	-1.46721882602386	0.09574420922442
O	-0.35197279587859	0.38412712771538	2.31862413380216
O	-1.23669368424909	1.20920647636108	1.46745137336301
O	1.78357524360571	-1.61804450123116	-0.44708240822512
O	1.69084552647139	-1.51772589381644	1.02752980824983
O	-0.06850437789468	0.09253136074143	-2.22116231438435
O	-1.04912976274075	1.01217153637072	-1.59908738451644

Table S11: DFT optimized coordinates of Model I - $[UO_2(O_2)_2(O_2)^{\bullet}]^{3-}$

U	0.00905339127925	0.03770212142262	0.07541338465616
O	1.38532385440974	1.30457174438278	0.07992030282338
O	-1.15697416026194	-1.42488367153961	0.09746969815715
O	-0.41214705510403	0.44431769093466	2.30089300310888
O	-1.30842332197701	1.27458328218656	1.47535613898385
O	1.98279324028806	-1.80382886861000	-0.35031004355525
O	1.89998746583775	-1.71862328959540	0.97812874942038
O	-0.12653544462355	0.14955382695893	-2.21908886280723
O	-1.11265699784827	1.07199319785946	-1.62666576978731

Table S12: DFT optimized coordinates of Model I - $[NpO_2(O_2)_3]^{4-}$

Np	0.17186774846846	-0.09532885882965	-0.01274098209055
O	1.44264256047078	1.27468056501258	-0.01321505373490
O	-1.09845820721869	-1.46673847025830	-0.01411452415666
O	-0.17869473898115	0.22824690512102	2.24423920765363
O	-1.12267344867434	1.10034774048046	1.52780639832818
O	1.80296029420918	-1.59678769402747	-0.73397613427765
O	1.80388533923051	-1.59673554691286	0.75059790209549
O	-0.17607488836586	0.22611828092833	-2.26762267931316
O	-1.12826187513890	1.10518915848588	-1.56317289450438

Table S13: DFT optimized coordinates of Model I - $[NpO_2(O_2)_2(O_2)^{\bullet}]^{3-}$

Np	0.06962930456291	-0.02034236027557	0.08254111208288
O	1.37613046192951	1.27686039639924	0.08012703264129
O	-1.13319267320317	-1.41525344598459	0.09686744356273
O	-0.38026474527280	0.41224085878779	2.24910389911570

O	-1.27661386780378	1.24569351361408	1.44692000353888
O	1.88939206582895	-1.71464987098371	-0.35747804600432
O	1.80662100819741	-1.62737209847399	0.96233054134775
O	-0.10293085304694	0.12827749186088	-2.15984351213742
O	-1.08834972919211	1.04993154905591	-1.58945187314747

Table S14: DFT optimized coordinates of Model II - $[UO_2(O_2)_3]^{4-}$

U	0.18531200693410	-0.08606112808656	-0.00494387269645
O	1.43637750969490	1.30712974585351	-0.04379054111590
O	-1.10874322204819	-1.43966755200033	0.00210399765603
O	-0.15834799403398	0.19413394793947	2.28689993088900
O	-1.08926129918030	1.08967614429709	1.58790917959103
O	1.84561083008943	-1.59109341212369	-0.69694844129540
O	1.81418684970057	-1.57413117133750	0.77244866255323
O	-0.15588097590282	0.17997269892639	-2.30222635567246
O	-1.08447277075953	1.08183199582134	-1.60673419820722
Li	-2.27445847397848	-2.93511305936763	0.10181879653719
Li	2.80464598180142	2.61673587463928	-0.36868282401272
O	-3.52186726161441	-0.24607776162612	-1.79420285549118
O	-3.52185914123919	-0.24605931010960	1.79408860270870
O	-1.54981305379109	-1.93895470065143	3.26530192306678
O	0.28448799009669	-3.75210526410620	1.45220746051998
O	1.93913659154286	1.54967203984431	3.26528997215681
O	3.99538998805467	0.04084578276189	1.48435373183437
O	1.93914982618809	1.54967778810914	-3.26543330440241
O	4.01401712762377	-0.02848315257854	-1.38178390133502
O	-1.54980439568779	-1.93897212723212	-3.26529515922329
O	0.28448832192909	-3.75210583224850	-1.45230828214353
O	0.24628199424724	3.52185284323920	-1.79416882065295
O	0.24633290757169	3.52186394207708	1.79412268944526
H	-1.02734060064112	-1.14583666064300	3.00693772903703
H	0.70922923725541	-4.40124504129549	2.02205089241052
H	-1.03161090689520	-1.14460722936360	-3.00506151079664
H	0.76549593437916	-4.49087196214584	-1.84090319359544
H	4.59579905296025	-0.41303029561687	-2.04967281528245
H	4.63197357703051	-0.10664501135591	0.77185544619882
H	1.64305621199531	2.36118006477555	-3.69275404372801
H	1.66264875375120	2.35792865068275	3.71127078602467
H	-0.28266026583444	4.21142490662507	2.21069924540263
H	1.11711725820612	1.07267471635360	-3.00514545262854
H	1.10777096668593	1.08330956681075	3.02170776905391
H	3.30569756338105	-0.64682308013539	1.33821004062044
H	3.31138175309954	-0.70138906852784	-1.19366769132444

H	-2.36897264699100	-1.58600363706875	3.63189418383727
H	-2.36840697015471	-1.59289577067068	-3.63994875291993
H	0.97154931915489	-3.08764312996252	1.23241187261919
H	0.95073899537755	-3.09252378620328	-1.17691275102137
H	-0.31092227012346	2.70806250834796	1.78921981035365
H	-0.27899737587850	4.20707774207046	-2.22300605357518
H	-0.31329426497015	2.70954562042105	-1.78548741857844
H	-2.70184770491575	0.30448021294134	1.78408216596589
H	-4.21155924048049	0.29797208931349	2.19070035543194
H	-2.70573374645942	0.30920090629592	-1.78535817744855
H	-4.20991060665061	0.28818402174555	-2.20711786642332

Table S15: DFT optimized coordinates of Model II - $[UO_2(O_2)_2(O_2)^{\bullet}]^{3-}$

U	-0.01431118102100	0.04266765933064	0.00840206582409
O	1.31618865584873	1.33780657177976	-0.02020529905866
O	-1.24747187421681	-1.34696064637277	0.00033482679623
O	-0.37981498309439	0.39498943416780	2.25073414496847
O	-1.28149689304826	1.27194074007836	1.50381304170503
O	1.81774427756605	-1.61939813032632	-0.28184263789117
O	1.66812486322752	-1.43097992718122	1.01644324158032
O	-0.23983481723814	0.24731590043691	-2.28870853981369
O	-1.17962320439443	1.16608548326961	-1.64220050061406
Li	-2.51040959187691	-2.91014576823954	-0.20901219924401
Li	2.72776985203159	2.69510678108221	-0.13966066408819
O	-3.52186808910146	-0.24607630114470	-1.79420037837124
O	-3.52185534131939	-0.24606479957599	1.79409841784181
O	-1.54979138816852	-1.93897042557342	3.26529909247466
O	0.28448674056812	-3.75210699627838	1.45219955647193
O	1.93912780330202	1.54969131144166	3.26528238998988
O	3.99538962886231	0.04084504384078	1.48435410208227
O	1.93915602018141	1.54966595568733	-3.26543712324394
O	4.01400718893816	-0.02847857302339	-1.38180586524776
O	-1.54979806083017	-1.93897613034190	-3.26529423468508
O	0.28448834911434	-3.75210646172621	-1.45230408642316
O	0.24628493864363	3.52185092649688	-1.79417352528327
O	0.24632645290580	3.52186840028995	1.79411254235630
H	-1.24063549573741	-1.02710630914532	3.09117541830757
H	0.49537713568950	-4.52451936412066	1.99437798310728
H	-1.13491186430461	-1.06715051590443	-3.12072498277693
H	0.92386398718337	-3.95714384002356	-2.14651188188441
H	4.14078730930087	-0.71813759463177	-2.04593549376912
H	4.76170980732494	-0.08706708466983	2.06042473859721
H	1.95477631558301	2.05620493965890	-4.08317823963514

H	1.96146032643577	2.10922799800266	4.04885722491674
H	-0.14969863773863	4.25256086234680	2.28138900042035
H	1.04689091274929	1.17133367107632	-3.15509539938833
H	1.00818199917279	1.28971449469768	3.12245078666846
H	3.41027042901454	-0.72918343479433	1.61605588082253
H	4.32189803087411	-0.38355782883476	-0.53794047680211
H	-2.23304735826799	-1.87787529019096	3.94137973108906
H	-2.20392630759147	-1.83823129655049	-3.96394905642954
H	1.00264618546713	-3.11441550380892	1.61283656471671
H	0.71466019193392	-3.95865495748308	-0.61177381708526
H	-0.40718863300475	2.79068566735900	1.77939728571733
H	-0.14517035667279	4.23574614551259	-2.30932491619587
H	-0.35120218414884	2.74926978031636	-1.85898525715227
H	-2.78559091470661	0.40311592098861	1.78178142433224
H	-4.25950382096875	0.16915937332122	2.25453459042246
H	-2.74357907841244	0.34671974852079	-1.85317216670706
H	-4.24249958082499	0.17103454074589	-2.27862720366051

Table S16: DFT optimized coordinates of Model II - $[NpO_2(O_2)_3]^{4-}$

Np	0.17631098841023	-0.08064561942046	-0.00618656592472
O	1.41386732376851	1.28717865616069	-0.04169396429194
O	-1.09429580966690	-1.41861789961528	0.00401009393054
O	-0.15496596442376	0.18929457664285	2.31204461738540
O	-1.07705481563474	1.07810630440679	1.59717145239991
O	1.83517492735644	-1.58391013302540	-0.69503143563175
O	1.80406167570876	-1.56773170444009	0.76476732888598
O	-0.15433900739818	0.17816096371136	-2.32704869488943
O	-1.07554107408741	1.07315239427465	-1.61571310940609
Li	-2.27445856952896	-2.93511297171699	0.10181891597313
Li	2.80464583230314	2.61673599085678	-0.36868291168596
O	-3.52186762197209	-0.24607712103465	-1.79420150929122
O	-3.52185898365908	-0.24605940362980	1.79408873056867
O	-1.54980586524548	-1.93895970958684	3.26530123200206
O	0.28448758677912	-3.75210647100967	1.45220259648139
O	1.93913249296192	1.54968097465733	3.26528640301890
O	3.99538684229538	0.04084825623657	1.48435831823544
O	1.93915100928966	1.54967498459783	-3.26543453498361
O	4.01401299360040	-0.02848138649858	-1.38179335155449
O	-1.54980765773958	-1.93896988945786	-3.26529517848258
O	0.28448625841341	-3.75210720774021	-1.45229993820390
O	0.24628093027520	3.52185354415571	-1.79416627161105
O	0.24633138976958	3.52186490794679	1.79412009946978
H	-1.02735188924998	-1.14582775193356	3.00693760923867

H	0.70922943465212	-4.40124526700331	2.02205065734822
H	-1.03160636711057	-1.14461065477521	-3.00506198526606
H	0.76549602213554	-4.49087225953418	-1.84090265510058
H	4.59579893686363	-0.41303008145966	-2.04967291414710
H	4.63197394839350	-0.10664564887818	0.77185575795401
H	1.64305625072459	2.36117992331598	-3.69275417772007
H	1.66264899420279	2.35792869920777	3.71127030411652
H	-0.28266026362763	4.21142489239777	2.21069927069503
H	1.11711457632537	1.07267934245875	-3.00514348770608
H	1.10777729038427	1.08329566844385	3.02171348415205
H	3.30570124361010	-0.64682639540324	1.33820250519184
H	3.31138695721146	-0.70139188116183	-1.19365336509380
H	-2.36897244529537	-1.58600364667327	3.63189453619627
H	-2.36840691006409	-1.59289593228615	-3.63994862669816
H	0.97154881949299	-3.08764046303094	1.23241998034503
H	0.95074095823614	-3.09252107837844	-1.17692694673700
H	-0.31091982861963	2.70806164017191	1.78922324668399
H	-0.27899743189772	4.20707775793162	-2.22300598064751
H	-0.31329273323935	2.70954472615064	-1.78549114078441
H	-2.70184809075327	0.30448100083433	1.78408086819491
H	-4.21155918991528	0.29797201835546	2.19070051629713
H	-2.70573388641149	0.30920026817311	-1.78535960460930
H	-4.20991060756410	0.28818402144544	-2.20711785319847

Table S17: DFT optimized coordinates of Model II - $[NpO_2(O_2)_2(O_2)^{\bullet}]^{3-}$

Np	-0.00550411429777	0.03560872081758	0.00731641328950
O	1.29109721211688	1.32772045478378	-0.01569273151363
O	-1.23534746412468	-1.32096767410880	0.00033475644679
O	-0.38428373322211	0.40027382251197	2.22517011243171
O	-1.29113437650708	1.28144911281885	1.50657256298830
O	1.81520318933234	-1.62056763147734	-0.26640918485656
O	1.67092256320565	-1.43591173139087	1.03291397650657
O	-0.24757761401258	0.25654829862505	-2.26534473541313
O	-1.19255948048552	1.17944880914977	-1.64698353806441
Li	-2.51040959245412	-2.91014576746726	-0.20901219970514
Li	2.72776985305723	2.69510678209403	-0.13966066227244
O	-3.52186807291127	-0.24607632577857	-1.79420040489455
O	-3.52185536566317	-0.24606477822238	1.79409837741878
O	-1.54979121574529	-1.93897046648163	3.26529920023310
O	0.28448682878474	-3.75210705565529	1.45219947271016
O	1.93912780622940	1.54969128876340	3.26528240788368
O	3.99538955642516	0.04084513857619	1.48435420070521

O	1.93915602770553	1.54966593458496	-3.26543713440753
O	4.01400719217194	-0.02847857440861	-1.38180587018624
O	-1.54979803181949	-1.93897614091326	-3.26529424426390
O	0.28448835360237	-3.75210646010562	-1.45230408754472
O	0.24628490648939	3.52185095112865	-1.79417349008960
O	0.24632642764157	3.52186842463539	1.79411249435778
H	-1.24063575597392	-1.02710625927834	3.09117525415668
H	0.49537712537671	-4.52451936686065	1.99437798767607
H	-1.13491190514783	-1.06715050399706	-3.12072496472994
H	0.92386398702844	-3.95714383984649	-2.14651188182657
H	4.14078730511858	-0.71813759410990	-2.04593549252367
H	4.76170980710013	-0.08706710031864	2.06042473782625
H	1.95477631668230	2.05620493643790	-4.08317824208199
H	1.96146032630395	2.10922799997720	4.04885722322294
H	-0.14969863874849	4.25256086296069	2.28138899481871
H	1.04689091097449	1.17133369942916	-3.15509538439056
H	1.00818201791994	1.28971452123115	3.12245076648095
H	3.41027053606951	-0.72918353959457	1.61605571701426
H	4.32189803087188	-0.38355783973277	-0.53794046767721
H	-2.23304735357550	-1.87787528869900	3.94137974030214
H	-2.20392630459057	-1.83823129572267	-3.96394906087829
H	1.00264608987103	-3.11441541290221	1.61283670260096
H	0.71466017256529	-3.95865496583713	-0.61177381588065
H	-0.40718859552453	2.79068562437597	1.77939735839211
H	-0.14517035968856	4.23574614742294	-2.30932491172580
H	-0.35120212739916	2.74926974319767	-1.85898532091014
H	-2.78559085809895	0.40311589293058	1.78178148492610
H	-4.25950382184405	0.16915937537901	2.25453458565772
H	-2.74357910294984	0.34671978820016	-1.85317212011510
H	-4.24249957954571	0.17103453934743	-2.27862720389329

Table S18: DFT optimized coordinates of Model III - $[UO_2(O_2)_3]^{4-}$

U	0.19614303570786	-0.13084887553114	0.00074897730904
O	1.51613122282389	1.28675525113822	-0.01171060303148
O	-1.13790348315650	-1.52721179192742	-0.00899290405388
O	-0.12461552121489	0.15307445431192	2.27622504974323
O	-1.04688918117883	1.04586787263440	1.56844969912509
O	1.83411791729680	-1.59683485697267	-0.69295690201637
O	1.80506159622042	-1.58345911927191	0.76864716526264
O	-0.11770407981198	0.14185510139370	-2.28387098000506
O	-1.03888373228226	1.03912552651887	-1.57824336759833
Li	2.65979804080921	2.25278443845422	1.52089961901896

Li	-2.10438599843432	-2.81491675305710	-1.60977490145455
Li	2.64439173229193	2.23745453450148	-1.56917704938652
Li	-2.10435765662267	-2.81494856922642	1.46529383703885
O	-3.52182761122684	-0.24612685808731	-1.79429170131035
O	-3.52179278818974	-0.24614967289861	1.79423589163423
O	-1.54980021465940	-1.93896669116054	3.26529718048120
O	0.28442411764409	-3.75237132533692	1.45122111466161
O	1.93911996009887	1.54970286959975	3.26528285808234
O	3.99474687774328	0.04135802922819	1.48526715917895
O	1.93914470176902	1.54967690342949	-3.26544402658937
O	4.01335485703827	-0.02818850101068	-1.38326963256530
O	-1.54981406289295	-1.93896709654685	-3.26530010817368
O	0.28409310376611	-3.75234424272791	-1.45082327312109
O	0.24628975649385	3.52184624069669	-1.79415912111310
O	0.24634965351282	3.52184819775051	1.79413909849424
H	-1.02738253262184	-1.14581685966110	3.00694622437645
H	0.70926662583790	-4.40129140063754	2.02199578026240
H	-1.03162054190371	-1.14461082202319	-3.00506372831167
H	0.76553072988755	-4.49092355749842	-1.84082241409362
H	4.59577957529213	-0.41300678765076	-2.04970153339446
H	4.63203667549865	-0.10677887627462	0.77189085945986
H	1.64305801056626	2.36118061295144	-3.69275838687625
H	1.66265093748076	2.35793031410320	3.71127367048433
H	-0.28266364393253	4.21142548408091	2.21069938529464
H	1.11708746679350	1.07267195588697	-3.00512490694230
H	1.10776890391628	1.08326562830774	3.02171071199531
H	3.30647240919874	-0.64750364401620	1.33673585874995
H	3.31221443797798	-0.70185237861694	-1.19131605085170
H	-2.36897771981468	-1.58600248700970	3.63190001563554
H	-2.36840408167012	-1.59290380931878	-3.63995383987411
H	0.97143297126877	-3.08712029464008	1.23408026296846
H	0.95111881761754	-3.09206632353293	-1.17939197413871
H	-0.31093119083767	2.70809156359203	1.78922874860459
H	-0.27900074879052	4.20707746819078	-2.22300734563730
H	-0.31329729506185	2.70956084363449	-1.78551660901731
H	-2.70195483246463	0.30462111891966	1.78388012185014
H	-4.21155742266972	0.29797280773775	2.19070862534105
H	-2.70581408292129	0.30927882222683	-1.78522777708743
H	-4.20990880336065	0.28818528824056	-2.20712552112113

Table S19: DFT optimized coordinates of Model III - $[UO_2(O_2)_2(O_2)^{\bullet}]^{3-}$

U	0.09970186028190	-0.03036395440271	0.01832205253235
O	1.53001775775245	1.26228646586795	0.01164293454849
O	-1.16719367576934	-1.49319588736392	0.03455259795496
O	-0.27738075645746	0.28652014853912	2.23469138331043
O	-1.15872758310462	1.15405844091804	1.47064650649273
O	1.91709295195255	-1.69481429753773	-0.27779297549474
O	1.76859797279345	-1.50891823848244	1.02118207574999
O	-0.14438315626129	0.16442631724619	-2.25323383053611
O	-1.06431792526156	1.06736904393319	-1.57248744497505
Li	2.78909021376516	2.21640948661367	1.43976999557068
Li	-2.11751995069201	-2.64939096292275	-1.52388014214749
Li	2.78914344444792	2.21640971871927	-1.63527788578344
Li	-2.11752261248522	-2.64939087124727	1.55121755954873
O	-3.52186767612379	-0.24607673200556	-1.79419893153797
O	-3.52185491326139	-0.24606526292642	1.79409925454904
O	-1.54979184990501	-1.93897026491443	3.26529896400350
O	0.28448680388030	-3.75210695398463	1.45219956471136
O	1.93912786825028	1.54969138927135	3.26528220170896
O	3.99538988455688	0.04084463386472	1.48435374824894
O	1.93915612444980	1.54966583042294	-3.26543708638268
O	4.01400724882834	-0.02847851789755	-1.38180582133826
O	-1.54979915948516	-1.93897565558041	-3.26529385368522
O	0.28448822411676	-3.75210663302999	-1.45230406606061
O	0.24628546029097	3.52185062744233	-1.79417458399210
O	0.24632595682696	3.52186886773042	1.79411135072908
H	-1.24063492906122	-1.02710641726094	3.09117572896130
H	0.49537706750669	-4.52451938808380	1.99437799525292
H	-1.13491001614771	-1.06715120303247	-3.12072568295952
H	0.92386400272824	-3.95714383461595	-2.14651188228291
H	4.14078730770451	-0.71813761021021	-2.04593549813325
H	4.76170982482090	-0.08706698749205	2.06042475905516
H	1.95477629073083	2.05620495413811	-4.08317825026062
H	1.96146029611301	2.10922800869860	4.04885724031674
H	-0.14969867075994	4.25256089068946	2.28138895015137
H	1.04689067754933	1.17133375718394	-3.15509538290199
H	1.00818187084946	1.28971437008733	3.12245095876440
H	3.41027009470582	-0.72918309942408	1.61605633986490
H	4.32189797925205	-0.38355783313519	-0.53794054310836
H	-2.23304737945501	-1.87787529954861	3.94137966166801
H	-2.20392636459521	-1.83823132300084	-3.96394896812378
H	1.00264621326199	-3.11441560475132	1.61283648640970
H	0.71466031213085	-3.95865483058098	-0.61177385776181

H	-0.40718782119394	2.79068488506400	1.77939917462256
H	-0.14517035794108	4.23574613570222	-2.30932500316599
H	-0.35120293905057	2.74927019711108	-1.85898335563942
H	-2.78559165952531	0.40311667680349	1.78177988395545
H	-4.25950379102609	0.16915935281959	2.25453467210880
H	-2.74357964265031	0.34672021960784	-1.85317432784751
H	-4.24249960752121	0.17103463041079	-2.27862720086644

Table S20: DFT optimized coordinates of Model III - $[NpO_2(O_2)_3]^{4-}$

Np	0.20339402323012	-0.11918546936392	0.01704928380487
O	1.49603101910987	1.27786815091198	0.02031627792740
O	-1.09074143681526	-1.50775487914407	0.01227861528555
O	-0.11511480773016	0.14877966161626	2.32903043025740
O	-1.02738164296414	1.03086733902919	1.59782190340418
O	1.83276188726312	-1.59531750044988	-0.68030199579220
O	1.80754382833742	-1.58583919230297	0.77074941347807
O	-0.10665811036740	0.12976214459806	-2.29672484002725
O	-1.01792124732357	1.01999113297820	-1.57469725752663
Li	2.68422854436578	2.30086856412635	1.67541258140312
Li	-2.00841202696798	-2.87184145686386	-1.53367153947543
Li	2.68428247569457	2.30086912122041	-1.39963526359430
Li	-2.00841732403084	-2.87184120261814	1.54142593648870
O	-3.52186755473178	-0.24607686526407	-1.79419954109601
O	-3.52185482746200	-0.24606533047415	1.79409956350189
O	-1.54979129560936	-1.93897022513676	3.26529899009883
O	0.28448692450983	-3.75210705590242	1.45219975217055
O	1.93912796271191	1.54969156984060	3.26528221884683
O	3.99538999292092	0.04084496196721	1.48435392414475
O	1.93915593038058	1.54966588592040	-3.26543713059928
O	4.01400720921945	-0.02847837741574	-1.38180623378670
O	-1.54979972029658	-1.93897560761188	-3.26529390141853
O	0.28448792154066	-3.75210674928807	-1.45230422954343
O	0.24628573641522	3.52185032404930	-1.79417545973507
O	0.24632608022519	3.52186877040063	1.79411172263132
H	-1.02737353942148	-1.14581137060509	3.00693893880781
H	0.70922964499502	-4.40124512216637	2.02205087934714
H	-1.03161738354378	-1.14460558635915	-3.00506260708013
H	0.76549596044750	-4.49087270506874	-1.84090239474428
H	4.59579863246996	-0.41303018392001	-2.04967282861909
H	4.63197252502540	-0.10664375299377	0.77185578800773
H	1.64305531379177	2.36118058361324	-3.69275474557249
H	1.66264901876935	2.35792898179209	3.71126969237619

H	-0.28266053036712	4.21142504447762	2.21069884035488
H	1.11711088071349	1.07268987567842	-3.00513947213292
H	1.10778654757891	1.08327788670024	3.02171985666392
H	3.30569725241614	-0.64682508981605	1.33820715030365
H	3.31139773929858	-0.70139581789932	-1.19363745574887
H	-2.36897232650350	-1.58600323700911	3.63189514547794
H	-2.36840675059709	-1.59289509148172	-3.63994986247426
H	0.97155110466016	-3.08764029768823	1.23242143916255
H	0.95073629564577	-3.09252023624673	-1.17692430745356
H	-0.31091106583698	2.70805525655808	1.78923711562011
H	-0.27899748760401	4.20707776499191	-2.22300605683573
H	-0.31329937441999	2.70954896018459	-1.78547759392745
H	-2.70185389248890	0.30449052388363	1.78406252591915
H	-4.21155903795843	0.29797165615294	2.19070120810162
H	-2.70573179590280	0.30919749943050	-1.78536531005615
H	-4.20991072611192	0.28818414224015	-2.20711785592213

Table S21: DFT optimized coordinates of Model III - $[NpO_2(O_2)_2(O_2)^{\bullet}]^{3-}$

Np	0.07042034587663	-0.05499336600262	0.02194324272930
O	1.43006229995604	1.25411241186606	0.00979136382466
O	-1.17824266837954	-1.46306096209666	0.03741320457948
O	-0.31761631850311	0.33165095825138	2.20656753309292
O	-1.20789147554245	1.19898748849720	1.46591910971281
O	1.87825664121961	-1.68079870825294	-0.24761964235057
O	1.73273105025026	-1.49101690702401	1.04855852213721
O	-0.18711104992028	0.19814199190872	-2.21491470471228
O	-1.11802673068647	1.10701814153824	-1.57027409517155
Li	2.62169359086807	2.07974171402464	1.51384775049917
Li	-2.04077303508773	-2.79305435149724	-1.47093433655377
Li	2.62174691542334	2.07974184852903	-1.56120016825379
Li	-2.04077607425950	-2.79305397713545	1.60416334063712
O	-3.52186783910185	-0.24607648215797	-1.79419863923904
O	-3.52185506907220	-0.24606508710516	1.79409885809284
O	-1.54979175135068	-1.93897032195173	3.26529895389003
O	0.28448661800643	-3.75210682325048	1.45219979531747
O	1.93912785396408	1.54969139038394	3.26528219779096
O	3.99539002954811	0.04084453069220	1.48435345055291
O	1.93915609593105	1.54966589141917	-3.26543709739546
O	4.01400723195630	-0.02847852499196	-1.38180581400767
O	-1.54979930625502	-1.93897559895446	-3.26529386968233
O	0.28448822305619	-3.75210657774484	-1.45230406167958
O	0.24628546179271	3.52185062164184	-1.79417459419645

O	0.24632587279869	3.52186894784518	1.79411111363220
H	-1.24063492548821	-1.02710637263685	3.09117570854411
H	0.49537705715161	-4.52451938788498	1.99437800470563
H	-1.13490995863463	-1.06715117825836	-3.12072565006665
H	0.92386399572099	-3.95714385539261	-2.14651188755769
H	4.14078731510947	-0.71813760370564	-2.04593550475474
H	4.76170983049556	-0.08706701027748	2.06042476801773
H	1.95477630174957	2.05620494850104	-4.08317824564246
H	1.96146031262768	2.10922800155537	4.04885724169434
H	-0.14969868010897	4.25256089984733	2.28138894756391
H	1.04689065010084	1.17133373687008	-3.15509536077775
H	1.00818186238747	1.28971435665907	3.12245095816384
H	3.41026984105257	-0.72918292123892	1.61605668609549
H	4.32189798584970	-0.38355782694966	-0.53794054007854
H	-2.23304737859122	-1.87787532026476	3.94137966864126
H	-2.20392635918443	-1.83823131597324	-3.96394895784047
H	1.00264650262830	-3.11441578877351	1.61283619334022
H	0.71466031326536	-3.95865483791514	-0.61177386756851
H	-0.40718773512798	2.79068480737653	1.77939941943864
H	-0.14517033906379	4.23574612889253	-2.30932501156781
H	-0.35120297315321	2.74927026395118	-1.85898327195831
H	-2.78559148654613	0.40311646964712	1.78178035598992
H	-4.25950379346961	0.16915936010746	2.25453465744860
H	-2.74357943320220	0.34671985843846	-1.85317474934909
H	-4.24249961316020	0.17103463053468	-2.27862717784264

Table S22: DFT optimized coordinates of Model IV - $[UO_2(O_2)_3]^{4-}$

U	0.14199753529222	-0.09580666360149	0.00656249305660
O	1.55162000556005	1.36992813191114	-0.00258499273872
O	-1.28264580641366	-1.54963409481443	0.00100065505154
O	-0.13628123257524	0.15639628422706	2.22512808808972
O	-1.05926675995678	1.05548913998401	1.54565921597585
O	1.75705163896212	-1.51915404630317	-0.68783154396073
O	1.73020230969253	-1.50618230339269	0.76834692944337
O	-0.12386376919644	0.14325418545624	-2.21747712481180
O	-1.04821012350640	1.04719495365104	-1.54512323921861
Li	2.95777169083294	1.51991376710769	1.53870044611477
Li	-1.50610262680614	-2.97218648850075	-1.53759538295424
Li	2.95782558266400	1.51991417515006	-1.53634731080219
Li	-1.50610757433407	-2.97218619560253	1.53750191204955
Li	-3.52188249150397	-1.35956881096294	0.00000288874059
Li	1.34364602518296	3.53608677683772	0.00126388030045

O	-3.52186771201794	-0.24607678132349	-1.79419905874114
O	-3.52185478723833	-0.24606518709904	1.79409928273257
O	-1.54979139315176	-1.93897018098634	3.26529899655784
O	0.28448690085392	-3.75210709507598	1.45219976395778
O	1.93912785090982	1.54969152534737	3.26528224617473
O	3.99539017143552	0.04084481553313	1.48435372733795
O	1.93915602057075	1.54966601222415	-3.26543716568212
O	4.01400722606972	-0.02847843759918	-1.38180591854076
O	-1.54979966191475	-1.93897562741783	-3.26529390270728
O	0.28448793088159	-3.75210677694326	-1.45230423746742
O	0.24628547981636	3.52185060460011	-1.79417461705859
O	0.24632589173541	3.52186875244670	1.79411133113220
H	-1.02737354822550	-1.14581144812075	3.00693895371418
H	0.70922965139740	-4.40124512222777	2.02205087572599
H	-1.03161734844453	-1.14460552246659	-3.00506256986010
H	0.76549595334834	-4.49087270695766	-1.84090240501759
H	4.59579864509021	-0.41303021363809	-2.04967281543206
H	4.63197249718763	-0.10664366422825	0.77185580051181
H	1.64305530560616	2.36118057684902	-3.69275473586896
H	1.66264900248269	2.35792900169587	3.71126969528795
H	-0.28266052669919	4.21142505757803	2.21069882578469
H	1.11711075163722	1.07268977902819	-3.00513941454408
H	1.10778665025401	1.08327795313589	3.02171988326930
H	3.30569702228859	-0.64682495181668	1.33820744490695
H	3.31139755641755	-0.70139566770239	-1.19363793917006
H	-2.36897232405250	-1.58600321464732	3.63189513767610
H	-2.36840675606485	-1.59289510318170	-3.63994986087065
H	0.97155109808428	-3.08764027847927	1.23242143700072
H	0.95073631591404	-3.09252017537140	-1.17692427943279
H	-0.31091074930116	2.70805523473971	1.78923765986337
H	-0.27899752099216	4.20707775532696	-2.22300602342075
H	-0.31329897009859	2.70954859945913	-1.78547863904892
H	-2.70185396376224	0.30449035218217	1.78406288687100
H	-4.21155904717270	0.29797169070118	2.19070116471746
H	-2.70573160243632	0.30919737940398	-1.78536589268553
H	-4.20991072304608	0.28818417948528	-2.20711782110467

Table S23: DFT optimized coordinates of Model IV - $[UO_2(O_2)_2(O_2)^{\bullet}]^{3-}$

U	0.02550651800358	-0.00474030663181	0.00948457444399
O	1.48893887800073	1.35326134670337	0.01159021322848
O	-1.30719839223067	-1.49558529955391	0.02159615736535
O	-0.30330213135969	0.31857833829469	2.17622398153326

O	-1.19605435910371	1.19184511141933	1.44360137376342
O	1.80788543536303	-1.60721691824575	-0.35552952000578
O	1.68409121941860	-1.44407097021810	0.94315520460069
O	-0.18101880983149	0.19158043701888	-2.19655042332935
O	-1.10824598577954	1.10299130658442	-1.55367411199641
Li	2.95777209224720	1.51991434738918	1.53870036453788
Li	-1.50610365001611	-2.97218691847870	-1.53759564175318
Li	2.95782562418743	1.51991432749674	-1.53634730685655
Li	-1.50610665339778	-2.97218645739451	1.53750241827933
Li	-3.52188260783231	-1.35956841760016	0.00000273701989
Li	1.34364587637507	3.53608638063169	0.00126408781201
O	-3.52186769124087	-0.24607681782602	-1.79419900788211
O	-3.52185492429515	-0.24606526971372	1.79409924419613
O	-1.54979175741612	-1.93897035704921	3.26529893086729
O	0.28448679459259	-3.75210700048454	1.45219954669606
O	1.93912787184624	1.54969141213553	3.26528226149303
O	3.99538989695199	0.04084461575328	1.48435374906781
O	1.93915608888009	1.54966598445492	-3.26543715325377
O	4.01400718348652	-0.02847852011275	-1.38180587465919
O	-1.54979931248750	-1.93897562022391	-3.26529383961703
O	0.28448823006692	-3.75210655106981	-1.45230404211702
O	0.24628548722162	3.52185062698186	-1.79417463147549
O	0.24632591059822	3.52186892506944	1.79411121453826
H	-1.24063490925945	-1.02710634592927	3.09117569614581
H	0.49537705213970	-4.52451939955703	1.99437799393282
H	-1.13490994009464	-1.06715115375539	-3.12072566636936
H	0.92386399517341	-3.95714384961094	-2.14651188544135
H	4.14078730234222	-0.71813761131144	-2.04593548210456
H	4.76170981232549	-0.08706700731502	2.06042475002945
H	1.95477634009488	2.05620493636018	-4.08317824934600
H	1.96146035750206	2.10922800752985	4.04885726243528
H	-0.14969865999372	4.25256089686140	2.28138894837889
H	1.04689053673195	1.17133364109283	-3.15509529458156
H	1.00818174613144	1.28971428283606	3.12245085883538
H	3.41027009971657	-0.72918306452225	1.61605636294965
H	4.32189800427168	-0.38355781961570	-0.53794054346872
H	-2.23304739005075	-1.87787531776270	3.94137966528051
H	-2.20392634700495	-1.83823132407002	-3.96394896115278
H	1.00264626603592	-3.11441551766349	1.61283645349305
H	0.71466031472191	-3.95865484879454	-0.61177384714477
H	-0.40718776755901	2.79068478693478	1.77939931916129
H	-0.14517035232083	4.23574612109726	-2.30932501449342
H	-0.35120294706840	2.74927021647116	-1.85898331115671

H	-2.78559159198669	0.40311673115346	1.78177983190767
H	-4.25950379651566	0.16915933253188	2.25453466772627
H	-2.74357954063963	0.34672032886581	-1.85317422745474
H	-4.24249961555881	0.17103461695294	-2.27862720659974

Table S24: DFT optimized coordinates of Model IV - $[NpO_2(O_2)_3]^{4-}$

Np	0.13044437175069	-0.08260009563147	0.00672526118829
O	1.52172421570418	1.35848978719199	-0.00155963310990
O	-1.26664506610345	-1.51969305848428	0.00184700690816
O	-0.11847329622192	0.13845187081482	2.27124524497275
O	-1.03226680589970	1.02943029213561	1.57456913348306
O	1.74458815694052	-1.51173711531726	-0.68295170891205
O	1.71833938760158	-1.49805583186209	0.75869631402151
O	-0.10520468836439	0.12544832782666	-2.26204937149179
O	-1.02107242916809	1.02115671392211	-1.57297122854534
Li	2.95777176133476	1.51991396966269	1.53870021596082
Li	-1.50610269715840	-2.97218648735264	-1.53759528761252
Li	2.95782547650626	1.51991441794360	-1.53634700292044
Li	-1.50610754321894	-2.97218637506034	1.53750185893231
Li	-3.52188253165850	-1.35956869989885	0.00000284899231
Li	1.34364619843999	3.53608652183132	0.00126383853966
O	-3.52186763353332	-0.24607673093270	-1.79419903586457
O	-3.52185470265617	-0.24606516945856	1.79409928247370
O	-1.54979136301182	-1.93897011649671	3.26529897658201
O	0.28448688934092	-3.75210705404550	1.45219979299396
O	1.93912782019176	1.54969148539035	3.26528224748045
O	3.99539006490539	0.04084472241560	1.48435372316224
O	1.93915593243483	1.54966590899101	-3.26543717243558
O	4.01400725352794	-0.02847844138265	-1.38180592418668
O	-1.54979956857449	-1.93897555451438	-3.26529389237868
O	0.28448795091076	-3.75210676752623	-1.45230417570054
O	0.24628537587749	3.52185054967570	-1.79417458418820
O	0.24632582851266	3.52186868347374	1.79411132804863
H	-1.02737357518124	-1.14581149442466	3.00693898587565
H	0.70922965413986	-4.40124511793995	2.02205087252308
H	-1.03161742649422	-1.14460561633502	-3.00506261627587
H	0.76549598258214	-4.49087266842910	-1.84090244196781
H	4.59579862743903	-0.41303024562412	-2.04967282461705
H	4.63197250227445	-0.10664365258490	0.77185580566780
H	1.64305536646987	2.36118058786192	-3.69275476957685
H	1.66264905088564	2.35792900984177	3.71126972900613
H	-0.28266052764185	4.21142506307470	2.21069884324741

H	1.11711079173669	1.07268981804304	-3.00513943317545
H	1.10778659201190	1.08327792858151	3.02171986131747
H	3.30569713312917	-0.64682488831543	1.33820747912924
H	3.31139762216975	-0.70139563559995	-1.19363795751996
H	-2.36897233363629	-1.58600322430704	3.63189515463819
H	-2.36840676718157	-1.59289511856459	-3.63994987356896
H	0.97155109370548	-3.08764030719051	1.23242143559126
H	0.95073624877641	-3.09252029768599	-1.17692432462156
H	-0.31091067554390	2.70805538758550	1.78923767616525
H	-0.27899748505040	4.20707777384575	-2.22300606153757
H	-0.31329890228868	2.70954869949719	-1.78547865376300
H	-2.70185409004506	0.30449031449368	1.78406288353977
H	-4.21155906274329	0.29797167184883	2.19070118199057
H	-2.70573168592047	0.30919733889665	-1.78536589148226
H	-4.20991075236614	0.28818414390666	-2.20711784315457

Table S25: DFT optimized coordinates of Model IV - $[NpO_2(O_2)_2(O_2)^{\bullet}]^{3-}$

Np	0.04895961787504	-0.02485828182382	0.00563904881438
O	1.47479971855696	1.33115446694119	0.00636325434376
O	-1.27898201283462	-1.47980207207552	0.01719784545064
O	-0.31602005016008	0.33340655519662	2.13853082468177
O	-1.20904300504193	1.20498901519813	1.42768108852935
O	1.82360325959291	-1.62858121976108	-0.28810166814403
O	1.69398627244150	-1.45010065495358	1.00941216164196
O	-0.20982070682460	0.21891283098832	-2.16477785821210
O	-1.13638628869896	1.12974643734052	-1.53971991162371
Li	2.95777221193001	1.51991447869974	1.53870056237366
Li	-1.50610352822089	-2.97218673846453	-1.53759543272848
Li	2.95782552809749	1.51991423717359	-1.53634746356542
Li	-1.50610690541376	-2.97218634091444	1.53750218069946
Li	-3.52188239013022	-1.35956864910632	0.00000274239269
Li	1.34364594228026	3.53608644058245	0.00126407709279
O	-3.52186773709869	-0.24607676477279	-1.79419899425458
O	-3.52185501386508	-0.24606528709393	1.79409925307809
O	-1.54979169124349	-1.93897029839908	3.26529893217035
O	0.28448679073567	-3.75210707510246	1.45219962701305
O	1.93912780722266	1.54969136342452	3.26528219184069
O	3.99538996214271	0.04084463451225	1.48435379828833
O	1.93915604205061	1.54966594204375	-3.26543710735436
O	4.01400720121871	-0.02847849996837	-1.38180584360490
O	-1.54979932445581	-1.93897561687623	-3.26529386759041
O	0.28448821408062	-3.75210657905877	-1.45230406332354

O	0.24628548231763	3.52185070234698	-1.79417464372224
O	0.24632595647884	3.52186897403574	1.79411123272325
H	-1.24063498102905	-1.02710643791437	3.09117574563550
H	0.49537705347922	-4.52451940460163	1.99437798372789
H	-1.13490999129617	-1.06715124270749	-3.12072569739126
H	0.92386398761058	-3.95714386710578	-2.14651188351072
H	4.14078732079615	-0.71813759779060	-2.04593549836719
H	4.76170982889280	-0.08706696868529	2.06042475360455
H	1.95477632360996	2.05620493803531	-4.08317824405764
H	1.96146035225048	2.10922796964376	4.04885724916089
H	-0.14969868280481	4.25256087850606	2.28138893015946
H	1.04689065411304	1.17133371842326	-3.15509536410263
H	1.00818184970140	1.28971436676072	3.12245094438137
H	3.41026994437023	-0.72918317681020	1.61605628469307
H	4.32189798662092	-0.38355783653591	-0.53794053197051
H	-2.23304736503452	-1.87787531358909	3.94137966980640
H	-2.20392636309111	-1.83823127924368	-3.96394894834665
H	1.00264628997137	-3.11441546478851	1.61283641387761
H	0.71466031384042	-3.95865483701455	-0.61177384474891
H	-0.40718779433942	2.79068475251681	1.77939933272390
H	-0.14517035328607	4.23574613150704	-2.30932500327966
H	-0.35120300323177	2.74927007785580	-1.85898331606722
H	-2.78559156126175	0.40311672590849	1.78177983715187
H	-4.25950378206529	0.16915936534021	2.25453466460074
H	-2.74357953792199	0.34672029108366	-1.85317424938106
H	-4.24249960911915	0.17103462746658	-2.27862721609718

Table S26: DFT optimized coordinates of Model V - $[UO_2OH_4]^{2-}$

U	8.60269517574991	17.45463514341392	8.57584670130582
O	8.52388135331581	15.63492119285798	8.83010955474305
O	8.68163838395474	19.27432675491009	8.32143700939153
O	7.03026837346280	17.26416285576189	6.93431142508405
O	10.17631612932830	17.64708907201321	10.21633871533031
O	6.95269771032056	17.77574227673632	10.11901431143413
O	10.25414226751926	17.13515163453461	7.03360012653420
H	6.62611051302605	16.96982640739073	10.53358542426995
H	10.58241607392446	17.94228087626496	6.62266455130190
H	10.42611274493118	16.81889396685119	10.64011461812766
H	6.78354434358047	18.09361845246051	6.51113490415330

Table S27: DFT optimized coordinates of Model V - $[UO_2OH_4]^-$

U	8.60230825095608	17.45218548180237	8.57424912728676
O	8.52883438899886	15.67599422843264	8.97729187598163
O	8.67627730469433	19.22893058852558	8.17440672778401
O	7.11264667340814	17.30385284881093	6.94976741971662
O	10.09234930934180	17.60764834574665	10.19801146468956
O	7.02638854499303	17.71357833499514	10.09948777650725
O	10.17995572713502	17.19720812191357	7.04961627274118
H	6.66781978848775	16.97078638910095	10.59971123466131
H	10.54672574283848	17.94502199239783	6.56286782465202
H	10.36804209413327	16.84174698204022	10.71568702529105
H	6.83847524412666	18.07369531942908	6.43706059236421

Table S28: DFT optimized coordinates of Model V - $[NpO_2OH_4]^{2-}$

Np	8.60555096436296	17.45879646953304	8.57685678571642
O	8.52598278558959	15.66408069257726	8.84043816831440
O	8.68444926343061	19.25367460364611	8.31328145173043
O	7.03918551548938	17.25537081527073	6.95025795341951
O	10.16992056553572	17.65371110467528	10.20559198875284
O	6.96660094345949	17.78159773810421	10.11026185532448
O	10.24158131778252	17.12916264486911	7.04241407106430
H	6.65155672717684	16.95907108341412	10.50126435359238
H	10.55344195107518	17.94866709682580	6.64286401043558
H	10.39257158195221	16.81178587686983	10.61801619392922
H	6.80898145325903	18.09473050740979	6.53691050939629

Table S29: DFT optimized coordinates of Model V - $[NpO_2OH_4]^-$

Np	8.60364252568811	17.45764137647084	8.57713378984564
O	8.52959692459867	15.70563432979782	8.97536495827802
O	8.67778775392096	19.20938534818298	8.17738530891108
O	7.12383690721864	17.29063392779653	6.96449173683545
O	10.08363121320916	17.62033322786006	10.19004981892640
O	7.03755525659050	17.72683566853971	10.09069769964567
O	10.16920467488698	17.18396767003985	7.06378708445456
H	6.68504054246463	16.96142906032784	10.56126346744382
H	10.52102141229017	17.94663200478298	6.58836994742074
H	10.34890483331180	16.83340646648690	10.68188430816838
H	6.85960102493372	18.07474955290923	6.46772922174578

Table S30: DFT optimized coordinates of Model VI - $[UO_2OH_4]^{2-}$

U	8.72341767402755	17.45214664201184	8.72336666851074
O	8.75623879297886	15.67842743251820	8.79164675058867
O	8.68984877786672	19.22541236534647	8.65380981978256
O	7.62904898056640	17.40194155446862	6.70447156088766
O	9.81904129864755	17.49424058687845	10.74493143629245
O	6.71027230042187	17.45037215749590	9.81761332803499
O	10.74395147545883	17.45695532575608	7.63785568159921
H	5.87403196254878	17.35148988363932	10.30554001200661
H	11.57794491794727	17.54397066230610	7.14302256188278
H	10.32049495643497	17.66725806264541	11.55906756675525
H	7.13094947511386	17.27438379008629	5.87877497857092
Li	10.63231436392022	15.44119993349048	6.82591058955664
Li	6.82589802226864	15.44120010755217	6.82020587950589
Li	10.63230067937833	15.44120358491843	10.62660430410995
Li	6.82590219500736	15.44120245589617	10.63228314754874
Li	8.72630648647602	13.72640098603737	8.72630406080793
Li	10.63229814644130	19.46380118102572	10.62660314455152
Li	6.82593691426148	19.46377277294485	10.63234697352586
Li	10.62654966145936	19.46378866164127	6.82014756339580
Li	6.82592323108460	19.46376309157874	6.82016706817898
Li	8.72629104807493	21.17861405058636	8.72630869888058
Li	5.24380073241687	17.45249658767034	5.24379889403118
Li	12.20870107681085	17.45250202451891	12.20870462893246
Li	5.24379776229126	17.45249600299218	12.20869543779335
Li	12.20870033672859	17.45250333488890	5.24379941486221
Li	4.88148791749195	17.45251327894331	8.72629980361451
Li	12.57100668369964	17.45249548779166	8.72630683098868
Li	8.72629551173322	17.45250338853473	12.57099983656124
Li	8.72630254936776	17.45249773135213	4.88149920150876
O	5.46090334531668	15.48039628164819	11.99160054096645
O	7.27398296175181	13.64608264777205	10.17860412267933
O	11.99160739625241	15.48040067873320	5.46090441141869
O	10.17850919164588	13.64610057418464	7.27400644136827
O	11.99160087300888	15.48039988964586	11.99160394863193
O	10.17850174186192	13.64610392542940	10.17850431259549
O	5.46090347931963	15.48039381629900	5.46090387712825
O	7.27400515758977	13.64610207830268	7.27399401007051
O	3.76797834823658	17.45251628319997	6.93210465711056
O	13.68451747989620	17.45249294770668	10.52041097210577
O	3.76800269562822	17.45249901332459	10.52040055334387
O	13.68449887608234	17.45251651497227	6.93209029328307
O	6.93209716320079	17.45249643997132	13.68450217311920

O	10.52040536624548	17.45249909917543	3.76800110869920
O	10.52039720050162	17.45251260271814	13.68450312029675
O	6.93211357267168	17.45248616993050	3.76801862505975
O	5.46088437527269	19.42461759257251	5.46091314020852
O	7.27399760935135	21.25889733480325	7.27399627630838
O	11.99159734790640	19.42460618654539	11.99160090674542
O	10.17849759217614	21.25890148654613	10.17850291724041
O	5.46086563218800	19.42461436010351	11.99156357332959
O	7.27398888313943	21.25889789235553	10.17848967488765
O	11.99162685480535	19.42461169717781	5.46093127326422
O	10.17849244926512	21.25889487327826	7.27400057854682
H	5.54357520408395	15.09727756652876	4.54775667587460
H	4.54554672814921	15.17871856348170	5.68612746339277
H	7.37284987491316	18.32834324311205	3.44364608366078
H	6.72279208144479	17.00572108992430	2.93319234505633
H	3.29762601394616	18.27426285565227	7.20888770354869
H	2.96741922407239	16.91181261931303	6.67984590076826
H	5.78958451453315	19.83864066118145	4.61380502951736
H	4.61854352431821	19.76774783862004	5.84039055177639
H	11.06364392946658	21.65646741921289	7.50088680129006
H	9.99981912553355	21.96769291915863	6.62307937672375
H	12.69277171068948	19.97281026854040	5.89439733961278
H	11.90682655682354	19.83177097237283	4.56362947896843
H	12.83538095401667	15.10383876625043	5.76829904485671
H	11.51266857669682	14.80090733804779	4.82935809012404
H	10.94468467107938	13.18071907019040	7.67557309337712
H	10.06763114532498	13.34917642912986	6.26078287914567
H	7.62421348606158	21.70804438480588	6.45813217563198
H	6.60780809024538	21.97509873411118	7.54538756568677
H	7.36778489651333	21.69659235556469	11.12244719925890
H	6.61302861598834	21.85264590606753	9.71807929958951
H	5.77536591240548	20.10579689354095	12.67684821771802
H	4.58129387027880	19.76634150790255	11.71210068414420
H	12.85133650932280	19.79770782560563	11.69510461387646
H	11.88343728611347	19.71715474053602	12.93170702407347
H	13.95259408118540	16.50822644337537	10.24533941188365
H	14.45814626207580	18.02201429142919	10.68996649752376
H	10.07638586806823	16.57489775307806	14.02819857730536
H	10.74339359354793	17.88657313047835	14.52182916812229
H	12.81309167664556	14.99327623289935	11.81618924569096
H	11.52265262086716	15.19938192315876	12.84637375077467
H	14.12750163400962	16.76220016959699	7.67958112583492
H	14.36879103674225	18.12786147536597	6.89943536529251

H	10.92408629949900	13.07087898440175	9.81975420266055
H	9.90993091969582	13.06293217575961	10.93894437030134
H	7.51452439341021	13.05115560532620	10.91020448780213
H	6.52126723626556	13.12906419281393	9.86399499165129
H	5.65685871308952	15.02071449922082	12.83839589220454
H	4.74163347762748	14.95918026128812	11.55564491192224
H	7.53213359967636	13.04305767079356	6.54537032488187
H	6.79925721844183	12.99215282239536	7.94876833528244
H	3.26097361660084	18.16194535986287	9.84543563957333
H	3.04899725338380	16.77459009107522	10.51832692224837
H	10.65292412952250	16.64716728553514	3.21804224070854
H	10.32269933673788	18.21198219692088	3.17602251558547
H	10.86199052908173	21.77534214870346	9.63411826361922
H	9.81742108691022	21.74712593148282	10.94833566540210
H	7.15365506520551	16.73014633467712	14.30509369990069
H	6.85465859212688	18.34811761918781	14.10173173989652

Table S31: DFT optimized coordinates of Model VI - $[UO_2OH_4]^-$

U	8.72447868368452	17.45208258040592	8.72420269533258
O	8.75409667951215	15.67675234761551	8.77635350500196
O	8.69196824021132	19.22902479946163	8.67106046456738
O	7.62206421289339	17.41348257159927	6.70986391410718
O	9.82770604556296	17.49050992463628	10.73856036207145
O	6.71829161055284	17.45183435481060	9.82474075582077
O	10.73592755886826	17.46141768585243	7.62939143232484
H	5.87665672402673	17.34174544740159	10.30271425347015
H	11.57842732862565	17.54620453563189	7.14646371498145
H	10.31957679945454	17.65773181453470	11.56008508386248
H	7.13078790246116	17.27209399947294	5.88254126805616
Li	10.63230742078333	15.44119924007110	6.82590336294664
Li	6.82589862363986	15.44119997678996	6.82020602625134
Li	10.63230022707414	15.44120313498043	10.62660412530892
Li	6.82590093055068	15.44120337741014	10.63228163738229
Li	8.72630390516592	13.72640097103528	8.72630078740936
Li	10.63229697520721	19.46380064729980	10.62660308584505
Li	6.82593564896818	19.46377388481625	10.63234561513876
Li	10.62659668532459	19.46379813303562	6.82019951349884
Li	6.82592378875054	19.46376322679057	6.82016683951147
Li	8.72627821326385	21.17861136978604	8.72629312153580
Li	5.24380052486614	17.45249618418762	5.24379820087130
Li	12.20870094180411	17.45250276691917	12.20870464430013
Li	5.24379975383419	17.45249619564571	12.20869799885847

Li	12.20870343640201	17.45250399927870	5.24380203558546
Li	4.88149098728138	17.45250825230959	8.72629775455117
Li	12.57100542048576	17.45249407349199	8.72630594200596
Li	8.72629562131891	17.45250359454373	12.57099977332038
Li	8.72630310926454	17.45249840223701	4.88150001070704
O	5.46090285411938	15.48039611289441	11.99160007120685
O	7.27398300101953	13.64608239994400	10.17860473684623
O	11.99161094227838	15.48039983183080	5.46090776598016
O	10.17851086528209	13.64610036520135	7.27400750089389
O	11.99160094806786	15.48039997597026	11.99160383310772
O	10.17850200939741	13.64610399593762	10.17850441517169
O	5.46090327643136	15.48039390095904	5.46090393799690
O	7.27400512181927	13.64610201573793	7.27399412242491
O	3.76797615805131	17.45251960410459	6.93210626979928
O	13.68451719787478	17.45249376443002	10.52041117272632
O	3.76800068846537	17.45249922740446	10.52040012167490
O	13.68449824856006	17.45251683633643	6.93209021223941
O	6.93209686738585	17.45249618449090	13.68450203188958
O	10.52040492267155	17.45249850665427	3.76800032963587
O	10.52039709980126	17.45251241818247	13.68450282106799
O	6.93211378969459	17.45248617993599	3.76801891255437
O	5.46088420449755	19.42461751902743	5.46091343695387
O	7.27399682451436	21.25889859906903	7.27399851693456
O	11.99159728957489	19.42460579116686	11.99160073322738
O	10.17850000950211	21.25890184478324	10.17850240478141
O	5.46086492474818	19.42461424988708	11.99156275097792
O	7.27398938380664	21.25889997421032	10.17849058082425
O	11.99159725679320	19.42460201264607	5.46090019916987
O	10.17848977149909	21.25889553898679	7.27399745471510
H	5.54357519654703	15.09727753581019	4.54775668404301
H	4.54554672377044	15.17871855822918	5.68612745197659
H	7.37284954109699	18.32834316944312	3.44364546169229
H	6.72279206519320	17.00572109325379	2.93319235727051
H	3.29762624122975	18.27426248359645	7.20888750537803
H	2.96741930626061	16.91181246317394	6.67984575983434
H	5.78958449875743	19.83864071149521	4.61380501408832
H	4.61854351236825	19.76774785047305	5.84039051489414
H	11.06364449870789	21.65646606758271	7.50088742329671
H	9.99981904672140	21.96769307056779	6.62307976312301
H	12.69277112248550	19.97281422191674	5.89439737244910
H	11.90682750083677	19.83177080386092	4.56363064802390
H	12.83538101786945	15.10383944403371	5.76829910017336
H	11.51266844236613	14.80090730146940	4.82935799703080

H	10.94468454572350	13.18071929073396	7.67557345403027
H	10.06763124187110	13.34917642538561	6.26078280073244
H	7.62421366479617	21.70804390726045	6.45813186188176
H	6.60780813373053	21.97509865864222	7.54538749206763
H	7.36778563403415	21.69659087565857	11.12244775002864
H	6.61302868446333	21.85264584428706	9.71807918824058
H	5.77536593248551	20.10579686012311	12.67684837875406
H	4.58129397316464	19.76634154113719	11.71210071438377
H	12.85133651312220	19.79770783043675	11.69510464376071
H	11.88343728658002	19.71715475578427	12.93170703683359
H	13.95259425351447	16.50822638352160	10.24533932334461
H	14.45814629180754	18.02201423015833	10.68996649665563
H	10.07638592176240	16.57489781727995	14.02819873280569
H	10.74339360162094	17.88657315968656	14.52182926492413
H	12.81309164973452	14.99327624129201	11.81618926008756
H	11.52265259725754	15.19938191866252	12.84637376675426
H	14.12750169426971	16.76220024012088	7.67958117431213
H	14.36879102171199	18.12786144208459	6.89943533898134
H	10.92408633281180	13.07087903052855	9.81975416299935
H	9.90993093156591	13.06293216292062	10.93894437904425
H	7.51452459851030	13.05115572970061	10.91020445551709
H	6.52126728126527	13.12906422263864	9.86399478174860
H	5.65685878774036	15.02071461124117	12.83839603160673
H	4.74163357255532	14.95918024921164	11.55564495327030
H	7.53213366143255	13.04305768455033	6.54537029310272
H	6.79925722382254	12.99215277327206	7.94876825809665
H	3.26097376475971	18.16194533540001	9.84543575826219
H	3.04899742765223	16.77459013677844	10.51832698566761
H	10.65292420107039	16.64716736162944	3.21804239232347
H	10.32269934970318	18.21198227636899	3.17602253962837
H	10.86199058422353	21.77534164175857	9.63411802652022
H	9.81742103834945	21.74712590154823	10.94833566814082
H	7.15365504483619	16.73014638935690	14.30509367765470
H	6.85465858632959	18.34811767957778	14.10173174973309

Table S32: DFT optimized coordinates of Model VI - $[NpO_2OH_4]^{2-}$

Np	8.72502635791728	17.45370766596233	8.72546761111251
O	8.74170104142959	15.70108336317365	8.78524237817271
O	8.70815157694908	19.20587927379087	8.66550970963691
O	7.63765319304776	17.41604272650315	6.71697387290233
O	9.81074363625659	17.48090865663642	10.73712905609762
O	6.72236168121516	17.46242664860728	9.81058206093468

O	10.73353513347848	17.44608354459842	7.64464589521863
H	5.89154029315397	17.34896132560744	10.30356464357056
H	11.56353148224829	17.54830872956728	7.14666984976601
H	10.32057331755395	17.66644549892527	11.54272823089754
H	7.13264322166691	17.28107817480045	5.89738946977461
Li	10.63230766570595	15.44119909818474	6.82590364078425
Li	6.82589854229784	15.44119993509974	6.82020622553820
Li	10.63230019028080	15.44120315157480	10.62660414541683
Li	6.82590101817359	15.44120345721865	10.63228170524715
Li	8.72630425173484	13.72640087314258	8.72630071162575
Li	10.63229696411677	19.46380063321657	10.62660315056750
Li	6.82593554901402	19.46377381059950	10.63234545211295
Li	10.62659660278756	19.46379818139808	6.82019942257157
Li	6.82592378926214	19.46376312335812	6.82016694807602
Li	8.72627850889670	21.17861150495516	8.72629308127249
Li	5.24380052811398	17.45249619540839	5.24379817898490
Li	12.20870095608542	17.45250276407420	12.20870460385661
Li	5.24379970803361	17.45249624613480	12.20869795836163
Li	12.20870338224240	17.45250406690162	5.24380197371211
Li	4.88149077427422	17.45250816488516	8.72629765897995
Li	12.57100534051546	17.45249399919375	8.72630579335819
Li	8.72629582866328	17.45250381793468	12.57100055207428
Li	8.72630306607664	17.45249853637394	4.88149999558627
O	5.46090285599451	15.48039605457479	11.99160007972759
O	7.27398294875834	13.64608270264362	10.17860490783687
O	11.99161092724201	15.48039981967080	5.46090775590041
O	10.17851075619644	13.64610082190699	7.27400751298615
O	11.99160093780983	15.48039995652190	11.99160382165076
O	10.17850196391282	13.64610377359591	10.17850442725186
O	5.46090331865506	15.48039390375761	5.46090388040166
O	7.27400508274492	13.64610205572730	7.27399399794318
O	3.76797617840495	17.45251964713729	6.93210634872415
O	13.68451716178831	17.45249388485494	10.52041122211328
O	3.76800081347289	17.45249922960796	10.52040015969036
O	13.68449821167568	17.45251681685351	6.93209027286344
O	6.93209668924784	17.45249600916036	13.68450164693494
O	10.52040487554384	17.45249840542566	3.76800023103660
O	10.52039706053630	17.45251242179964	13.68450256997480
O	6.93211372538478	17.45248613698366	3.76801884353328
O	5.46088421547967	19.42461752628136	5.46091341049504
O	7.27399677203954	21.25889858288207	7.27399835557578
O	11.99159727546389	19.42460578709678	11.99160070035957
O	10.17849997923420	21.25890191444063	10.17850235333728

O	5.46086492171961	19.42461429992761	11.99156278151252
O	7.27398936884851	21.25889978463518	10.17849072646693
O	11.99159730783402	19.42460201355529	5.46090023094947
O	10.17848978167331	21.25889532992358	7.27399752604725
H	5.54357517849885	15.09727753269054	4.54775668233387
H	4.54554671526699	15.17871855512272	5.68612745689726
H	7.37284956890512	18.32834318455934	3.44364548719514
H	6.72279207627716	17.00572110592475	2.93319238225673
H	3.29762626736865	18.27426249871838	7.20888748950369
H	2.96741930898435	16.91181245962131	6.67984575695280
H	5.78958448076088	19.83864071894276	4.61380501221460
H	4.61854350380616	19.76774786235169	5.84039051227414
H	11.06364446340944	21.65646618234761	7.50088741497294
H	9.99981905512498	21.96769309737045	6.62307975201864
H	12.69277112321215	19.97281421307938	5.89439738430910
H	11.90682749989868	19.83177080612203	4.56363065443787
H	12.83538101325675	15.10383943929859	5.76829910206588
H	11.51266844131103	14.80090731090983	4.82935798604469
H	10.94468447648879	13.18071902422492	7.67557334787343
H	10.06763126747477	13.34917638773733	6.26078281325396
H	7.62421369189158	21.70804389103429	6.45813190178642
H	6.60780813418310	21.97509866643995	7.54538751824530
H	7.36778564171110	21.69659100162449	11.12244771308166
H	6.61302868162168	21.85264586314056	9.71807918823615
H	5.77536594817208	20.10579684612538	12.67684840345589
H	4.58129398856983	19.76634152544620	11.71210071614271
H	12.85133651157862	19.79770782437753	11.69510464208504
H	11.88343727663194	19.71715475166328	12.93170703307929
H	13.95259427869278	16.50822634960926	10.24533930513196
H	14.45814629307954	18.02201420368606	10.68996648877433
H	10.07638591761323	16.57489781129585	14.02819871291435
H	10.74339358319395	17.88657316210068	14.52182925214571
H	12.81309165033760	14.99327625133541	11.81618925843661
H	11.52265259554236	15.19938192220045	12.84637375770817
H	14.12750169098737	16.76220024440218	7.67958117244904
H	14.36879102543476	18.12786144603588	6.89943533181291
H	10.92408634879412	13.07087911689492	9.81975412835459
H	9.90993092693377	13.06293220752169	10.93894437889579
H	7.51452450551245	13.05115549511399	10.91020432145110
H	6.52126731270319	13.12906419755187	9.86399480714222
H	5.65685879563264	15.02071462251768	12.83839604263531
H	4.74163357761193	14.95918026774449	11.55564494211433
H	7.53213371424317	13.04305772426565	6.54537028836037

H	6.79925720898462	12.99215275853855	7.94876828745754
H	3.26097375057549	18.16194534733591	9.84543573674932
H	3.04899741345335	16.77459013226810	10.51832695784974
H	10.65292422523829	16.64716737398296	3.21804244924734
H	10.32269935850994	18.21198229222681	3.17602256600849
H	10.86199058012124	21.77534160419969	9.63411801173724
H	9.81742104219998	21.74712587837859	10.94833567676447
H	7.15365506044707	16.73014638607379	14.30509370094513
H	6.85465860910449	18.34811769187484	14.10173176575477

Table S33: DFT optimized coordinates of Model VI - $[NpO_2OH_4]^-$

Np	8.72638375398771	17.45376265016215	8.72618086630578
O	8.73976040892374	15.69966769095905	8.76565592036572
O	8.71123838958457	19.20914629786908	8.68612879865458
O	7.63088034757300	17.43149645510773	6.72184209275857
O	9.82075008774511	17.47401796783790	10.73015248444682
O	6.72893231069433	17.46303331685209	9.81606972268192
O	10.72683262686043	17.45359602462750	7.63829727842274
H	5.89396963022035	17.33684852881474	10.30059267844969
H	11.56416663864783	17.54806595579157	7.14918310908047
H	10.31853864619904	17.65455193732452	11.54464068119363
H	7.13177321110873	17.28232409657662	5.90150338990309
Li	10.63230775058144	15.44119908802696	6.82590373537170
Li	6.82589848608267	15.44119993510845	6.82020622082265
Li	10.63230035367102	15.44120315476930	10.62660405778454
Li	6.82590099020947	15.44120344863260	10.63228167540089
Li	8.72630424358327	13.72640087091181	8.72630066311814
Li	10.63229703923377	19.46380065468452	10.62660303710552
Li	6.82593552662457	19.46377380857578	10.63234540877465
Li	10.62659669580608	19.46379812063120	6.82019948862430
Li	6.82592376666537	19.46376315516251	6.82016694051611
Li	8.72627854536294	21.17861142439932	8.72629299193273
Li	5.24380053407187	17.45249616224119	5.24379816917962
Li	12.20870091269459	17.45250278914214	12.20870466991250
Li	5.24379978360619	17.45249622082316	12.20869804623893
Li	12.20870340052105	17.45250405736172	5.24380199570828
Li	4.88149079191976	17.45250816227385	8.72629770998151
Li	12.57100532587438	17.45249415780926	8.72630573485000
Li	8.72629556228412	17.45250379703905	12.57100030591121
Li	8.72630313718727	17.45249857100742	4.88150028076287
O	5.46090283050053	15.48039605741734	11.99160005625775
O	7.27398299246938	13.64608272467997	10.17860496893206

O	11.99161091983659	15.48039985064243	5.46090770184984
O	10.17851068480875	13.64610083323173	7.27400750889141
O	11.99160090550575	15.48039998659235	11.99160382309668
O	10.17850188323175	13.64610377547396	10.17850447561083
O	5.46090331898220	15.48039388377255	5.46090388788806
O	7.27400510965245	13.64610204353346	7.27399400752687
O	3.76797615428165	17.45251964097490	6.93210633247798
O	13.68451721607108	17.45249375244038	10.52041118930660
O	3.76800083682785	17.45249924237044	10.52040012333346
O	13.68449814546200	17.45251681189948	6.93209027306486
O	6.93209678801162	17.45249603432794	13.68450173534736
O	10.52040483340683	17.45249835773253	3.76800011721866
O	10.52039712063432	17.45251241393850	13.68450273072118
O	6.93211374619198	17.45248614864328	3.76801873620565
O	5.46088422734415	19.42461754332605	5.46091340966390
O	7.27399678838836	21.25889859042820	7.27399838962694
O	11.99159727196093	19.42460576668250	11.99160071441041
O	10.17849990885489	21.25890185615625	10.17850241080676
O	5.46086489310638	19.42461429682420	11.99156276506846
O	7.27398937130061	21.25889989657632	10.17849075197075
O	11.99159728100108	19.42460200313268	5.46090020869512
O	10.17848968397292	21.25889541878698	7.27399753914659
H	5.54357518028099	15.09727754234631	4.54775668020595
H	4.54554672230236	15.17871855986229	5.68612745827156
H	7.37284955481839	18.32834317963446	3.44364548445410
H	6.72279206034745	17.00572110642592	2.93319238040412
H	3.29762627497485	18.27426250106585	7.20888749173511
H	2.96741931411842	16.91181245953682	6.67984575613871
H	5.78958447593482	19.83864071340640	4.61380501100241
H	4.61854350209411	19.76774785751478	5.84039051466442
H	11.06364448244017	21.65646614933076	7.50088740200994
H	9.99981906407079	21.96769308436233	6.62307975140106
H	12.69277112249778	19.97281422197526	5.89439738870002
H	11.90682750562195	19.83177080667008	4.56363065308760
H	12.83538100232527	15.10383943027294	5.76829911300593
H	11.51266844595395	14.80090730320190	4.82935798061117
H	10.94468448955647	13.18071901476714	7.67557332802949
H	10.06763127306926	13.34917637888749	6.26078281049266
H	7.62421368843416	21.70804388799907	6.45813189505578
H	6.60780813017905	21.97509866474421	7.54538750885884
H	7.36778565234756	21.69659095190948	11.12244771835723
H	6.61302867993577	21.85264584651992	9.71807918777341
H	5.77536594413776	20.10579684296945	12.67684840929116

H	4.58129398958905	19.76634153046155	11.71210071256120
H	12.85133650851091	19.79770783565706	11.69510462994564
H	11.88343728529174	19.71715475049402	12.93170703454349
H	13.95259424418211	16.50822636324187	10.24533932989465
H	14.45814628730495	18.02201422217223	10.68996649308497
H	10.07638592018465	16.57489780845108	14.02819870314946
H	10.74339358627303	17.88657316082777	14.52182923936250
H	12.81309164735597	14.99327623187917	11.81618925000763
H	11.52265260635636	15.19938191808313	12.84637376566374
H	14.12750170885798	16.76220023765084	7.67958117384727
H	14.36879103296738	18.12786144551133	6.89943534368903
H	10.92408636514351	13.07087911447299	9.81975414251499
H	9.90993093521812	13.06293220211999	10.93894437318928
H	7.51452449966712	13.05115549166265	10.91020431321516
H	6.52126730159728	13.12906418962397	9.86399480104255
H	5.65685879261841	15.02071462814377	12.83839604138569
H	4.74163357974423	14.95918026322660	11.55564494339266
H	7.53213371732340	13.04305773042153	6.54537028477764
H	6.79925720251633	12.99215275909946	7.94876827358712
H	3.26097374326984	18.16194534183234	9.84543574139864
H	3.04899740725790	16.77459012859726	10.51832696126014
H	10.65292423053878	16.64716738251949	3.21804244450885
H	10.32269935756128	18.21198229953711	3.17602256028932
H	10.86199059344004	21.77534163562109	9.63411803155812
H	9.81742105263351	21.74712589789239	10.94833567399897
H	7.15365505627204	16.73014638761654	14.30509370779724
H	6.85465860567910	18.34811769036745	14.10173177066689

7.0 References

1 Nyman, M., Rodriguez, M. A. & Campana, C. F. Self-Assembly of Alkali-Uranyl-Peroxide Clusters. *Inorganic Chemistry* **49**, 7748-7755 (2010). https://doi.org:10.1021/ic1005192

2 Dolomanov, O. V., Bourhis, L. J., Gildea, R. J., Howard, J. A. K. & Puschmann, H. OLEX2: a complete structure solution, refinement and analysis program. *Journal of Applied Crystallography* **42**, 339-341 (2009). https://doi.org:doi:10.1107/S0021889808042726

3 Neese, F., Wennmohs, F., Becker, U. & Riplinger, C. The ORCA quantum chemistry program package. *The Journal of Chemical Physics* **152** (2020). https://doi.org:10.1063/5.0004608

4 Becke, A. D. Density-functional thermochemistry. III. The role of exact exchange. *The Journal of Chemical Physics* **98**, 5648-5652 (1993). https://doi.org:10.1063/1.464913

5 Lee, C., Yang, W. & Parr, R. G. Development of the Colle-Salvetti correlation-energy formula into a functional of the electron density. *Physical Review B* **37**, 785-789 (1988). https://doi.org:10.1103/PhysRevB.37.785

6 Lenthe, E. v., Baerends, E.-J. & Snijders, J. G. Relativistic regular two-component Hamiltonians. *The Journal of chemical physics* **99**, 4597-4610 (1993).

7 van Wüllen, C. Molecular density functional calculations in the regular relativistic approximation: Method, application to coinage metal diatomics, hydrides, fluorides and chlorides, and comparison with first-order relativistic calculations. *The Journal of chemical physics* **109**, 392-399 (1998).

8 Pantazis, D. A., Chen, X.-Y., Landis, C. R. & Neese, F. All-electron scalar relativistic basis sets for third-row transition metal atoms. *Journal of chemical theory and computation* **4**, 908-919 (2008).

9 Weigend, F. & Ahlrichs, R. Balanced basis sets of split valence, triple zeta valence and quadruple zeta valence quality for H to Rn: Design and assessment of accuracy. *Physical Chemistry Chemical Physics* **7**, 3297-3305 (2005).

10 Pantazis, D. A. & Neese, F. All-Electron Scalar Relativistic Basis Sets for the Actinides. *Journal of Chemical Theory and Computation* **7**, 677-684 (2011). https://doi.org:10.1021/ct100736b

11 Grimme, S., Antony, J., Ehrlich, S. & Krieg, H. A consistent and accurate ab initio parametrization of density functional dispersion correction (DFT-D) for the 94 elements H-Pu. *The Journal of Chemical Physics* **132** (2010). https://doi.org:10.1063/1.3382344

12 Grimme, S., Ehrlich, S. & Goerigk, L. Effect of the damping function in dispersion corrected density functional theory. *Journal of Computational Chemistry* **32**, 1456-1465 (2011). https://doi.org:https://doi.org/10.1002/jcc.21759

13 Barone, V. & Cossi, M. Quantum Calculation of Molecular Energies and Energy Gradients in Solution by a Conductor Solvent Model. *The Journal of Physical Chemistry A* **102**, 1995-2001 (1998). https://doi.org:10.1021/jp9716997

14 Klamt, A. Conductor-like Screening Model for Real Solvents: A New Approach to the Quantitative Calculation of Solvation Phenomena. *The Journal of Physical Chemistry* **99**, 2224-2235 (1995). https://doi.org:10.1021/j100007a062

15 Chemcraft v. Version 1.8 (2009).

16 Lu, T. & Chen, F. Multiwfn: A multifunctional wavefunction analyzer. *Journal of Computational Chemistry* **33**, 580-592 (2012). https://doi.org:https://doi.org/10.1002/jcc.22885
